# Out-of-plane coordination of iridium single atoms with organic molecules and cobalt–iron hydroxides to boost oxygen evolution reaction

**DOI:** 10.1038/s41565-024-01807-x

**Published:** 2024-10-21

**Authors:** Jie Zhao, Yue Guo, Zhiqi Zhang, Xilin Zhang, Qianqian Ji, Hua Zhang, Zhaoqi Song, Dongqing Liu, Jianrong Zeng, Chenghao Chuang, Erhuan Zhang, Yuhao Wang, Guangzhi Hu, Muhammad Asim Mushtaq, Waseem Raza, Xingke Cai, Francesco Ciucci

**Affiliations:** 1 Institute for Advanced Study, Shenzhen University, Shenzhen, China; 2 School of Energy and Environment, City University of Hong Kong, Hong Kong, China; 3 Department of Mechanical Engineering and Research Institute for Smart Energy, The Hong Kong Polytechnic University, Hong Kong, China; 4 Key Laboratory of Energy Thermal Conversion and Control (Ministry of Education), School of Energy and Environment, Southeast University, Nanjing, China; 5 School of Physics, Henan Key Laboratory of Photovoltaic Materials, Henan Normal University, Xinxiang, China; 6 School of Ecology and Environmental Science, Yunnan University, Kunming, China; 7 College of Mechatronics and Control Engineering, Shenzhen University, Shenzhen, China; 8 Shanghai Synchrotron Radiation Facility, Shanghai Advanced Research Institute, Chinese Academy of Sciences, Shanghai, China; 9 Department of Physics, Tamkang University, New Taipei City, Taiwan; 10 Future Battery Research Center, Global Institute of Future Technology, Shanghai Jiao Tong University, Shanghai, China; 11 Department of Mechanical and Aerospace Engineering, The Hong Kong University of Science and Technology, Hong Kong, China; 12 University of Bayreuth, Chair of Electrode Design for Electrochemical Energy Systems, Bayreuth, Germany; 13 University of Bayreuth, Bavarian Center for Battery Technology (BayBatt), Bayreuth, Germany

**Keywords:** Electrocatalysis, Two-dimensional materials, Electrocatalysis, Two-dimensional materials, Electrocatalysis

## Abstract

Advancements in single-atom-based catalysts are crucial for enhancing oxygen evolution reaction (OER) performance while reducing precious metal usage. A comprehensive understanding of underlying mechanisms will expedite this progress further. Here we report Ir single atoms coordinated out-of-plane with dimethylimidazole (MI) on CoFe hydroxide (Ir_1_/(Co,Fe)-OH/MI). This Ir_1_/(Co,Fe)-OH/MI catalyst, which was prepared using a simple immersion method, delivers ultralow overpotentials of 179 mV at a current density of 10 mA cm^−2^ and 257 mV at 600 mA cm^−2^ as well as an ultra-small Tafel slope of 24 mV dec^−1^. Furthermore, Ir_1_/(Co,Fe)-OH/MI has a total mass activity exceeding that of commercial IrO_2_ by a factor of 58.4. Ab initio simulations indicate that the coordination of MI leads to electron redistribution around the Ir sites. This causes a positive shift in the *d*-band centre at adjacent Ir and Co sites, facilitating an optimal energy pathway for OER.

## Abstract (web summary)

This article presents a new method for coordinating iridium atoms with dimethylimidazole and cobalt–iron hydroxides. This enhances the oxygen evolution reaction and delivers high current densities with reduced precious metal use.

## Main

Electrochemical water splitting powered by renewable electricity is a promising green pathway to realizing large-scale hydrogen production^1,2^. One of the major bottlenecks of water splitting is the oxygen evolution reaction (OER), which is a sluggish reaction involving the transfer of four electrons^3^. As the breaking of O–H bonds and the formation of subsequent O–O bonds are required, significant overpotentials are needed to achieve sufficiently high current densities^4^. OER can be speeded up if effective electrocatalysts containing precious metals are used. For instance, commercial OER catalysts are materials based on IrO_2_ or RuO_2_ (refs. ^5,6^). However, these catalysts are far from ideal owing to their high costs linked to high precious material loading, limited stability and significant overpotentials. These factors collectively constrain the commercial viability of this technology^6,7^.

Over the past two decades, researchers have extensively studied high-performance OER catalysts, focusing on materials containing early-transition metals such as metal oxyhydroxides/hydroxides^8,9^, perovskite oxides^10^, metal phosphides^11^, metal nitrides^12^, metal sulfides^13^, metal oxides^14^, metal borides^15^ and metal carbides^4^. Co-, Fe- and Ni-based metal oxyhydroxides/hydroxides have emerged as particularly promising candidates owing to their cost-effectiveness and strong OER activity^16–19^. For instance, NiFe, CoFe and NiCo oxyhydroxides/hydroxides show promising OER overpotentials; the reported values in the 220–350 mV (at 10 mA cm^−2^) range^19–21^ are comparable to those of commercial IrO_2_/RuO_2_ (300–380 mV at 10 mA cm^−2^)^20,21^. Although these Co/Fe/Ni-based oxyhydroxides/hydroxides have relativity low OER overpotentials, they are still far from the desired target (that is, overpotentials below 200 mV at 10 mA cm^−2^)^22^. Several strategies have been used to lower the overpotentials of metal oxyhydroxides/hydroxides, including morphology regulation^23^, defect engineering^24^, creation of heterogeneous interfaces^25^, doping with multiple elements^26^ and single-atom loading^27–29^.

Loading noble-metal single atoms onto metal oxyhydroxides/hydroxides has proven effective in reducing OER overpotentials to values below 200 mV (refs. ^27–29^). For example, Li et al. developed CoFe hydroxides loaded with atomically dispersed Ru atoms using a simple chemical precipitation method, achieving an OER overpotential of only 198 mV (ref. ^27^). Zhai et al. incorporated Ru single atoms into defect-rich NiFe hydroxides via an electrodeposition-etching method, further reducing the OER overpotential to 189 mV (ref. ^28^). Mu et al. used a hydrothermal-soaking process to create hole-rich CoFe hydroxides with atomically dispersed Ru single atoms, achieving OER overpotentials as low as 194 mV (ref. ^29^). Further research is needed to fully exploit the interplay between single-atom coordination, bonding and catalytic performance.

Researchers in the field of single-atom catalysts have used out-of-plane ligand coordination (axial coordination) to enhance the electrocatalytic activity of various electrochemical reactions^30–32^. For instance, Zhang et al. designed a Pt single-atom catalyst on NiFe hydroxide with chloride coordination using an irradiation-impregnation procedure, which improved water dissociation and increased hydrogen production in alkaline media^30^. Similarly, Liu et al. enhanced OER performance by coordinating out-of-plane, a phosphate group to a CoN_4_ site (the CoN_4_ site is a single Co atom surrounded by four planar nitrogen atoms)^32^. These studies demonstrate the power of out-of-plane ligand coordination engineering to optimize the performance of single-atom catalysts. Applying this technique to noble-metal single atoms on metal oxyhydroxides/hydroxides can significantly improve OER performance. However, no studies have explored this promising avenue of research in the context of OER.

Herein we prepared Ir single atoms out-of-plane coordinated with dimethylimidazole (MI) on CoFe hydroxide nanosheets (denoted as Ir_1_/(Co,Fe)-OH/MI). As an OER catalyst, the as-prepared Ir_1_/(Co,Fe)-OH/MI showed impressively low overpotential, small Tafel slope, high areal activity (based on electrochemically active surface area (ECSA)) and mass activity (based on total catalyst mass). First-principle simulations suggest that this excellent OER performance stems from the unique coordination between the Ir single atoms and MI. This coordination effectively redistributed charge around the Ir sites, which favourably shifted the *d*-band centres of both Ir and adjacent Co atoms and reduced the reaction energy barrier. When used as overall water splitting electrodes, the Ir_1_/(Co,Fe)-OH/MI exhibited exciting application potential.

## Synthesis and characterization

**Fig. 1:**
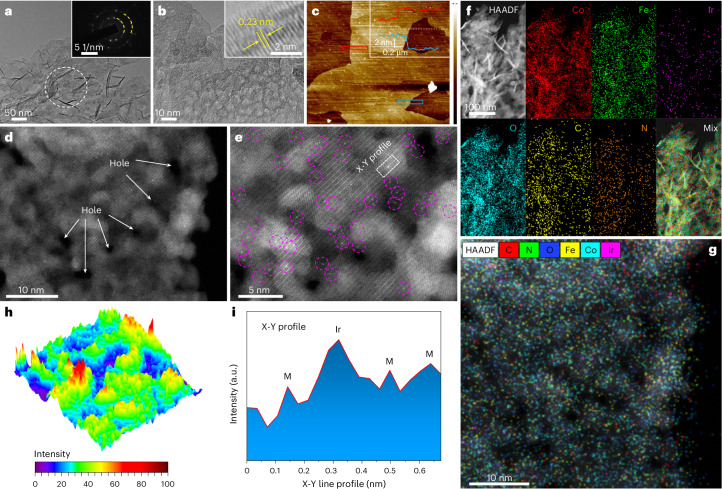
Morphology characterizations of the Ir_1_/(Co,Fe)-OH/MI sample. **a**, TEM micrograph with a corresponding SAED image. **b**, Integrated TEM and HR-TEM images. **c**, AFM characterization. **d**,**e**, HAADF-STEM images. Holes (**d**). Ir single atoms (**e**). **f**, TEM image with corresponding elemental maps. **g**, High-resolution elemental map. **h**, Three-dimensional atom-overlapping intensity value representation. **i**, Intensity profile along the dashed rectangle in **e**. Note: M in **i** represents either Co or Fe. Source data

Ir_1_/(Co,Fe)-OH/MI was prepared using a process that converted the Co-based complex (Co-MI) into CoFe hydroxide. As illustrated in Supplementary Scheme 1, this synthesis procedure required two steps. The synthesis of the Ir_1_/(Co,Fe)-OH/MI sample started with the growth of Co-MI on nickel foam using an aqueous solution containing Co^2+^ ions and MI molecules. The resulting Co-MI featured petal-shaped sheets characterized by nanometre-sized pores (Supplementary Fig. 1). As a second step, Co-MI was converted into Ir_1_/(Co,Fe)-OH/MI following immersion in an ethylene glycol/water solution containing Co^2+^, Fe^3+^ and Ir^3+^ (Supplementary Figs. 2−7 and Table 1). For a more detailed description of the synthesis procedure, refer to ‘Synthesis of Ir_1_/(Co,Fe)-OH/MI’ in Methods and ‘Synthesis process for Ir_1_/(Co,Fe)-OH/MI’ section in Supplementary Information. The morphology of Ir_1_/(Co,Fe)-OH/MI was characterized using transmission electron microscopy (TEM) combined with selected area electron diffraction (SAED) and high-resolution TEM (HR-TEM) (Fig. 1). The TEM images indicate that Ir_1_/(Co,Fe)-OH/MI has a nanosheet morphology characterized by the presence of many holes, similar to that of Co-MI (Fig. 1a,b and Supplementary Fig. 1). Additional SAED image indicates that Ir_1_/(Co,Fe)-OH/MI is polycrystalline (Fig. 1a). The HR-TEM image indicates a 0.23 nm lattice fringe of CoFe hydroxide in Ir_1_/(Co,Fe)-OH/MI (Fig. 1b). Atomic force microscopy (AFM) analysis suggests that the thickness of the Ir_1_/(Co,Fe)-OH/MI nanosheet is 3–4 nm (Fig. 1c). Aberration-corrected high angle annular dark field-scanning transmission electron microscopy (HAADF-STEM) images highlight the presence of isolated Ir atoms (bright dots marked by pink circles in Fig. 1e), which were dispersed on the surface of (Co,Fe)-OH (Fig. 1d,e and Supplementary Fig. 8). Elemental mapping images show that the dispersion of Co, Fe, Ir, O, C and N atoms (with C and N originating from MI) was uniform across the entire Ir_1_/(Co,Fe)-OH/MI sample (Fig. 1f,g and Supplementary Figs. 9 and 10). Analysis of atom-overlapping intensity values further confirms atomic dispersion of Ir on (Co,Fe)-OH (Fig. 1h and Supplementary Fig. 11 with the red regions indicating Ir)^14^. To confirm isolated Ir atoms, the intensity profile in Fig. 1i (from the dashed rectangle in Fig. 1e) shows a prominent Ir peak, visually supporting atomic dispersion. Inductively coupled plasma mass spectrometry (ICP-MS) confirmed 0.33 wt% Ir loading (Supplementary Table 2). Electrodeposited Ir_1_/(Co,Fe)-OH without MI also showed similar atomically dispersed Ir within porous nanosheets (Supplementary Fig. 12), with 0.30 wt% Ir loading (Supplementary Table 3). For comparison, Ir-free (Co,Fe)-OH/MI and (Co,Fe)-OH were prepared and characterized by TEM (Supplementary Figs. 13 and 14).

**Fig. 2:**
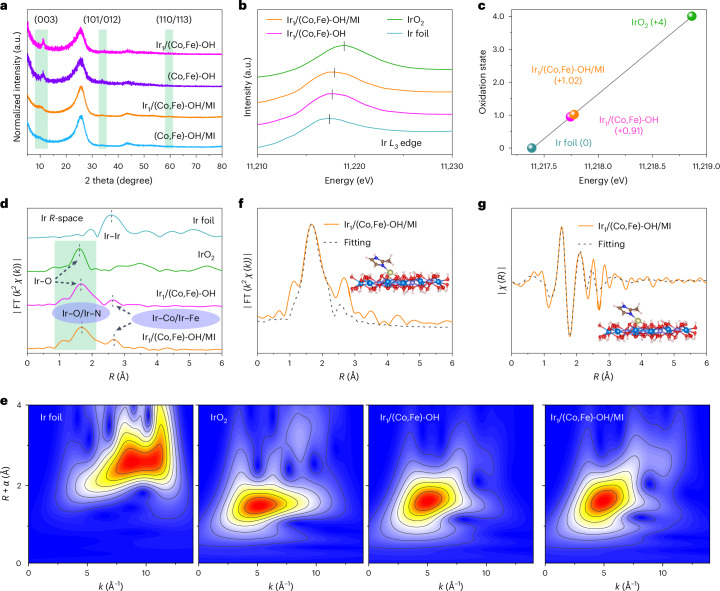
Structural characterizations of Ir_1_/(Co,Fe)-OH/MI and Ir_1_/(Co,Fe)-OH samples. **a**, XRD patterns. **b**, XANES spectra. **c**, Valence of various Ir species obtained from Ir *L*_3_-edge XANES. **d**, Normalized Ir *L*_3_-edge FT-EXAFS spectra. **e**, Wavelet-transformed spectra of Ir *L*_3_-edge FT-EXAFS. **f**,**g**, Fitting results of the FT-EXAFS spectra of Ir_1_/(Co,Fe)-OH/MI. Real part (**f**). Imaginary part (**g**). Source data

Further structural characterizations are presented in Fig. 2. The X-ray powder diffraction (XRD) patterns of Ir-containing catalysts closely resemble their Ir-free counterparts, confirming the absence of Ir nanoparticles or bulk Ir metal (Fig. 2a and Supplementary Fig. 15). Characteristic layered double hydroxide peaks in the XRD patterns identify CoFe hydroxides as the supports for Ir single atoms^33–35^. X-ray absorption spectroscopy (XAS) was used to probe the electronic structure and local atomic environment of Ir. The Ir *L*_3_-edge X-ray absorption near edge structure (XANES) spectra for both Ir_1_/(Co,Fe)-OH/MI and Ir_1_/(Co,Fe)-OH show similar peaks, shifted ~0.4 eV higher than Ir foil but lower than IrO_2_, suggesting an Ir oxidation state between 0 and +4 (refs. ^14^^,^^36^) (Fig. 2b and Supplementary Fig. 16). Linear regression analysis of the first-order derivative zero further refines the Ir oxidation states to +1.02 and +0.91 for Ir_1_/(Co,Fe)-OH/MI and Ir_1_/(Co,Fe)-OH, respectively (Fig. 2c and Supplementary Fig. 17). The slightly higher Ir oxidation state in Ir_1_/(Co,Fe)-OH/MI may be due to partial coordination of Ir with MI.

The Fourier-transformed extended X-ray absorption fine structure (FT-EXAFS) spectra reveal the nature of Ir bonds in Ir_1_/(Co,Fe)-OH/MI, Ir_1_/(Co,Fe)-OH, IrO_2_ and Ir foil (Fig. 2d). In *R*-space, IrO_2_ and Ir foil exhibit single peaks at 1.60 Å and 2.60 Å, corresponding to Ir–O and Ir–Ir bonds, respectively. Ir_1_/(Co,Fe)-OH shows two peaks at 1.65 Å and 2.63 Å, attributed to Ir–O and Ir–Co/Ir–Fe. By contrast, Ir_1_/(Co,Fe)-OH/MI shows a slightly shifted profile with peaks at 1.68 Å (Ir–O or Ir–N) and 2.68 Å (Ir–Co or Ir–Fe), implying an impact of MI coordination. The wavelet-transformed Ir *L*_3_-edge EXAFS spectra of Ir_1_/(Co,Fe)-OH/MI and Ir_1_/(Co,Fe)-OH exhibit distinct differences compared with IrO_2_ and Ir foil (Fig. 2e), further supporting the atomic dispersion of Ir species on CoFe hydroxides, with no evidence of Ir clusters or nanoparticles. EXAFS analysis and structural simulations reveal the coordination structure of Ir to be Ir–CoFe for Ir_1_/(Co,Fe)-OH and Ir(N)–CoFe for Ir_1_/(Co,Fe)-OH/MI (Fig. 2f,g and Supplementary Figs. 18−20). Notably, in Ir_1_/(Co,Fe)-OH/MI, the MI ligand coordinates out-of-plane with the Ir single atom.

## OER performance

**Fig. 3:**
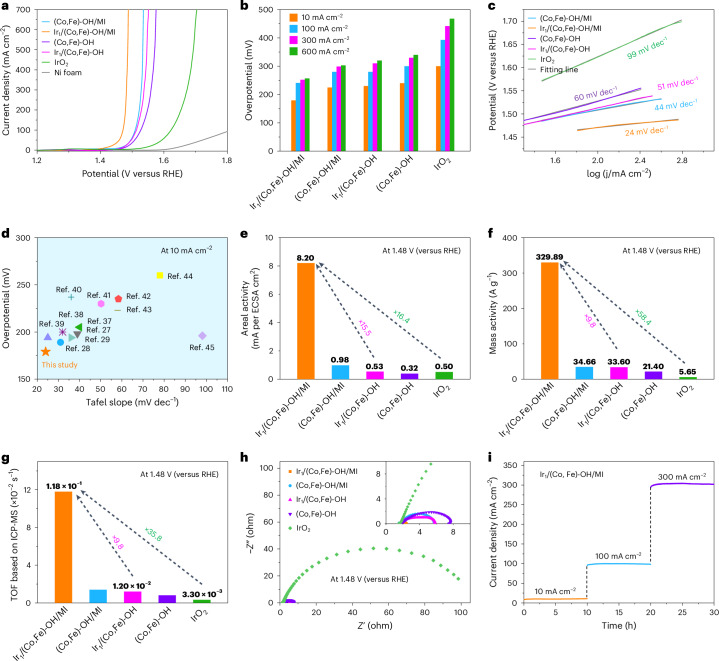
Electrochemical performances for OER. **a**, LSV curves in 1 M KOH at a scanning rate of 5 mV s^−1^. **b**, Overpotentials at 10 mA cm^−2^, 100 mA cm^−2^, 300 mA cm^−2^ and 600 mA cm^−2^. **c**, Tafel plots. **d**, Comparison of overpotentials and Tafel plots in reported hydroxide-based noble-metal single-atom OER electrocatalysts. **e**, Areal activity based on ECSA. **f**, Activity normalized relative to the catalyst mass. **g**, TOF from ICP-MS characterization. **h**, Electrochemical impedance spectroscopy. **i**, Chronoamperometry measurements of Ir_1_/(Co,Fe)-OH/MI at 10 mA cm^−2^, 100 mA cm^−2^ and 300 mA cm^−2^. Source data

To investigate the impact of MI–Ir coordination on OER performance, we evaluated the prepared samples electrochemically in 1 M KOH (Fig. 3). The linear sweep voltammetry (LSV) results demonstrate high current densities (up to 700 mA cm^−2^) and excellent OER activity across all catalysts. Notably, Ir_1_/(Co,Fe)-OH/MI exhibits the highest activity, followed by (Co,Fe)-OH/MI, Ir_1_/(Co,Fe)-OH and (Co,Fe)-OH, all outperforming IrO_2_ and Ni foam (Fig. 3a). At 10 mA cm^−2^, 100 mA cm^−2^, 300 mA cm^−2^ and 600 mA cm^−2^, the overpotentials of Ir_1_/(Co,Fe)-OH/MI are 179 mV, 241 mV, 252 mV and 257 mV, respectively. These values are notably lower than those observed for (Co,Fe)-OH/MI, Ir1/(Co,Fe)-OH, (Co,Fe)-OH and IrO2 at the same current densities (Fig. 3b). The Tafel slope of Ir_1_/(Co,Fe)-OH/MI is 24 mV dec^−1^, significantly lower than the other catalysts (Fig. 3c). Moreover, the low overpotential (179 mV at 10 mA cm^−2^) and Tafel slope (24 mV dec^−1^) of Ir_1_/(Co,Fe)-OH/MI outperform many reported hydroxide-based noble-metal single-atom electrocatalysts and other state-of-the-art OER catalysts^27–29,37–45^ (Fig. 3d and Supplementary Tables 4 and 5). The Faraday efficiency exceeds 98%, indicating near-theoretical O_2_ production during OER (Supplementary Fig. 21).

The catalyst showcased exceptional performance in both areal activity (based on ECSA) and mass activity (based on total catalyst mass) (Fig. 3e,f, Supplementary Figs. 22 and 23, and Table 6). At an overpotential of 250 mV, Ir_1_/(Co,Fe)-OH/MI achieved an areal activity of 8.20 mA cm^−2^, surpassing Ir_1_/(Co,Fe)-OH and commercial IrO_2_ by factors of 15.5 and 16.4, respectively (Fig. 3e). Furthermore, Ir_1_/(Co,Fe)-OH/MI showed a mass activity of 329.89 A g^−1^, exceeding Ir_1_/(Co,Fe)-OH and IrO_2_ by factors of 9.8 and 58.4, respectively (Fig. 3f). When normalized to Ir mass, Ir_1_/(Co,Fe)-OH/MI delivered an impressive 10^5^ A g_Ir_^−1^, outperforming Ir_1_/(Co,Fe)-OH by a factor of 8.9 and IrO_2_ by a remarkable 15,000-fold margin (Supplementary Fig. 24 and Table 7). The turnover frequency (TOF) of Ir_1_/(Co,Fe)-OH/MI significantly surpassed that of other catalysts, outperforming Ir_1_/(Co,Fe)-OH and IrO_2_ by factors of 9.8 and 35.8, respectively (Fig. 3g and Supplementary Tables 2, 3, 8 and 9). In addition, Ir_1_/(Co,Fe)-OH/MI exhibited the lowest charge transfer resistance among the prepared catalysts (Fig. 3h and Supplementary Table 10). Chronoamperometry tests confirmed the catalyst’s stability, with no significant degradation observed over 10 h at various current densities (10 mA cm^−2^, 100 mA cm^−2^ and 300 mA cm^−2^) (Fig. 3i). Multistep chronopotentiometry consistently supported this stability data across a broad current density range (50–400 mA cm^−2^) (Supplementary Fig. 25). Remarkably, Ir_1_/(Co,Fe)-OH/MI maintained stable operation for over 120 h as evidenced by chronoamperometric and chronopotentiometric measurements (Supplementary Fig. 26) as well as post-mortem morphological, chemical and structural characterizations (Supplementary Figs. 27−31).

## Density functional theory calculations

To elucidate the OER mechanisms in Ir_1_/(Co,Fe)-OH and Ir_1_/(Co,Fe)-OH/MI, we constructed Ir–CoFe and Ir(N)–CoFe atomic models, respectively, based on modified Co hydroxide single-crystal structures^46^ (Supplementary Fig. 20). Density functional theory (DFT) simulations on these models (Supplementary Figs. 32−37 and ‘DFT software, modules and functions’ in Supplementary Information) revealed distinct OER pathways. In the Ir–CoFe model, only the Ir site showed stable adsorption of OER intermediates (^*^OH, ^*^O and ^*^OOH), suggesting that OER primarily occurs at Ir, not adjacent Co or Fe sites (Supplementary Figs. 32−34). Conversely, in the Ir(N)–CoFe model, favourable OER energetics were observed at both Ir and adjacent Co sites, while Fe sites were unfavourable owing to unstable intermediate formation (Supplementary Figs. 35−37).

**Fig. 4:**
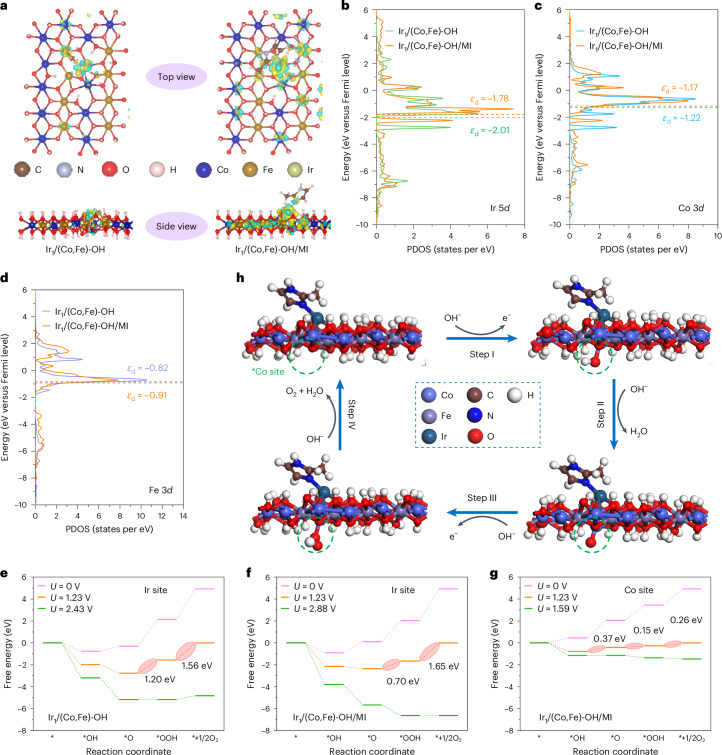
DFT calculations. **a**, Differential charge density of catalysts, where the yellow and green regions represent electronic accumulation and depletion, respectively. **b**, PDOS of Ir sites. **c**, PDOS of Co sites. **d**, PDOS of Fe sites. **e**, Gibbs free energy diagram of the Ir site in Ir_1_/(Co,Fe)-OH. **f**, Gibbs free energy diagram of the Ir site in Ir_1_/(Co,Fe)-OH/MI. **g**, Gibbs free energy diagram of the Co site adjacent to Ir in Ir_1_/(Co,Fe)-OH/MI. **h**, Proposed 4e^−^ mechanism of OER at the Co site adjacent to Ir in Ir_1_/(Co,Fe)-OH/MI. Note: the asterisk denotes the active site for adsorption. Source data

We analysed differential charge density, Bader charge, partial density of states (PDOS) and Gibbs free energies for both Ir–CoFe and Ir(N)–CoFe models (Fig. 4 and Supplementary Figs. 38−41). The Ir(N)–CoFe model exhibited significant charge redistribution around the Ir site owing to out-of-plane coordination with MI (Fig. 4a). Bader charge analysis revealed a greater electron loss at the Ir site for Ir(N)–CoFe (−0.53 e) compared with Ir–CoFe (−0.22 e), indicating a higher Ir valence in Ir(N)–CoFe. This aligns with the XANES-estimated Ir valence of +1.02 for Ir_1_/(Co,Fe)-OH/MI and +0.91 for Ir_1_/(Co,Fe)-OH (Fig. 2c and Supplementary Fig. 41). Similarly, Co or Fe sites adjacent to Ir in Ir(N)–CoFe showed greater electron loss than in Ir–CoFe, suggesting increased Co or Fe valence in Ir_1_/(Co,Fe)-OH/MI compared with Ir_1_/(Co,Fe)-OH (Supplementary Figs. 41−44). To examine the impact of the electronic structure on the adsorption of OER intermediates, we calculated the corresponding PDOS of Ir, Co and Fe sites. The Ir(N)–CoFe model showed higher *d*-band centres for both Ir (−1.78 eV) and its adjacent Co (−1.17 eV) compared with their counterparts in the Ir–CoFe model (−2.01 eV at Ir and −1.22 eV at Co). Conversely, the adjacent Fe site in Ir(N)–CoFe exhibited a lower *d*-band centre (−0.91 eV) than in Ir–CoFe (−0.82 eV) (Fig. 4b−d). On the basis of the *d*-band centre model, the elevated *d*-band centres at Ir and adjacent Co in Ir(N)–CoFe suggest more unoccupied orbitals, implying stronger adsorption of OER intermediates at these sites compared with the Ir–CoFe model^47,48^.

To understand the OER process at Ir and adjacent Co sites in Ir_1_/(Co,Fe)-OH/MI and Ir_1_/(Co,Fe)-OH, the Gibbs free energies (*G*) of adsorbed OER intermediates for the Ir(N)–CoFe and Ir–CoFe models were calculated^49^ (Fig. 4e−g). In alkaline electrolytes, OER follows four steps^28^: * → *OH, *OH → *O, *O → *OOH and *OOH → * + ½O_2_. At Ir sites, only the first step (* → *OH) was calculated to be spontaneous (Δ*G* < 0) at 0 V for both models. However, Ir(N)–CoFe exhibits a more negative Δ*G* for the first step than Ir–CoFe, indicating stronger OH adsorption on Ir_1_/(Co,Fe)-OH/MI (Fig. 4e,f). At 1.23 V, both models exhibit two spontaneous (that is, * → *OH and *OH → *O) and two unfavourable (that is, *O → *OOH and *OOH → * + ½O_2_) steps. The energy barriers for the unfavourable steps are lower for Ir(N)–CoFe (2.35 eV) compared with Ir–CoFe (2.76 eV), suggesting higher electrocatalytic activity for the former (Fig. 4e,f). Increasing the applied potential drives the transition of non-spontaneous steps (*O → *OOH and OOH → * + ½O_2_) towards spontaneity. Specifically, in Ir–CoFe, the *O → *OOH step becomes thermodynamically favourable at 2.43 V (Fig. 4e). By contrast, Ir(N)–CoFe exhibits favourable energetics for both *O → OOH and OOH → * + ½O_2_ at 2.88 V (Fig. 4f). These simulations indicate that the Ir site of Ir_1_/(Co,Fe)-OH/MI has enhanced OER activity compared with that of Ir_1_/(Co,Fe)-OH.

At the Co site adjacent to Ir in the Ir(N)–CoFe model, the OER process is non-spontaneous at 0 V (Fig. 4g). At 1.23 V, one step (that is, * → *OH) is spontaneous, while three steps (that is, *OH → *O, *O → *OOH and *OOH → * + ½O_2_) are not (Fig. 4g). Importantly, the total energy barrier at this Co site adjacent to Ir is only 0.78 eV, a value lower than that computed for the Ir site (2.35 eV for Ir(N)–CoFe and 2.76 eV for Ir–CoFe), indicating a more efficient OER pathway around Co than the Ir site (Fig. 4e−g). Upon increasing the potential to 1.59 V, the previously three non-spontaneous steps at the Co site become spontaneous, implying greater OER reactivity (lower overpotentials) at this site relative to the Ir site (Fig. 4f,g). Correspondingly, the 4e^−^ mechanism for OER at the Co site is outlined in Fig. 4h. The lower computed overpotential for the model corresponding to Ir_1_/(Co,Fe)-OH/MI relative to the one for Ir_1_/(Co,Fe)-OH is consistent with the trend observed experimentally (Fig. 3b and Supplementary Fig. 45).

In summary, out-of-plane coordination of Ir atoms with MI molecules increases the oxidation states of Ir (+1.02) and Co (+2.11) compared with Ir_1_/(Co,Fe)-OH (+0.91 for Ir and +2.05 for Co) (Fig. 2c and Supplementary Fig. 42). This increase aligns with the reduced charge density around Ir and Co sites, as evidenced by the analysis of the differential and Bader charges (Fig. 4a and Supplementary Figs. 38−41). These changes induce a positive shift in the *d*-band centres of Ir and adjacent Co sites (Fig. 4b,c), promoting adsorption of OER intermediates (Fig. 4e–g) and enhancing catalytic performance. For instance, the Ir_1_/(Co,Fe)-OH/MI catalyst exhibits lower overpotentials and Tafel slope (Fig. 3a–d) relative to Ir_1_/(Co,Fe)-OH. Moreover, its activity normalized by Ir mass exceeds that of commercial IrO_2_ by more than four orders of magnitude (Supplementary Fig. 24). These findings indicate that out-of-plane coordination with MI molecules is a suitable approach for tuning electronic structure and improving OER activity in noble-metal single-atom catalysts on metal oxyhydroxide/hydroxide supports.

## Electrochemical performance of overall water splitting

**Fig. 5:**
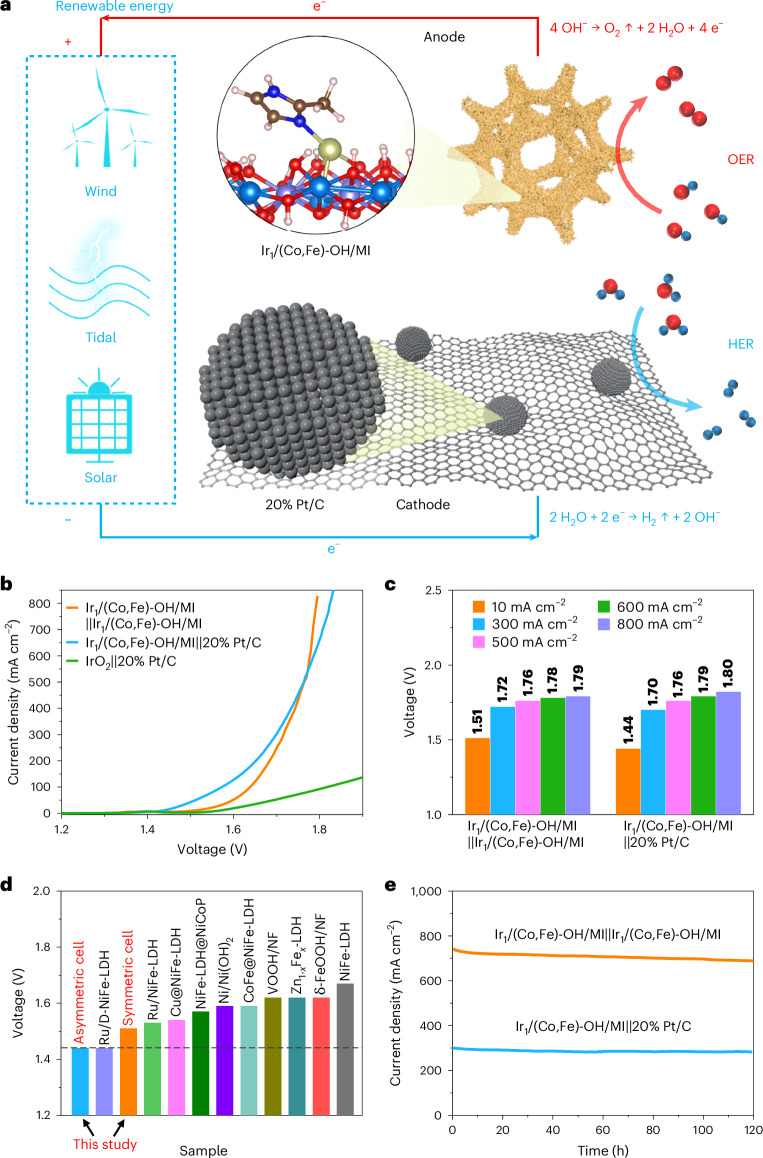
Overall water splitting performance. **a**, Schematic diagram of water splitting. **b**, LSV curves in 1 M KOH at a scanning rate of 5 mV s^−1^. **c**, Water splitting voltage at 10 mA cm^−2^, 300 mA cm^−2^, 500 mA cm^−2^, 600 mA cm^−2^ and 800 mA cm^−2^. **d**, Comparison of water splitting voltage (10 mA cm^−2^) in reported hydroxide-based OER electrocatalysts. **e**, Chronoamperometry measurements at 300 mA cm^−2^ and 700 mA cm^−2^ for 120 h. Source data

To assess the practical feasibility of the Ir_1_/(Co,Fe)-OH/MI catalyst, we evaluated its overall water splitting performance in a two-electrode cell (Fig. 5). The asymmetric Ir_1_/(Co,Fe)-OH/MI||20% Pt/C cell exhibited superior performance below 500 mA cm^−2^ compared with both the symmetric Ir_1_/(Co,Fe)-OH/MI||Ir_1_/(Co,Fe)-OH/MI cell and the asymmetric IrO_2_||20% Pt/C cell (Fig. 5a,b and Supplementary Fig. 46). However, the symmetric cell excelled at current densities above 500 mA cm^−2^ (Fig. 5b). Specifically, at 10 mA cm^−2^, 300 mA cm^−2^ and 500 mA cm^−2^, the asymmetric Ir_1_/(Co,Fe)-OH/MI||20% Pt/C cell achieved water splitting voltages of 1.44 V, 1.70 V and 1.76 V, respectively, outperforming the symmetric Ir_1_/(Co,Fe)-OH/MI||Ir_1_/(Co,Fe)-OH/MI cell (Fig. 5c). At higher current densities (600 mA cm^−2^ and 800 mA cm^−2^), the asymmetric Ir_1_/(Co,Fe)-OH/MI||20% Pt/C cell reached 1.79 V and 1.80 V, while the symmetric Ir_1_/(Co,Fe)-OH/MI||Ir_1_/(Co,Fe)-OH/MI cell showed slightly lower voltages of 1.78 V and 1.79 V. This Ir_1_/(Co,Fe)-OH/MI catalyst shows high overall water splitting performance compared with reported OER electrocatalysts (Fig. 5d, Supplementary Fig. 47 and Tables 11 and 12). The asymmetric Ir_1_/(Co,Fe)-OH/MI||20% Pt/C cell operated stably for 120 h at 300 mA cm^−2^, and the symmetric Ir_1_/(Co,Fe)-OH/MI||Ir_1_/(Co,Fe)-OH/MI cell functioned for 120 h at 700 mA cm^−2^ (Fig. 5e). Even in a two-electrode flow cell, the symmetric Ir_1_/(Co,Fe)-OH/MI||Ir_1_/(Co,Fe)-OH/MI cell maintained stability for 100 h at 800 mA cm^−2^ (Supplementary Fig. 48). To further demonstrate practical applicability, an anion exchange membrane (AEM) water electrolyser using Ir_1_/(Co,Fe)-OH/MI as both anode and cathode exhibited a lower voltage at 500 mA cm^−2^ than an IrO_2_||20% Pt/C electrolyser (Supplementary Fig. 49 and Table 13). Impressively, this electrolyser operated stably for over 150 h at 500 mA cm^−2^ with negligible degradation.

## Generalizing the preparation method

**Fig. 6:**
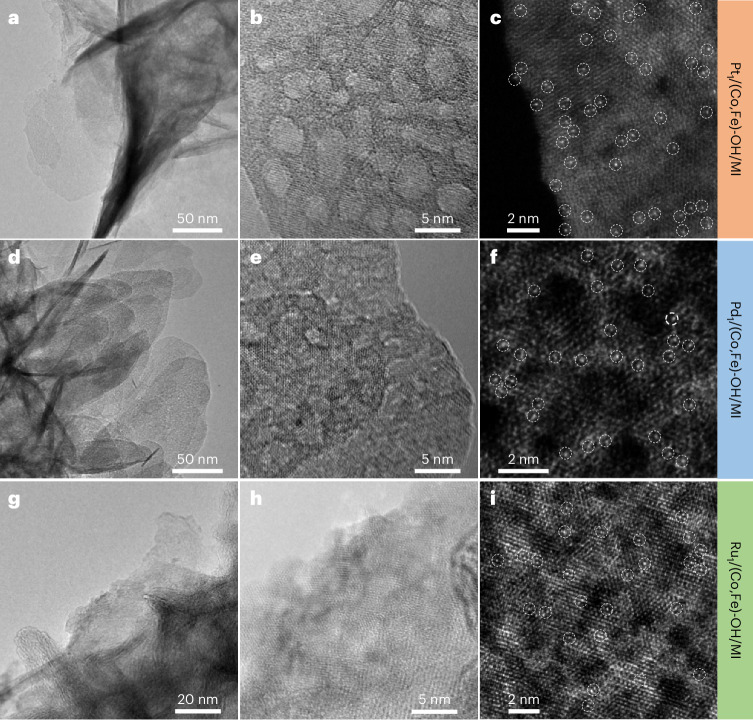
Generalized applicability of the preparation method. **a**–**c**, Pt_1_/(Co,Fe)-OH/MI. **d**–**f**, Pd_1_/(Co,Fe)-OH/MI. **g**–**i**, Ru_1_/(Co,Fe)-OH/MI. TEM images (**a**,**d**,**g**). HR-TEM images (**b**,**e**,**h**). HAADF-STEM images (**c**,**f**,**i**).

Our results show that coordinating MI improves the OER performance of Ir single atoms supported on CoFe hydroxide. We achieved this coordination using a simple, two-step method conducted under mild conditions (Supplementary Scheme 1). Extending our approach, we successfully synthesized Pt_1_/(Co,Fe)-OH/MI (Fig. 6a−c and Supplementary Figs. 50 and 51), Pd_1_/(Co,Fe)-OH/MI (Fig. 6d−f and Supplementary Figs. 52 and 53) and Ru_1_/(Co,Fe)-OH/MI (Fig. 6g−i and Supplementary Figs. 54 and 55), all of which exhibited the desired morphology: atomically dispersed single atoms on porous hydroxide supports (Figs. 1 and 6). This versatile approach opens a new platform of MI-coordinated, noble-metal, single-atom catalysts for exploring a wide range of catalytic reactions.

## Conclusion

This article introduces a straightforward two-step method to prepare Ir single atoms on CoFe hydroxide supports, coordinated with the MI molecule under mild conditions. The resulting catalyst, Ir_1_/(Co,Fe)-OH/MI, features atomically dispersed Ir atoms with enhanced valence, confirmed by HAADF-STEM and XANES analyses. The catalyst showed exceptional OER performance, with an ultralow overpotential of 179 mV at 10 mA cm^−2^ and superior stability even at high current densities. The superior performance compared with commercial IrO_2_ and similar catalysts is attributed to the precise coordination between Ir and MI, which optimizes charge distribution and adjusts *d*-band centres, thereby enhancing the adsorption of OER intermediates. When used in water splitting systems, Ir_1_/(Co,Fe)-OH/MI exhibited low operating voltages and stable long-term performance across various cell configurations. In addition, this preparation method is versatile, can be extended to other noble metals such as Pt, Pd and Ru, and offers a new platform for studying catalytic mechanisms across multiple applications.

## Methods

### Synthesis of Co-MI

First, 2-methylimidazole (20 mmol, MI) was dissolved into deionized water (50 ml) to form a solution, denoted as ‘solution A’. Cobalt nitrate (2.5 mmol, Co(NO_3_)_2_∙6H_2_O) was dissolved in deionized water (50 ml) to form another solution, denoted as ‘solution B’. Then, ‘solution B’ was poured into ‘solution A’ to form ‘solution C’. Next, Ni foam (1 × 2 cm^2^) was put into ‘solution C’. After soaking for 6 h, the Co-MI (that is, Co-MOF) electrode was obtained. Subsequently, the electrode was rinsed with deionized water.

### Synthesis of Ir_1_/(Co,Fe)-OH/MI

Ir_1_/(Co,Fe)-OH/MI was synthesized using a straightforward immersion technique conducted at ambient temperature and pressure. A solution was prepared by dissolving cobalt nitrate (Co(NO_3_)_2_∙6H_2_O, 1 mmol), iron nitrate (Fe(NO_3_)_3_∙6H_2_O, 1 mmol) and an Ir^3+^ aqueous solution (7.14 mg ml^−1^, 350 µl) in a mixture of deionized water (20 ml) and ethylene glycol (10 ml). The pH value of this solution was kept at ~4.5 by adding an appropriate amount of 2 mol l^−1^ NaOH. Subsequently, the pre-prepared Co-MI electrode was immersed in this solution for 20 h. Following immersion, the Ir_1_/(Co,Fe)-OH/MI was obtained and washed three times using deionized water. The mass loading of the Ir_1_/(Co,Fe)-OH/MI catalyst loaded on Ni foam was 0.70 mg cm^−2^. For comparative purposes, Ir_1_/(Co,Fe)-OH, a material without MI, was synthesized. This was achieved through a simple electrodeposition method using an aqueous solution of Co^2+^/Fe^3+^/Ir^3+^. During this process, an electrodeposition potential of −1 V (versus a saturated calomel reference electrode) was maintained, and the electrodeposition time was set at 240 s. In addition, samples without Ir atoms, specifically (Co,Fe)-OH/MI and (Co,Fe)-OH, were synthesized using the above two methods. The active material average mass loadings of the (Co,Fe)-OH/MI, Ir_1_/(Co,Fe)-OH and (Co,Fe)-OH electrodes were recorded as 0.70 mg cm^−2^, 0.71 mg cm^−2^ and 0.73 mg cm^−2^, respectively. The impact of Ni foam thickness, Co/Fe ratio and Ir species’ mass fractions on the OER performance of Ir_1_/(Co,Fe)-OH/MI were investigated for optimization purposes (see Supplementary Figs. 56 and 57).

### Materials characterization

SEM images were obtained using a Thermo Scientific APREO S microscope. TEM, HR-TEM, SAED and elemental mapping images were acquired with a JEOL JEM-F200 transmission electron microscope. AFM images were captured using a DIMENSION ICON CLOSED LOOP SPM. HAADF-STEM images, along with corresponding elemental images, were obtained using a transmission electron microscope (FEI/Thermo Scientific Themis Z) equipped with double spherical aberration correction, operating at 200 kV (the beam current was 44 pA, and the dwell time was 2 μs). The three-dimensional atom-overlapping intensity value representation was obtained by first extracting the grey value of each pixel of the HAADF-STEM image using the Velox software part of the spherical aberration correction STEM software packages, followed by fitting the obtained grey values using intensities ranging from 0 to 100. The two-dimensional atom-overlapping intensity map representation is the top view (or contour plot) of the three-dimensional plot. XRD patterns were recorded using an Ultima IV X-ray powder diffractometer, using Cu K_α_ radiation with a wavelength of 0.154056 nm. In situ Raman spectra were collected on a laser micro confocal Raman spectrometer (inVia, Renishaw). XAS spectra were used to examine the electronic structures and the local atomic environments of the Ir, Co and Fe elements. More details on the XAS technique are provided in Supplementary Information. The atomic ratios of the as-prepared samples were determined using an Agilent 7900 inductively coupled plasma mass spectrometer.

### Electrochemical measurements for OER

The electrochemical measurements of as-prepared catalysts were conducted on an electrochemical workstation (CHI760E) with a three-electrode cell system. The Ir_1_/(Co,Fe)-OH/MI, (Co,Fe)-OH/MI, Ir_1_/(Co,Fe)-OH and (Co,Fe)-OH catalysts grown on Ni foam were used as the working electrodes. A graphite rod was utilized as the counter electrode. The Hg/HgO (1 M KOH, 0.098 V at 25 °C) electrode acted as the reference electrode. For comparison purposes, IrO_2_ and 20% Pt/C inks were dropped on Ni foam to produce the IrO_2_ and 20% Pt/C electrodes (with a mass loading of 0.7 mg cm^−2^), respectively. Before the electrochemical measurements, the working electrodes required electrochemical activation using cyclic voltammetry for 50 cycles at a scan rate of 100 mV s^−1^ in a potential range of 0.2–1.0 V (versus Hg/HgO). The electrochemical activation and working electrode measurements were performed in an oxygen-saturated 1 M KOH aqueous electrolyte at 25 °C. The LSV curves of OER and hydrogen evolution reaction were all measured under a scanning rate of 5 mV s^−1^ with 100% iR compensation. The electrochemical impedance spectroscopy spectra were tested in the frequency range of 0.1 Hz to 100 kHz. Chronoamperometry was performed on Ir_1_/(Co,Fe)-OH/MI at 10 mA cm^−2^, 100 mA cm^−2^, 300 mA cm^−2^ and 600 mA cm^−2^ in a three-electrode cell system. In this paper, the potentials (versus reversible hydrogen electrode, *E*(RHE)) are given using the formula *E*(RHE) = *E*(Hg/HgO) + 0.098 + 0.059 × pH, where pH = 14 in 1 M KOH solution.

Overall water splitting performance was measured in a two-electrode membrane-free cell system and a two-electrode flow cell system at 25 °C. The LSV curves of overall water splitting were measured in an oxygen-saturated 1 M KOH aqueous electrolyte at a scanning rate of 5 mV s^−1^. The chronoamperometry measurements of overall water splitting at 300 mA cm^−2^ and 700 mA cm^−2^ were performed in a two-electrode membrane-free cell system. The chronoamperometry measurement of overall water splitting at 800 mA cm^−2^ was performed in a two-electrode flow cell system.

### First-principle simulations

The specific software, modules and functions used for DFT are detailed in Supplementary Information. The (Co,Fe)-OH models were constructed based on the single-crystal structure of Co(OH)_2_. The primitive cell model of (Co,Fe)-OH underwent full optimization. The 4 × 2 × 1 supercell from the (001) surface was sliced from the bulk (Co,Fe)-OH. To create a model of the Ir single-atom-anchored (Co,Fe)-OH catalyst, an Ir atom was positioned directly on the catalyst surface, coordinating with various metallic and oxygen atoms. The Ir_1_/(Co,Fe)-OH/MI model was constructed by introducing an MI group at the apex of the Ir atom. Throughout the structural optimizations, all atoms were permitted to relax. A vacuum layer of 15 Å was used to prevent periodic interactions in the direction perpendicular to the catalyst’s surface. The convergence criterion for geometry optimization was set with the energy and force converging to 1.0 × 10^−5^ eV per atom and 0.01 eV Å^−1^, respectively. The differential charge density, projected density of states and Gibbs free energy calculations related to the OER of the constructed models are described in the ‘DFT software, modules and functions’ section of Supplementary Information.

## Online content

Any methods, additional references, Nature Portfolio reporting summaries, source data, extended data, supplementary information, acknowledgements, peer review information; details of author contributions and competing interests; and statements of data and code availability are available at 10.1038/s41565-024-01807-x.

## Supplementary information

Supplementary Information The Supplementary Information file includes experimental section, DFT calculations, electrochemically active surface area calculation, turnover frequency calculation, synthesis process for Ir_1_/(Co,Fe)-OH/MI, summary of the research results, Supplementary Scheme 1, Figs. 1−57 and Tables 1−13.

Supplementary Data 1 Source data for the Supplementary figures.

## Source data

Source Data Fig. 1 Statistical source data.

Source Data Fig. 2 Statistical source data.

Source Data Fig. 3 Statistical source data.

Source Data Fig. 4 Statistical source data.

Source Data Fig. 5 Statistical source data.

## Data Availability

The data that support the main findings are available in the main text and Supplementary Information. Source data are provided with this paper.
