# Supplementary material for: Out-of-plane coordination of iridium single atoms with organic molecules and cobalt–iron hydroxides to boost oxygen evolution reaction
Source: Nat Nanotechnol. 2024 Oct 21;20(1):57–66. doi: 10.1038/s41565-024-01807-x (PMC11750697; doi:10.1038/s41565-024-01807-x)
Supplement: Supplementary file 1 — The Supplementary Information file includes experimental section, DFT calculations, electrochemically active surface area calculation, turnover frequency calculation, synthesis process for Ir1/(Co,Fe)-OH/MI, summary of the research results, Supplementary Scheme 1, Figs. 1−57 and Tables 1−13. [file 41565_2024_1807_MOESM1_ESM.pdf]

nature nanotechnology

Supplementary information https://doi.org/10.1038/s41565-024-01807-x

Out-of-plane coordination of iridium single atoms with organic molecules and cobalt–iron hydroxides to boost oxygen evolution reaction

In the format provided by the authors and unedited

Table of Contents

Experimental section………2
DFT software, modules and functions……………………………………………………………………………………………3
Electrochemical active surface area calculation…………………………………………………………………………………5
Turnover frequency calculation……………………………………………………………………………………………………6
Synthesis process for Ir_1/(Co,Fe)-OH/MI……………………………………………………………………………………7
Summary of the research results…………………………………………………………………………………………………8
Supplementary Scheme 1……9
Supplementary Figs. 1−57………………………………………………………………………………………………10−70
Supplementary Tables 1−13……………………………………………………………………………………………71−83
References……84

Experimental section

Materials

Cobalt nitrate hexahydrate ($Co(NO_3)_2·6H_2O$, solid, AR), ferric nitrate hexahydrate ($Fe(NO_3)_3·6H_2O$, solid, AR), methylimidazole ($C_4H_6N_2$, MI, solid, AR), KOH (99.999% metals basis, solid, electron-grade), NaOH (99.99% trace metals basis, solid) were purchased from Aladdin. Commercial Pt/C (20 wt% Pt, solid), iridium trichloride ($IrCl_3$, 99.9%), and iridium oxide (IrO_2, solid, 99.9%) were purchased from Sigma-Aldrich LLC.

X-ray absorption spectroscopy (XAS) analysis

Ir L_3-edge analysis was performed at the BL13SSW beamline of the Shanghai Synchrotron Radiation Facility (SSRF, Shanghai, China). Si (111) crystal monochromators were used. The XAS spectra were recorded simultaneously using two modes at room temperature: the transmission mode in a gas ionization chamber and the fluorescence mode in a Lytle ionization chamber. The XAS spectra of Ir_1/(Co,Fe)-OH/MI and Ir_1/(Co,Fe)-OH) were adopted in fluorescence mode, while the spectra of Ir foil and IrO_2 were in transmission mode. The Co and Fe *K*-edge XAS spectra were performed at the Taiwan Synchrotron Radiation Facility. The spectra were analyzed using the Athena, Artemis, and Hama packages.

DFT software, modules and functions

To understand electronic structures, as well as the oxygen evolution reaction (OER) mechanism of iridium (Ir) single-atom sites on CoFe-LDH, with and without the MI organic molecule, we performed density functional theory (DFT) calculations using the Vienna *ab initio* simulation package (VASP)[1–4]. We used the projected augmented wave (PAW) potential to describe electron-ion interactions and the generalized gradient approximation (GGA) with the spin-polarized Perdew-Burke-Ernzerhof (PBE) functional to model exchange-correlation energy[5–7]. A 400 eV energy cutoff was used for the plane-wave expansion. For the surface calculations, the first Brillouin zone was sampled using a 3×3×1 Monkhorst-Pack grid[8]. To compute the catalysts' density of states (DOS), we employed a denser 5×5×1 k-point mesh. Additionally, the DFT-D3 method was used to account for van der Waals interactions between the adsorbed intermediates and the catalysts[9].

We used the computational hydrogen electrode (CHE) model developed by Nørskov and coworkers to evaluate OER activity at different sites[10,11]. This model relies on several key assumptions. First, it assumes that the chemical potential of ($H^+ + e^-$) is equivalent to that of ½ H_2, utilizing the standard hydrogen electrode as the reference potential. Second, it models the stability of adsorbed oxygenate intermediates with solvation effects, employing an implicit solvation model. Third, it calculates the impact of potentail bias on electronic states by an –eU (U is the applied electrode potential) energy shift. Finally, the model incorporates the impact of the pH by correcting the free energy of H^+ ions according to

$$G(\mathrm{pH}) = -k\mathrm{T}\ln[\mathrm{H}^+] = k\mathrm{T}\ln 10 \quad \times \mathrm{pH} \tag{1}$$

With these assumptions, the adsorption free energies of oxygenate intermediates are calculated by incorporating the zero-point energy (ZPE) and entropy (S) corrections as follows:[12,13]

$$\Delta \mathrm{G_{ads}} = \Delta \mathrm{E_{ads}} + \Delta \mathrm{ZPE} - \mathrm{T}\Delta \mathrm{S} \tag{2}$$

The ΔZPE (change in zero-point energy) is calculated from the vibrational frequencies of the adsorbed species. The adsorption energy (ΔE_{ads}) are derived using the following equation[11]:

$$\Delta \mathrm{E_{O*}} = \mathrm{E_{O*}} - \mathrm{E_*} - [\mathrm{E_{H_2O}} - \mathrm{E_{H_2}}] \tag{3a}$$

$$\Delta \mathrm{E_{OH*}} = \mathrm{E_{OH*}} - \mathrm{E_*} - [\mathrm{E_{H_2O}} - 1/2\,\mathrm{E_{H_2}}] \tag{3b}$$

$$\Delta \mathrm{E_{OOH*}} = \mathrm{E_{OOH*}} - \mathrm{E_*} - [2\mathrm{E_{H_2O}} - 3/2\,\mathrm{E_{H_2}}] \tag{3c}$$

where E_*, E_{OOH*}, E_{OH*}, and E_{O*} are the energies of catalyst substrate without and with absorbed OOH, OH and O, respectively. E_{H_2O} and E_{H_2} are total energies of H_2O and H_2 in the gas, respectively. The * symbol indicates absorbed species.

In alkaline media, the overall OER can be expressed as[10]:

$$4\mathrm{OH^-} \rightarrow \mathrm{O_2(g)} + 2\mathrm{H_2O(l)} + 4\mathrm{e^-} \tag{4}$$

The elementary reaction steps along the concerted four-electron process are given by[10,14]:

(i) $OH^- \rightarrow OH^* + e^-$ (5a)

(ii) $OH^* + OH^- \rightarrow O^* + H_2O(l) + e^-$ (5b)

(iii) $O^* + OH^- \rightarrow OOH^* + e^-$ (5c)

(iv) $OOH^* + OH^- \rightarrow O_2(g) + H_2O(l) + e^-$ (5d)

where (l) and (g) refer to liquid and gas phases, respectively.

The change in Gibbs free energy (△G) of each reaction step is given by[10]

$$\Delta G = \Delta E + \Delta ZPE - T\Delta S + \Delta G_U + G(pH) \quad (6)$$

where △E is reaction energy difference, $\triangle G_U$ is –eU (U is the applied electrode potential), and T is temperature. The free energy change of O_2 is 4.92 eV obtained from $2H_2O \rightarrow O_2 + 2H_2$[10]. The reliability of this methodology is supported by numerous successful research works[15–18].

For example, under an electrode potential U=0 V, the △G for each step can be calculated by the following equations:

$$\Delta G_i = G(OH^*) + G(e^-) - G(OH^-) - G(^*)$$
$$= \{\Delta G_{OH*} + G(^*) + [G(H_2O) - 1/2G(H_2)]\} + 1/2G(H_2) - G(H_2O) - G(^*) = \Delta G_{OH*} \quad (7a)$$

$$\Delta G_{ii} = G(O^*) + G(H_2O) + G(e^-) - G(OH^*) - G(OH^-)$$
$$= \{\Delta G_{O*} + G(^*) + [G(H_2O) - G(H_2)]\} + 1/2G(H_2) - \{\Delta G_{OH*} + G(^*) + [G(H_2O) - 1/2G(H_2)]\} = \Delta G_{O*} - \Delta G_{OH*} \quad (7b)$$

$$\Delta G_{iii} = G(OOH^*) + G(e^-) - G(O^*) - G(OH^-)$$
$$= \{\Delta G_{OOH*} + G(^*) + [2G(H_2O) - 3/2G(H_2)]\} + 1/2G(H_2) - \{\Delta G_{O*} + G(^*) + [G(H_2O) - G(H_2)]\} - G(H_2O) = \Delta G_{OOH*} - \Delta G_{O*} \quad (7c)$$

$$\Delta G_{iv} = G(O_2) + G(e^-) + G(H_2O) - G(OOH^*) - G(OH^*) - G(OH^-)$$
$$= \{4.92 + 2G(H_2O) - 2G(H_2)\} + 1/2G(H_2) - \{\Delta G_{OOH*} + G(^*) + [2G(H_2O) - 3/2G(H_2)]\} = 4.92 - \Delta G_{OOH*} \quad (7d)$$

Then, the overpotential is obtained the following formula[10]:

$$\eta = \max(\Delta G_i, \Delta G_{ii}, \Delta G_{iii}, \Delta G_{iv})/e - 1.23 \quad (8)$$

Accordingly, the energy barriers and overpotentials of the concerted four-electron pathway are obtained at various electrode potentials.

Electrochemical active surface area calculation

The electrochemically active surface area (ECSA) was estimated from the double-layer capacitance (C_{dl}). To derive the C_{dl} value, cyclic voltammetry (CV) curves were recorded in the potential range of 0.3~0.4 V (versus Hg/HgO), at the scan rates of 20, 40, 60, 80, and 100 mV s^{-1}. C_{dl} was calculated from the linear slope of ΔJ/2 versus scan rate, where ΔJ represents the difference in current density (ΔJ = positive current density – negative current density) at 0.35 V (vs. Hg/HgO).

Ni foam served as the current collector, and its specific capacitance (2.68 mF cm^{-2}) was used for subsequent ECSA calculations[19]. The ECSA is thus given by:

$$A_{ECSA} = C_{dl}/(2.68\ \mathrm{mF\ cm^{-2}\ per\ ECSA\ cm^2}) \quad (9)$$

Turnover frequency calculation

The turnover frequency (TOF) values were calculated using the following equation[14,19]:

$$\mathrm{TOF} = \frac{|j|A}{\delta \cdot F \cdot n} \tag{10}$$

Here, $|j|$ is the absolute value of the current density (A cm^{-2}) measured at an overpotential of 250 mV (equivalent to 1.48 V vs. RHE) by linear sweep voltammetry (LSV) in a 1 M KOH aqueous electrolyte, A is the electrode plane area (0.5 cm^2), F is the Faraday constant (96485 C mol^{-1}), n is the number of electrons consumed to form one O_2 molecule from water (that is, 4 electrons for OER), and δ indicates the molar number of metal active sites obtained from ICP-MS characterization. Based on the ICP−MS results, the δ values were calculated using the following equation[19]:

$$\delta = \frac{\mathrm{m_{catalyst}} \times \mathrm{W_{Co}}}{\mathrm{M_{Co}}} + \frac{\mathrm{m_{catalyst}} \times \mathrm{W_{Fe}}}{\mathrm{M_{Fe}}} + \frac{\mathrm{m_{catalyst}} \times \mathrm{W_{Ir}}}{\mathrm{M_{Ir}}} \tag{11}$$

Here, $\mathrm{m_{catalyst}}$ indicates the mass of the catalyst on a Ni foam electrode of cross-sectional area 0.5 cm^2 (the masses of Ir_1/(Co,Fe)-OH/MI, (Co,Fe)-OH/MI, and IrO_2 were all 0.35 mg; the mass of Ir_1/(Co,Fe)-OH is 0.355 mg, and the mass of (Co,Fe)-OH is 0.365 mg). M_{Co}, M_{Fe}, and M_{Ir} are the relative atomic masses of Co, Fe, and Ir atoms, respectively. The symbols W_{Co}, W_{Fe}, and W_{Ir} indicate the weight percentage of Co, Fe, and Ir elements, respectively, as determined from ICP−MS.

Synthesis process for Ir_1/(Co,Fe)-OH/MI

Ir_1/(Co,Fe)-OH/MI was prepared using a process that converted the Co-based complex (Co-MI) into CoFe hydroxide. As illustrated in Supplementary Scheme 1, this synthesis procedure required two steps. The synthesis of the Ir_1/(Co,Fe)-OH/MI sample started with the growth of Co-MI on nickel foam using an aqueous solution containing Co^{2+} ions and MI molecules. The resulting Co-MI exhibited petal-shaped sheets characterized by nanometer-sized pores (Supplementary Fig. 1). For the second step, Co-MI was converted into Ir_1/(Co,Fe)-OH/MI by immersion in an ethylene glycol/water solution containing Co^{2+}, Fe^{3+}, and Ir^{3+} (Supplementary Figs. 2−5). This conversion involved two reactions: 1) Co-MI was progressively dissolved into Co^{2+} ions and MI molecules in the acidic environment (pH of ~4.5) (Supplementary Fig. 2), resulting in lots of Co^{2+} ions and MI molecules entering the ethylene glycol/water solution; 2) following their dissolution, Co^{2+}/Fe^{3+}/Ir^{3+} ions and MI molecules transformed into Ir_1/(Co,Fe)-OH/MI by the Fe^{3+} flocculation at a pH of ~4.5 (Supplementary Figs. 3−5 and Supplementary Table 1). After 20 h, the Ir_1/(Co,Fe)-OH/MI catalyst was obtained. The as-prepared Ir_1/(Co,Fe)-OH/MI compound was porous (Supplementary Fig. 6), presenting numerous channels for the transport of OER reactants (*e.g.*, OH^-, H_2O, and O_2). Notably, this two-step synthetic method was completed at room temperature and atmospheric pressure without needing energy-demanding reaction conditions (*e.g.*, high temperature and high pressure). Additionally, this method was suitable for preparing large-size (measuring tens of square centimeters) Ir_1/(Co,Fe)-OH/MI electrodes (Supplementary Fig. 7), laying a foundation for applications at scale.

Summary of the research results

This article presents a facile two-step method for preparing Ir single atoms on CoFe hydroxide supports coordinated with the organic molecule MI. This approach, conducted under mild conditions (room temperature, atmospheric pressure), involves the growth of Co-MI and its subsequent transformation into CoFe-based hydroxides. The resulting catalyst, Ir_1/(Co,Fe)-OH/MI, exhibits atomically dispersed Ir single atoms on porous CoFe hydroxide supports, as confirmed by high angle annular dark field-scanning transmission electron microscopy (HAADF-STEM). The X-ray absorption near edge structure (XANES) analysis further verifies the out-of-plane coordination between Ir single atoms and MI molecules in Ir_1/(Co,Fe)-OH/MI, along with its increased valence. When used for OER electrocatalysts, Ir_1/(Co,Fe)-OH/MI demonstrated an ultralow overpotential of 179 mV at a current density of 10 mA cm^{-2} and small Tafel slope of 24 mV dec^{-1}. Ir_1/(Co,Fe)-OH/MI has an overpotential of only 257 mV even at a high current density of 600 mA cm^{-2}, outperforming both Ir_1/(Co,Fe)-OH (without MI) and commercial IrO_2. In addition, Ir_1/(Co,Fe)-OH/MI has high areal/mass activities, approximately 15.5/9.8 and 16.4/58.3 times those of Ir_1/(Co,Fe)-OH and commercial IrO_2, respectively. The remarkable OER performance can be attributed to the coordination between Ir single atoms and MI molecules. First-principle simulations indicate that this coordination reduces charge density near the Ir site, leading to a positive shift in the *d*-band centers of both the Ir and adjacent Co sites. This shift promotes stable adsorption of OER intermediates, enhancing catalytic activity. When employed as overall water-splitting electrodes at a current density of 10 mA cm^{-2}, the Ir_1/(Co,Fe)-OH/MI electrode-based membrane-free electrolytic cells exhibit small water-splitting voltages of 1.44 V for the asymmetric Ir_1/(Co,Fe)-OH/MI || 20% Pt/C cell and 1.51 V for the symmetric Ir_1/(Co,Fe)-OH/MI || Ir_1/(Co,Fe)-OH/MI cell. At a current density of 800 mA cm^{-2}, the water-splitting voltage of the Ir_1/(Co,Fe)-OH/MI || Ir_1/(Co,Fe)-OH/MI cell was only 1.79 V. The corresponding membrane-free electrolytic cells can operate stably for 120 h without a noticeable decline in performance at 300 and 700 mA cm^{-2}. In a two-electrode flow cell system, the Ir_1/(Co,Fe)-OH/MI || Ir_1/(Co,Fe)-OH/MI cell can run stably for 100 h at 800 mA cm^{-2}. The AEM water electrolyzer, utilizing Ir_1/(Co,Fe)-OH/MI for both electrodes, outperformed an analogous IrO_2 || 20% Pt/C electrolyzer built using commercial materials. Furthermore, the AEM Ir_1/(Co,Fe)-OH/MI-based electrolyzer operated stably over 150 hours at 500 mA cm^{-2}. Importantly, this simple method of preparing Ir single atoms coordinated with MI organic molecules on hydroxides can be extended to other noble-metal systems, including Pt, Pd, and Ru. This study provides a new material platform for investigating catalytic mechanisms in other applications.

Supplementary Scheme

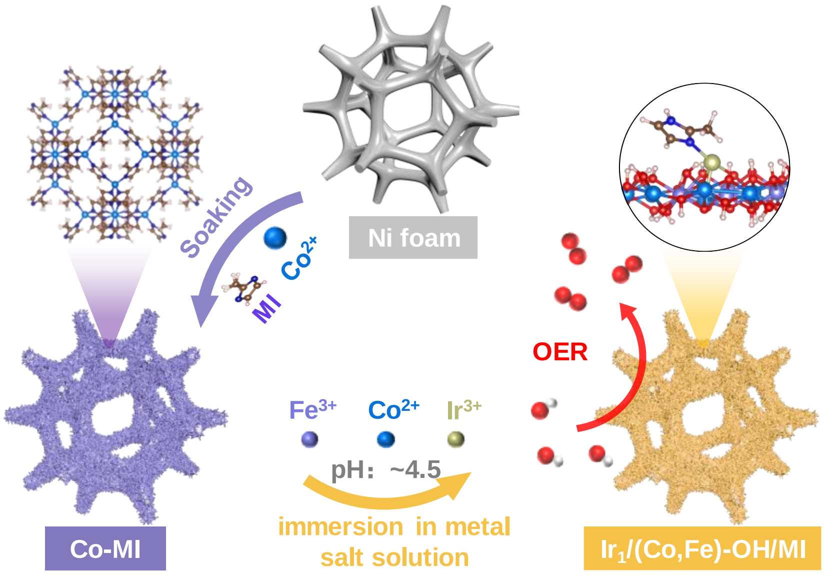

Supplementary Scheme 1 Schematic drawing illustrating how the Ir_1/(Co,Fe)-OH/MI catalyst was synthesized.

The synthesis procedure involved two main steps:

i) A Co-based MOF (Co-MI) was grown on Ni foam in a Co^{2+}/MI solution after sitting for 6 hours (see "The Synthesis of Co-MI" in the Methods section of the main text).

ii) Single Ir atoms coordinated with organic molecules (MI) were introduced into CoFe hydroxides, forming Ir_1/(Co,Fe)-OH/MI. This was achieved by immersing Co-MI in a Co^{2+}/Fe^{3+}/Ir^{3+} salt solution (pH ≈ 4.5) for 20 hours, inducing the transformation to Ir_1/(Co,Fe)-OH/MI (see "Synthesis of Ir_1/(Co,Fe)-OH/MI" in the Methods section of the main text).

Supplementary Figures

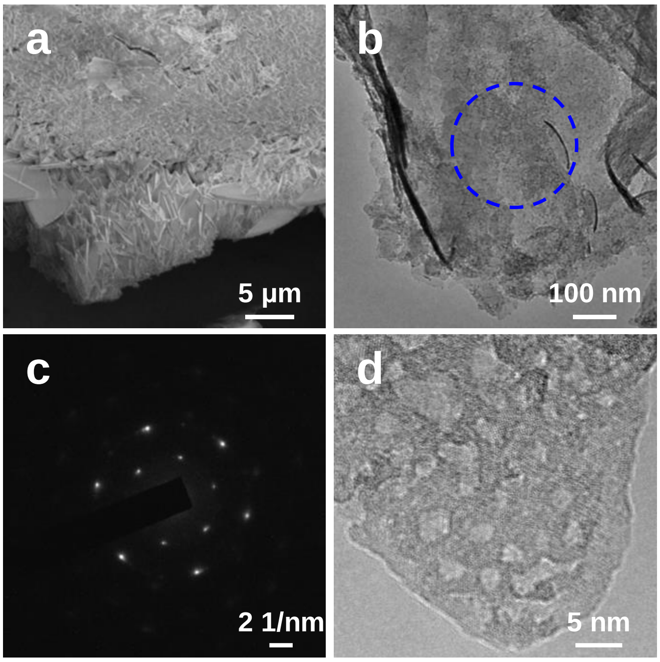

Supplementary Fig. 1 SEM and TEM images of Co-MI. a SEM image. **b** TEM image. **c** SAED image. **d** HR-TEM micrograph.

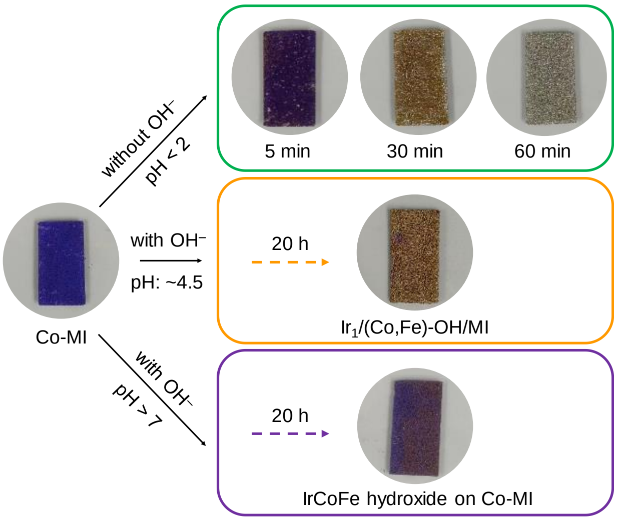

Supplementary Fig. 2 Digital photographs of samples at different immersion times during the conversion of Co-MI into CoFe-based hydroxide.

Controlled experiments revealed a strong pH dependence for the growth of Ir_1/(Co,Fe)-OH/MI:

pH < 2: The Co-MI framework dissolves rapidly (within 5 minutes) into MI and Co^{2+} ions. Released MI molecules increase pH, triggering Fe^{3+} precipitation to form Ir_1/(Co,Fe)-OH/MI. However, the complete dissolution of Co-MI before Fe^{3+} precipitation results in a weakly structured Ir_1/(Co,Fe)-OH/MI with poor adhesion to the Ni foam substrate (as seen in the 60-minute case).

pH ≈ 4.5: Co-MI dissolution and Fe^{3+} hydrolysis occur simultaneously, allowing Ir_1/(Co,Fe)-OH/MI to grow gradually along the Co-MI framework. This process, with prolonged soaking (over 20 hours), yields Ir_1/(Co,Fe)-OH/MI with excellent nanosheet morphology and strong substrate adhesion.

pH > 7: Co-MI remains undissolved, and Fe^{3+} precipitation is rapid. This leads to the formation of IrCoFe hydroxides on the surface of Co-MI, rather than the desired structure.

Further details on the growth process are given in Supplementary Figs. 3−5 and Supplementary Table 1.

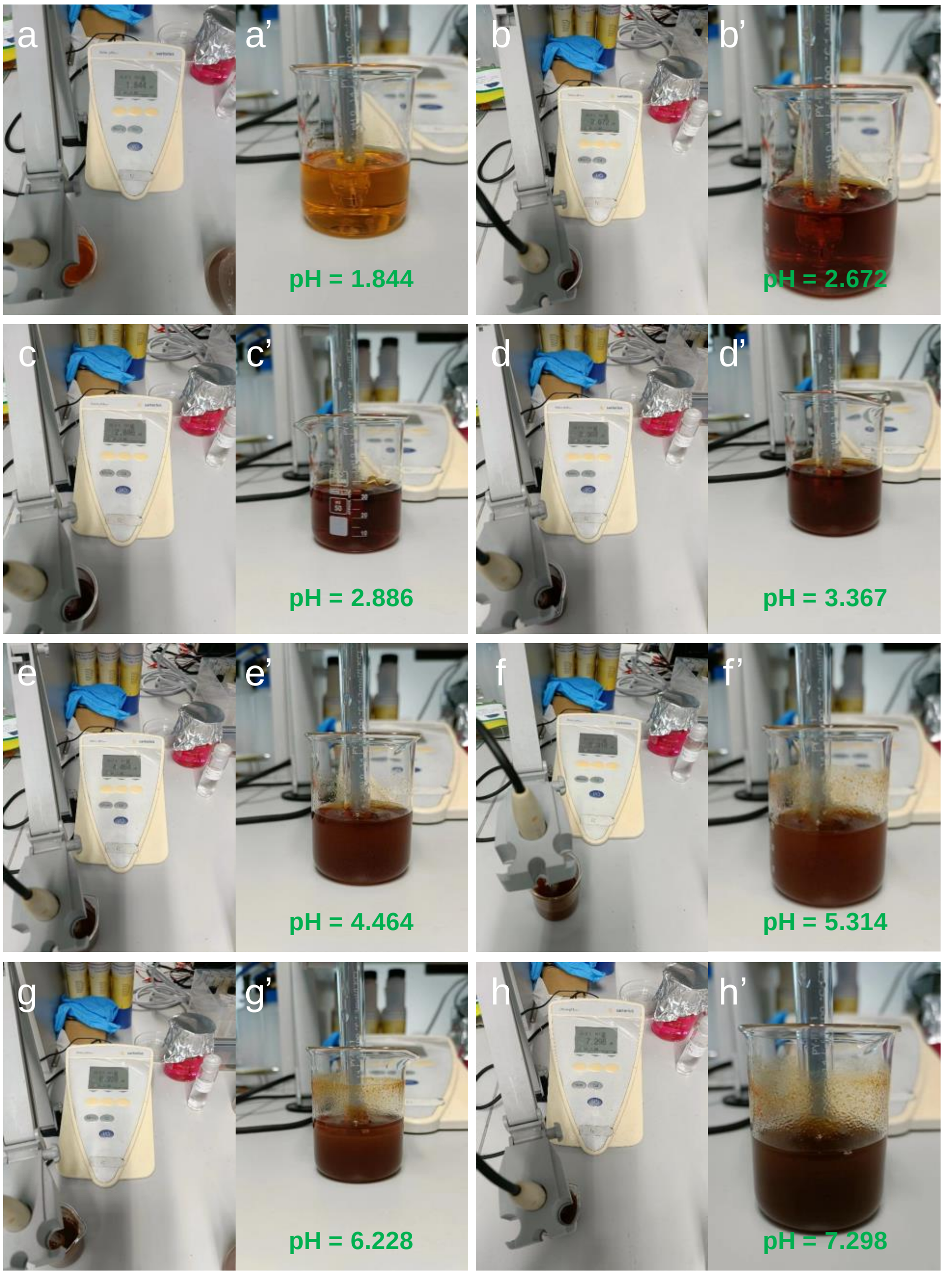

Supplementary Fig. 3 Digital photographs of immersion solutions under different initial pH values. Note: the room temperature was 20 °C. **a,a'** pH = 1.844. **b,b'** pH = 2.672. **c,c'** pH = 2.886. **d,d'** pH = 3.367. **e,e'** pH = 4.464. **f,f'** pH = 5.314. **g,g'** pH = 6.228. **h,h'** pH = 7.298.

As the initial pH value of the $Co^{2+}/Fe^{3+}/Ir^{3+}$ solution increases, Fe^{3+} ions rapidly precipitate to form hydroxide/oxyhydroxide compounds. This precipitation begins when the pH exceeds 3.4 and becomes particularly noticeable when the pH surpasses 4.5. For further exploration of how sample growth is affected at different pH values, please refer to Supplementary Fig. 4.

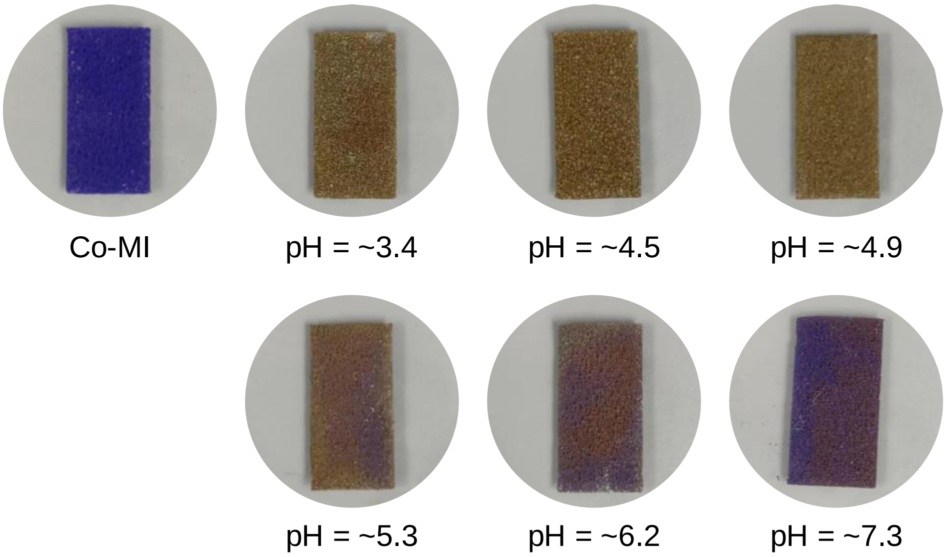

Supplementary Fig. 4 The digital photographs of samples after immersion for 20 h in $Co^{2+}/Fe^{3+}/Ir^{3+}$ solutions with different initial pH values.

The initial pH value of the immersion solution significantly impacts the growth of Ir_1/(Co,Fe)-OH/MI after a 20-hour immersion. At pH ~3.4, Ir_1/(Co,Fe)-OH/MI distribution is uneven, leaving areas of bare Ni foam. Optimal growth occurs between pH 4~5, resulting in uniform Ir_1/(Co,Fe)-OH/MI coverage. Above pH 5, blue Co-MI residues remain, indicating incomplete conversion to CoFe hydroxide/oxyhydroxide due to limited Co-MI dissolution at higher pH. The amount of residue increases with pH. At pH ~7.3, minimal Co-MI dissolves, and IrCoFe hydroxides form a coating, suggesting limited conversion of Co-MI to Ir_1/(Co,Fe)-OH/MI. See Supplementary Table 1 for controlled experiments examining the role of each component in the $Co^{2+}/Fe^{3+}/Ir^{3+}$ immersion solution.

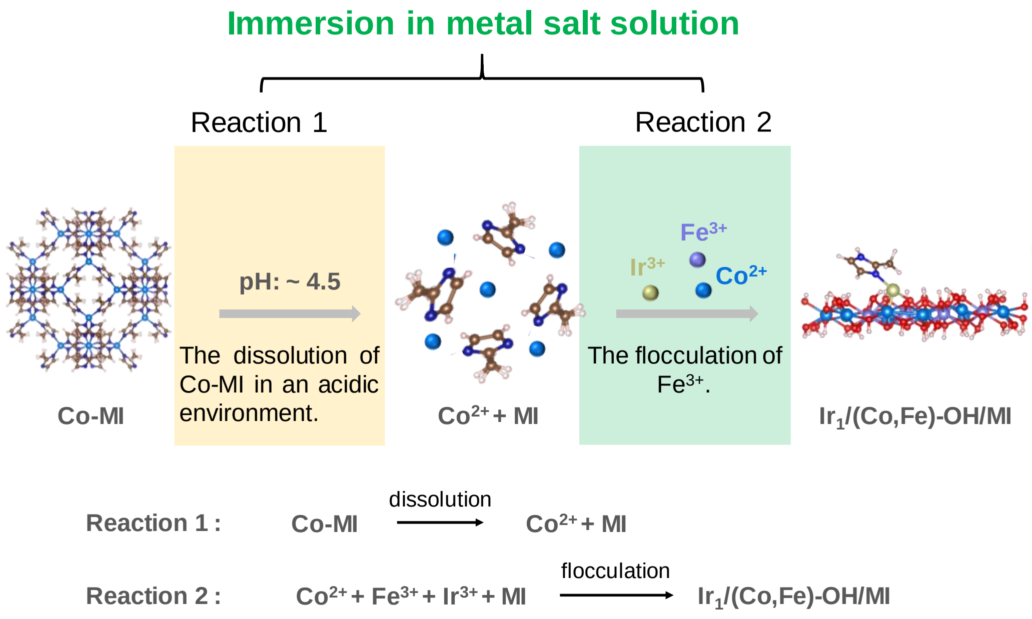

Supplementary Fig. 5 Diagram of the process converting Co-MI into Ir_1/(Co,Fe)-OH/MI.

Based on the results in Supplementary Figs. 2–4 and Table 1, the immersion process is driven by two key reactions: the dissolution of Co-MI and the precipitation of Fe^{3+}. This combination transforms Co-MI into CoFe hydroxide, forming Ir_1/(Co,Fe)-OH/MI.

Crucially, this preparation method offers versatility. By modifying the MOF type or the metal ions in the solution, a wide range of novel metal hydroxides (such as NiFe, ZnFe, ZnCoFe, NiCoFe) can be synthesized. This capability provides a platform for developing a diverse set of high-performance electrocatalysts.

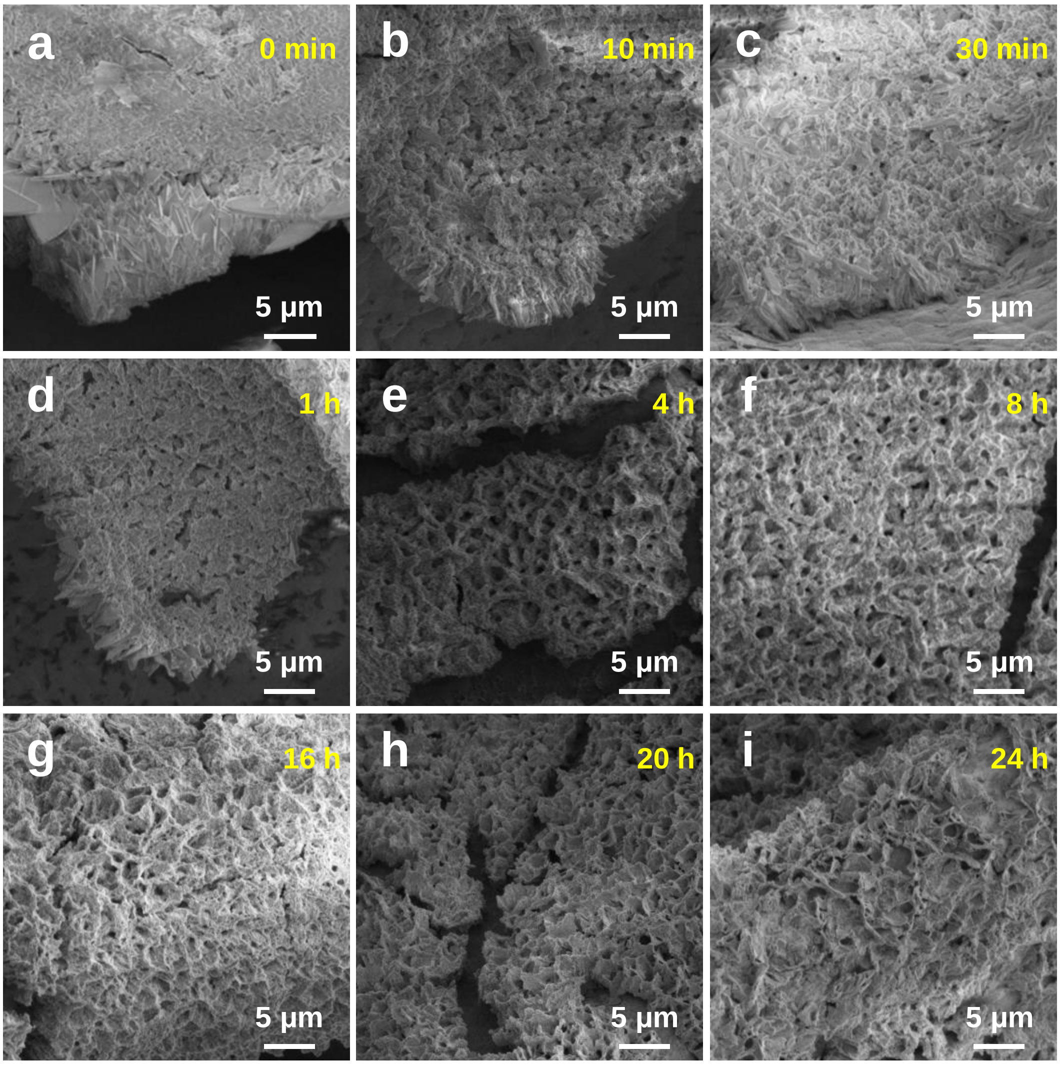

Supplementary Fig. 6 SEM images of as-prepared samples converted from Co-MI under different soaking times in an ethylene glycol/water solution containing Co^{2+}, Fe^{3+}, and Ir^{3+} ions. a 0 min. **b** 10 min. **c** 30 min. **d** 1 h. **e** 4 h. **f** 8 h. **g** 16 h. **h** 20 h. **i** 24 h.

The as-prepared Ir_1/(Co,Fe)-OH/MI sample exhibits a nanosheet morphology after soaking for over 20 hours.

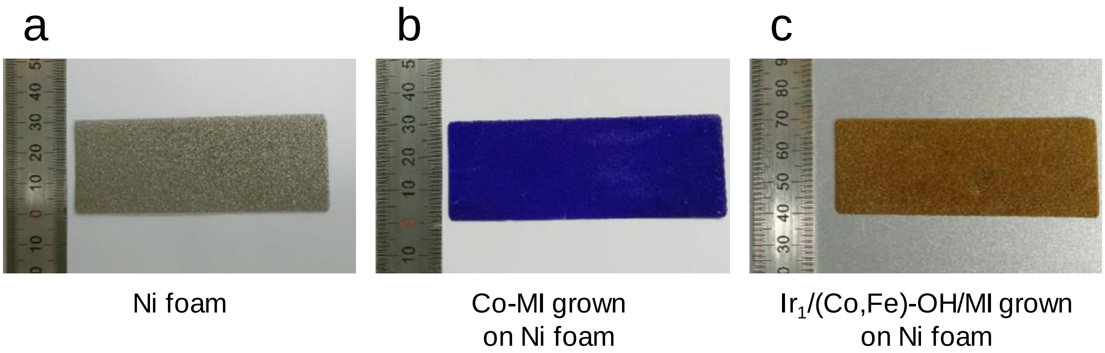

Supplementary Fig. 7 Photographs of large-size samples. **a** Ni foam. **b** Co-MI. **c** Ir_1/(Co,Fe)-OH/MI.

We prepared large-size Co-MI and Ir_1/(Co,Fe)-OH/MI samples using the methods described in this article (see "Methods" of main text).

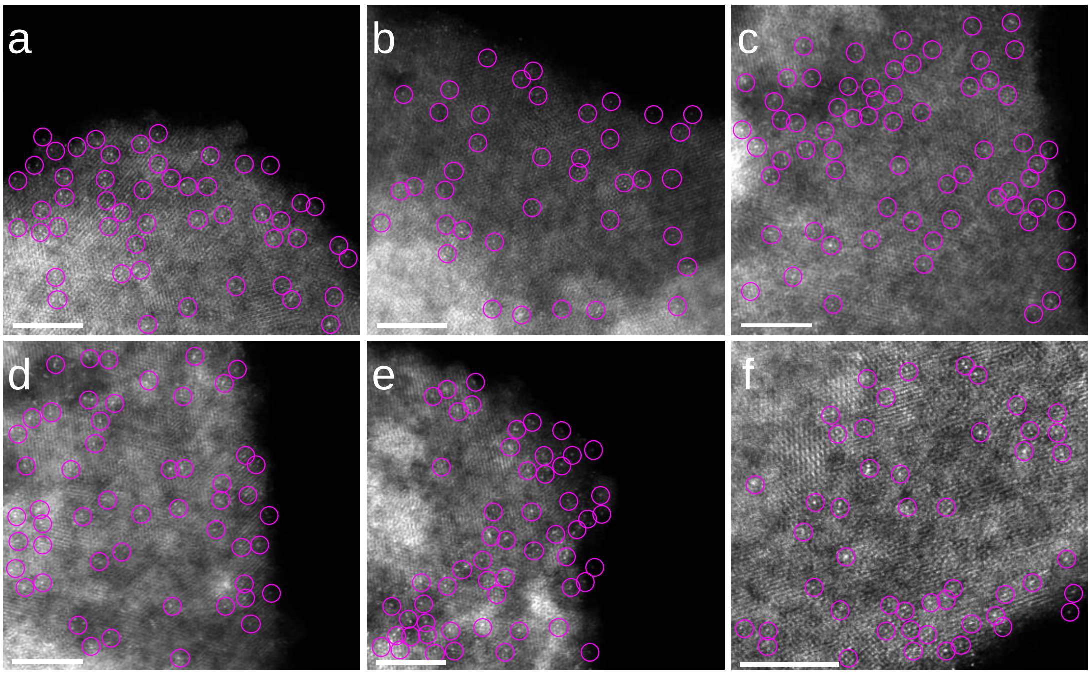

Supplementary Fig. 8 HAADF-STEM images of the Ir_1/(Co,Fe)-OH/MI sample. a-f The HAADF-STEM images at different positions of the Ir_1/(Co,Fe)-OH/MI sample. The scale bar is 5 nm.

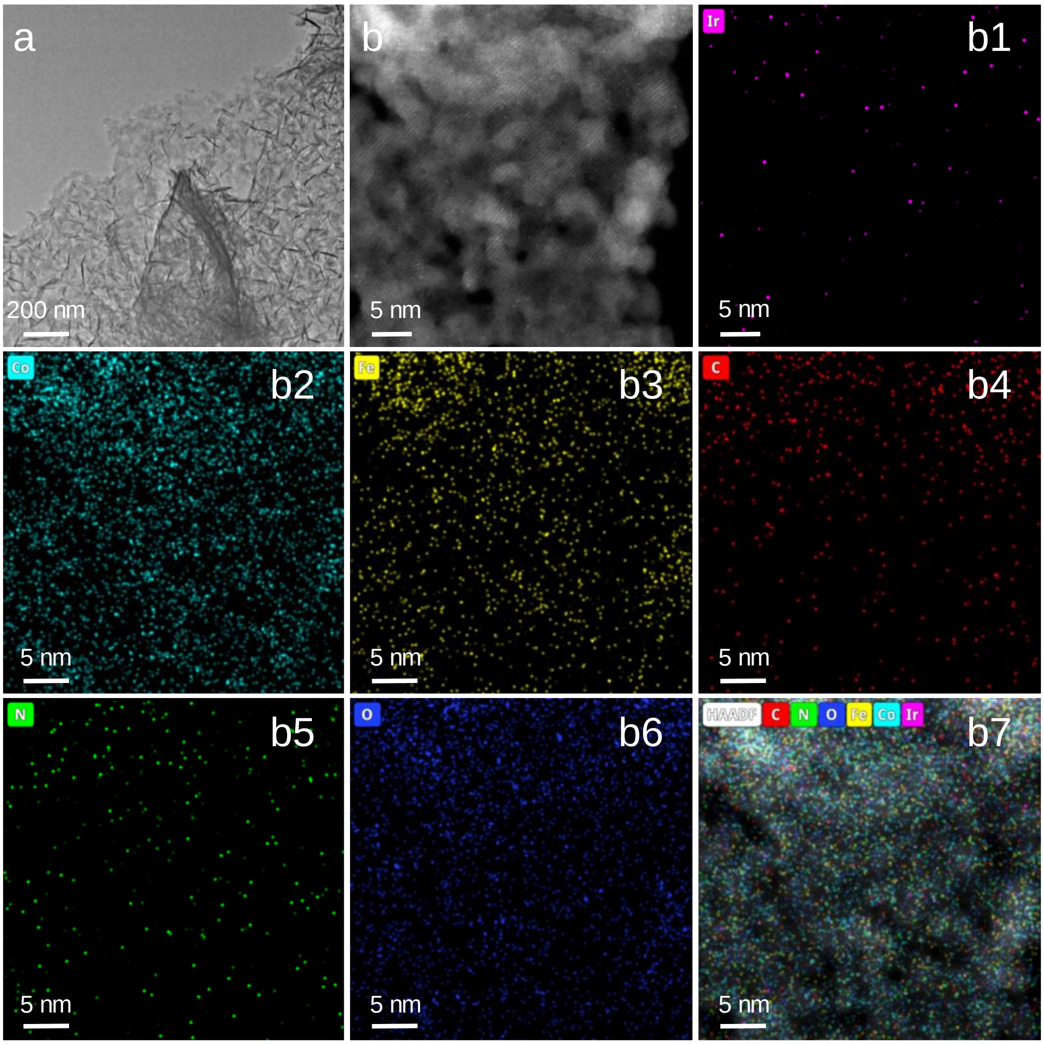

Supplementary Fig. 9 TEM image and the corresponding element mapping images of Ir_1/(Co,Fe)-OH/MI sample. a TEM image. **b** HAADF-TEM micrograph. **b1-b7** Corresponding element maps.

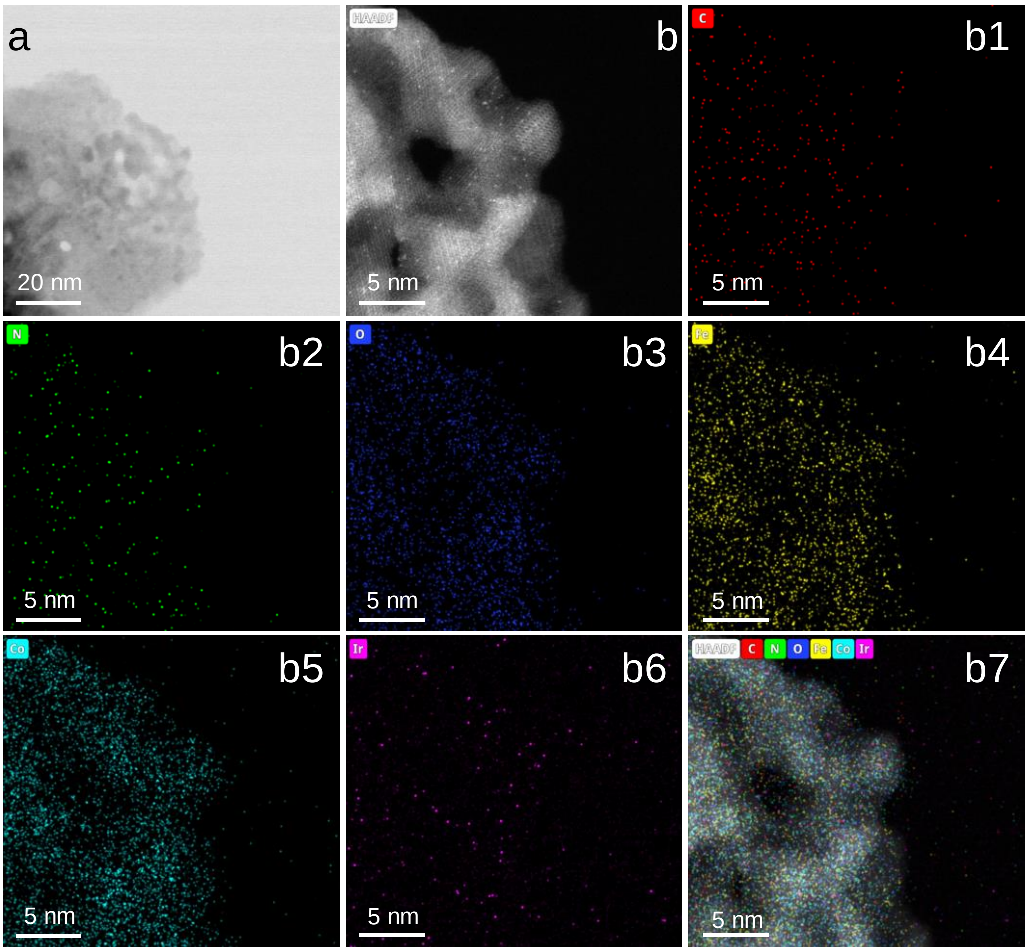

Supplementary Fig. 10 TEM image and the corresponding element mapping images of Ir_1/(Co,Fe)-OH/MI sample. a TEM image. **b** HAADF-STEM image. **b1-b7** Corresponding element maps.

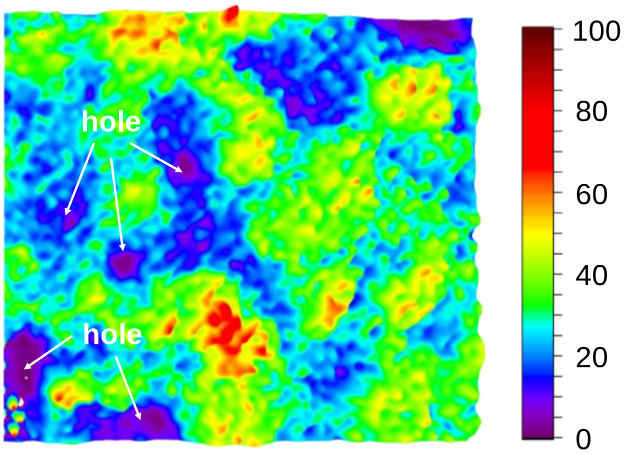

Supplementary Fig. 11 2D atom-overlapping intensity value image of Ir_1/(Co,Fe)-OH/MI sample.

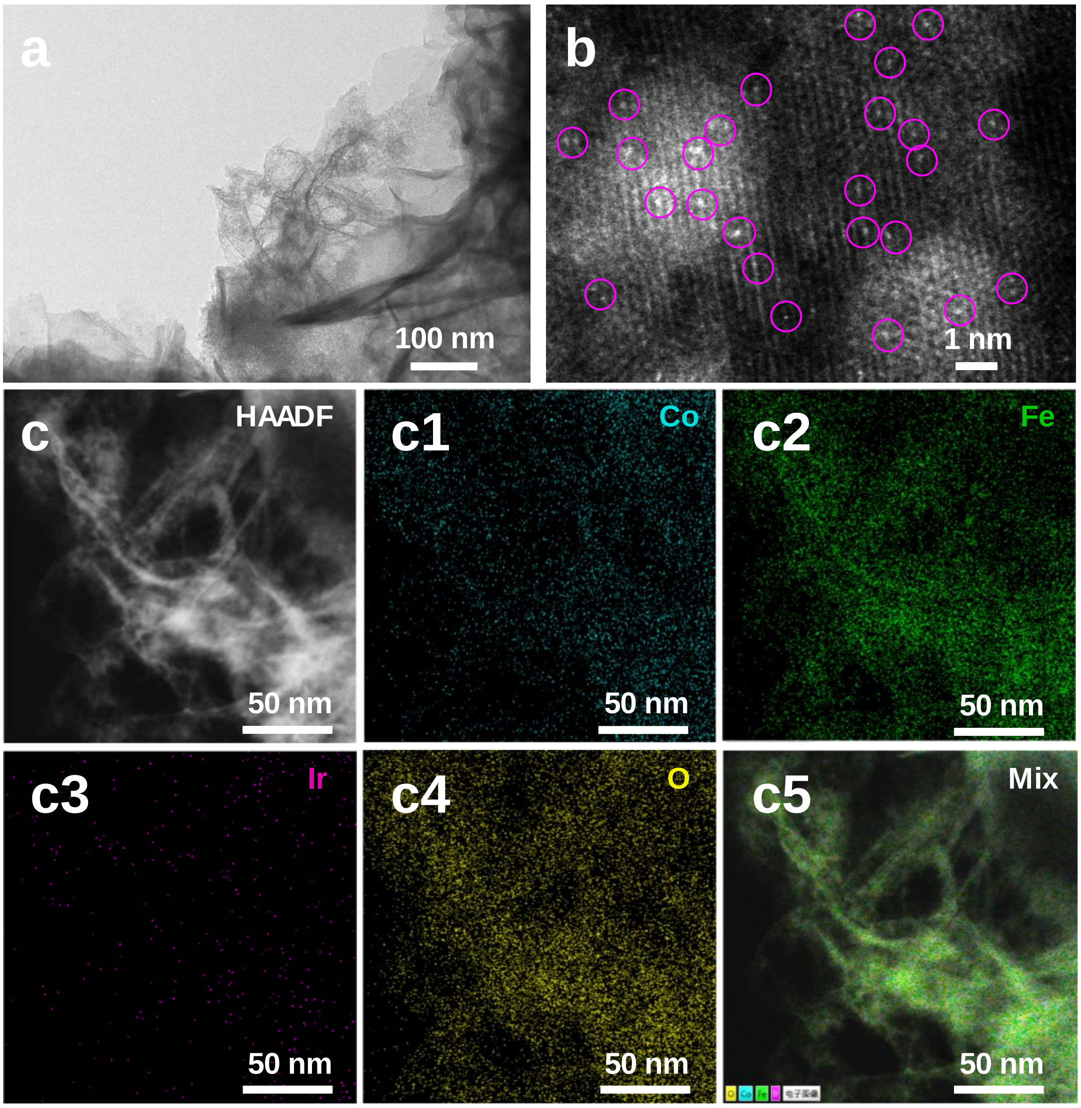

Supplementary Fig. 12 TEM images and the corresponding element mapping images of Ir_1/(Co,Fe)-OH sample. **a** TEM image. **b** HAADF-STEM image. **c** HAADF image. **c1-c5** The corresponding element mapping images.

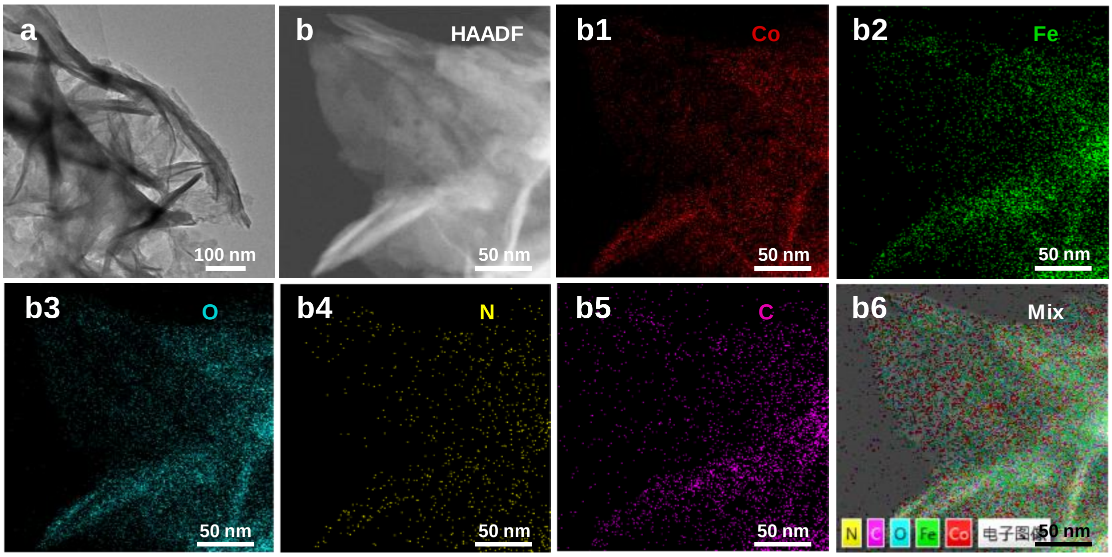

Supplementary Fig. 13 TEM images and the corresponding element mapping images of (Co,Fe)-OH/MI sample. a TEM image. **b** HAADF image. **b1-b6** The corresponding element mapping images.

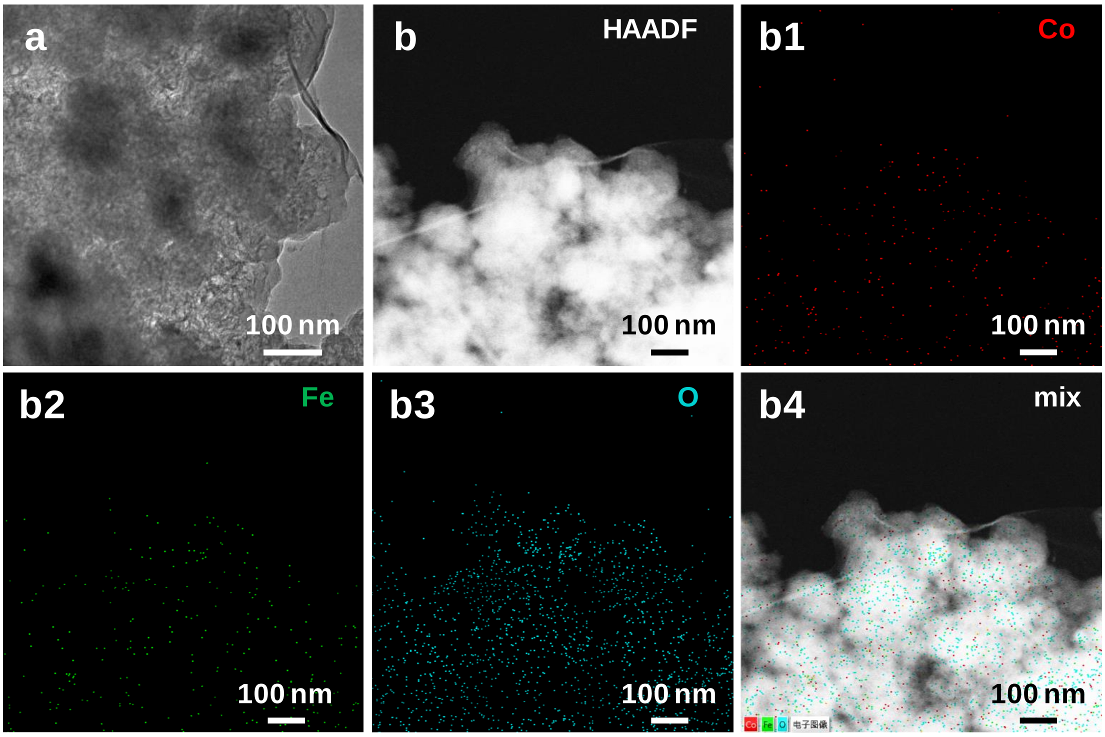

Supplementary Fig. 14 TEM images and the corresponding element mapping images of (Co,Fe)-OH sample. **a** TEM image. **b** HAADF image. **b1-b4** The corresponding element mapping images.

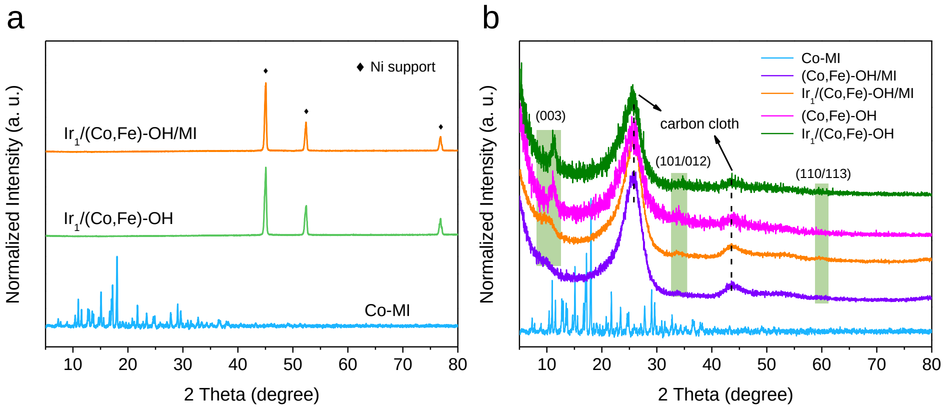

Supplementary Fig. 15 XRD patterns of (Co,Fe)-OH/MI, Ir$_1$/(Co,Fe)-OH/MI, (Co,Fe)-OH, Ir$_1$/(Co,Fe)-OH, and Co-MI. a The target samples prepared on Ni foam support. **b** The target samples and reference samples prepared on carbon cloth.

The XRD patterns in Supplementary Fig. 15b show the characteristic diffraction peaks of metal hydroxides for both target and reference samples prepared on carbon cloth.

Target samples: Ir$_1$/(Co,Fe)-OH and Ir$_1$/(Co,Fe)-OH/MI

Reference samples: (Co,Fe)-OH and (Co,Fe)-OH/MI

The characteristic peaks for the target samples are at around 9.9°, 33.6°, and 59.6°, corresponding to the (003), (101/012), and (110/113) lattice planes, respectively[20]. The reference samples have characteristic peaks at 11.1°, 33.9°, and 59.7°, corresponding to the same lattice planes[20].

The slight differences in peak positions are due to variations in interlayer distances between the target and reference samples[20,21]. The peaks at 25.5° and 43.6° are assigned to the carbon cloth support.

Note: The strong signals from the Ni foam used in the initial preparation (Supplementary Fig. 15a) obscured the target sample peaks. We prepared the target and reference samples on carbon cloth to understand the results.

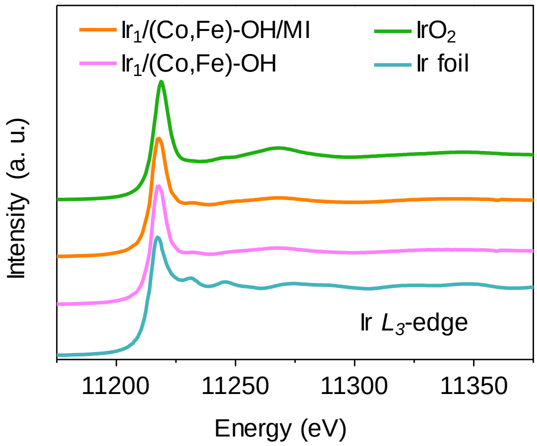

Supplementary Fig. 16 The normalized Ir L_3-edge XANES spectra of Ir_1/(Co,Fe)-OH/MI, Ir_1/(Co,Fe)-OH, IrO_2, and Ir foil.

Prior to measuring the Ir L_3-edge XAFS spectra of Ir_1/(Co,Fe)-OH/MI, Ir_1/(Co,Fe)-OH, and IrO_2, the XAFS spectrum of Ir foil was used for instrument calibration. This step is essential to eliminate energy shifts between experimental and theoretical spectra, which can arise due to beamline variations across synchrotron radiation facilities. The energy shift in Ir XAFS spectra was determined by calculating the difference between the measured X-ray absorption edge energy (E_0, corresponding to the energy at the first maximum in the first-order derivative of XANES) and its theoretical value (11215 eV for the Ir L_3-edge). Following the Ir foil measurement, the energy shift (ΔE_0) of the Ir L_3-edge XAFS was obtained by subtracting the theoretical E_0 from the measured E_0. The Ir L_3-edge XAFS spectra of Ir_1/(Co,Fe)-OH/MI, Ir_1/(Co,Fe)-OH, and IrO_2 were then automatically corrected by applying the ΔE_0 energy shift.

Following the automatic instrument correction, the Ir L_3-edge XAFS spectra of Ir_1/(Co,Fe)-OH/MI, Ir_1/(Co,Fe)-OH, IrO_2, and Ir foil were imported into Athena software for analysis. Prior to analysis, an E_0 value was defined within Athena for calibration purposes. Consistent with the literature[22,23], the energy at the first maximum position in the first-order derivative of the XANES spectrum was designated as E_0.

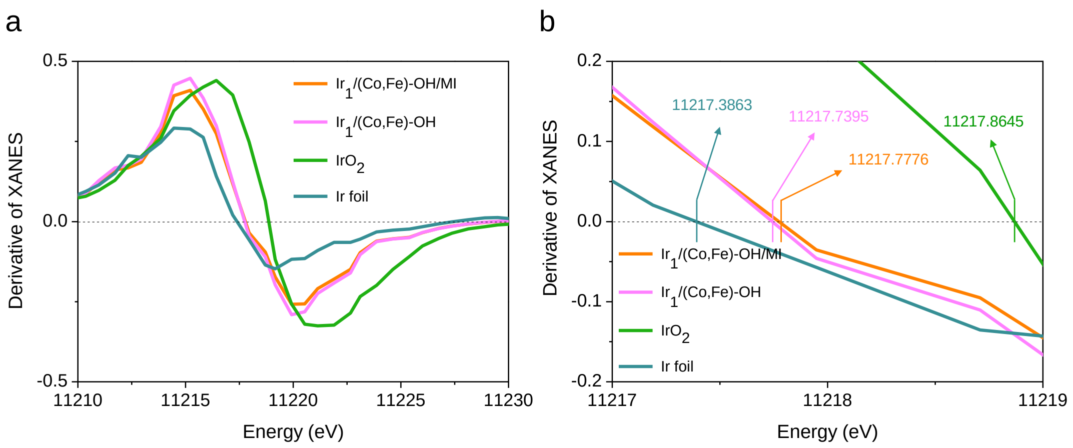

Supplementary Fig. 17 The first-order derivative of normalized Ir L_3-edge XANES spectra of Ir_1/(Co,Fe)-OH/MI, Ir_1/(Co,Fe)-OH, IrO_2, and Ir foil. a The first-order derivative of the normalized XANES spectra. **b** Zoom of the first-order derivatives of the XANES spectra near their zeros. Note: The normalized Ir L_3-edge XANES spectra are shown in Supplementary Fig. 16.

For 5d transition metals like Ir, the energy value at the white line peak in the normalized XANES spectrum provides insights into unoccupied electron states[24]. A shift of this peak towards higher energy indicates an increased valence state[24–28]. To determine the Ir oxidation states in the studied materials, the energy values at the white line peak were obtained by identifying the energies for which the first-order derivative of the corresponding normalized XANES spectra is zero. A linear prediction curve was constructed using the energy values at the white line peak positions of IrO_2 and Ir foil as the horizontal axis (independent variable), and their known oxidation states as the vertical axis (dependent variable) (Fig. 2c). The Ir oxidation states in Ir_1/(Co,Fe)-OH/MI and Ir_1/(Co,Fe)-OH were then estimated based on their measured white line peak energies and this prediction curve (Fig. 2c)[28,29].

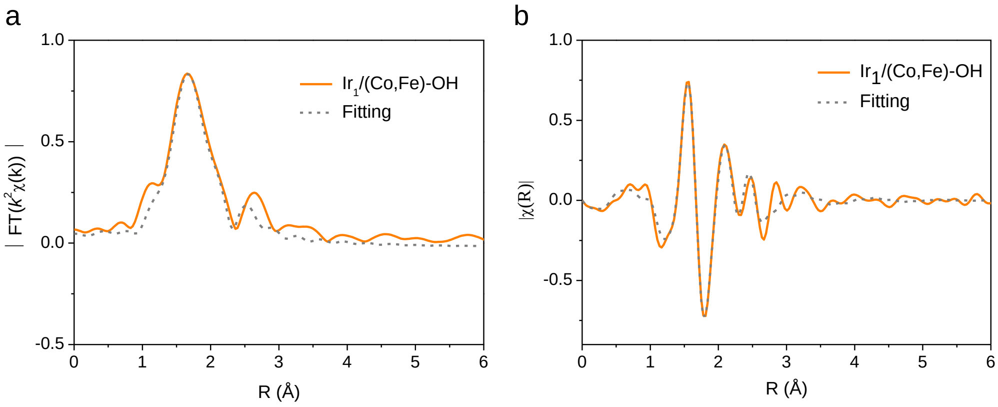

c

Ir L_3 edge EXAFS fitting results

Sample	Scattering path	Distance (Å)	C.N.	σ^2 ($Å^2$)	ΔE_0 (eV)
Ir foil	Ir-Ir	2.71	12	0.003	8.5
Ir_1/(Co,Fe)-OH/MI	Ir-O/N	2.03	3.0	0.006	11.3
	Ir-Fe/Co	2.59	0.9	0.006	11.3
Ir_1/(Co,Fe)-OH	Ir-O	2.03	2.4	0.006	10.4
	Ir-Fe/Co	2.57	1.9	0.006	10.4

S_0^2=0.7

Supplementary Fig. 18 The Ir *L_3* edge EXAFS fitting results. a,b The Ir L_3-edge EXAFS fitting results of Ir_1/(Co,Fe)-OH. **c** The fitting results of Ir_1/(Co,Fe)-OH/MI and Ir_1/(Co,Fe)-OH samples.

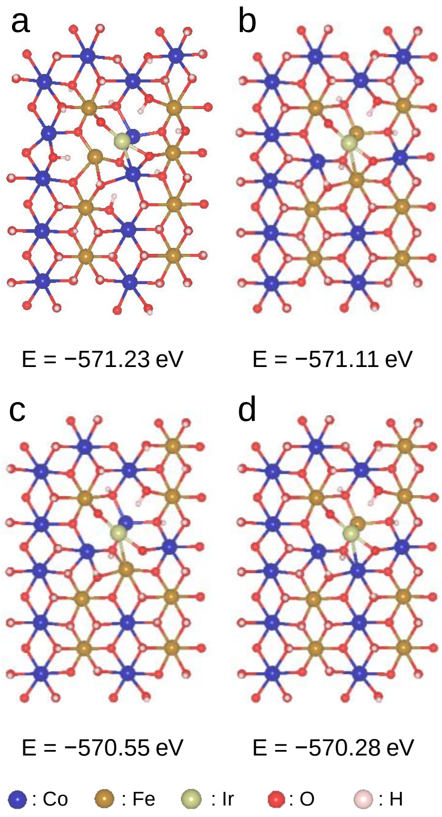

Supplementary Fig. 19 Possible structural models of the Ir$_1$/(Co,Fe)-OH sample. a One Ir atom coordinated with two O atoms and two Co atoms. **b** One Ir atom coordinated with two O atoms and two Fe atoms. **c,d** One Ir atom coordinated with two O atoms, one Co atom, and one Fe atom. Note: E represents the total energy of the system.

The Ir L_3-edge EXAFS results suggest that the Ir in the Ir$_1$/(Co,Fe)-OH sample is coordinated to two O atoms and two metal atoms (Co or Fe). The corresponding four possible structures are shown in Supplementary Fig. 19. Among these, the configuration in Supplementary Fig. 19a has the lowest total energy (−571.23 eV), implying the highest structural stability. Therefore, we used it as the basis for subsequent simulations of Ir$_1$/(Co,Fe)-OH and Ir$_1$/(Co,Fe)-OH/MI.

We refer to the configuration in Supplementary Fig. 19a as the Ir-CoFe model. The corresponding configuration with the MI molecule coordinated to the Ir atom is denoted as Ir(N)-CoFe (Supplementary Fig. 20).

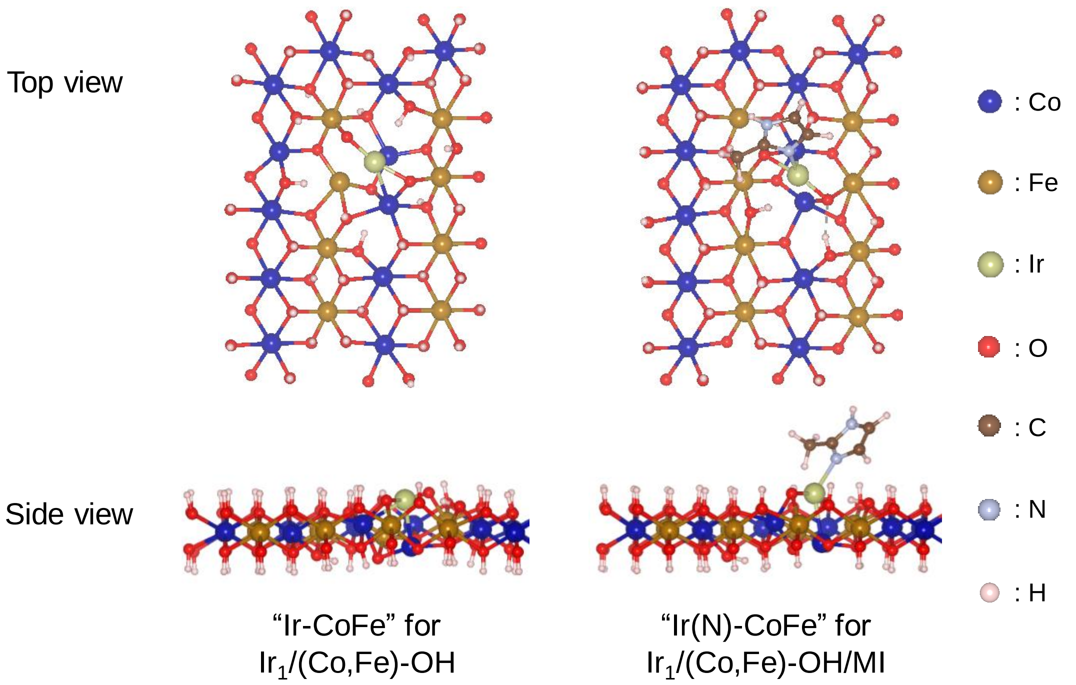

Supplementary Fig. 20 Atomic models of the Ir_1/(Co,Fe)-OH and Ir_1/(Co,Fe)-OH/MI samples.

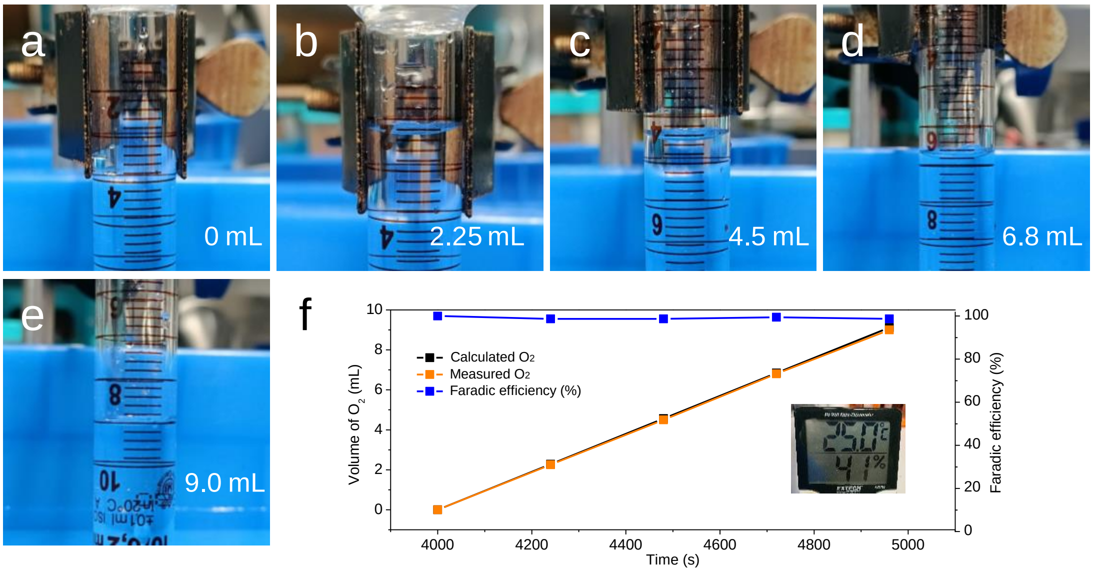

Supplementary Fig. 21 O_2 Production During OER Tests. a-e Digital photographs of the OER test setup. **f** Measured and calculated O_2 volume and Faradaic efficiency during OER tests. The O_2 evolution volume was measured every 4 minutes at a constant current density of 300 mA cm^{-2}.

The theoretical O_2 evolution volume ($V_{cal.}$) during the oxygen evolution reaction (OER) was calculated using the following equations, which account for key reaction parameters:

$$V_{cal.} = (I \times A \times t)/(\alpha F) \times V_{mol.} \quad (12a)$$

$$V_{mol.} = (n \times R \times T)/P \quad (12b)$$

where $V_{cal.}$ is the calculated O_2 volume (L), I is the OER current density (0.3 A cm^{-2});
A is the electrode area (0.5 cm^2), t is the OER reaction time (240 s); F is the Faraday constant (96485 C mol^{-1}), α is the number of electrons consumed per O_2 molecule (4 for OER), $V_{mol.}$ is the molar volume (L), n is the number of moles of O_2 (1 mol), R is the gas constant (8.314 J mol^{-1} K^{-1}), T is the temperature (K), and P is the atmospheric pressure (101325 Pa).

The Faradaic efficiency is given by

$$\text{Faradaic efficiency (\%)} = \left(\frac{V_{act.}}{V_{cal.}}\right) \times 100\% \quad (13)$$

where $V_{act.}$ is the experimentally measured O_2 volume.

The experimentally measured O_2 volume closely matched the theoretically calculated O_2 volume. Additionally, the Faradaic efficiency of the OER consistently exceeded 98% (see Supplementary Fig. 21f), signifying a highly efficient O_2 evolution process.

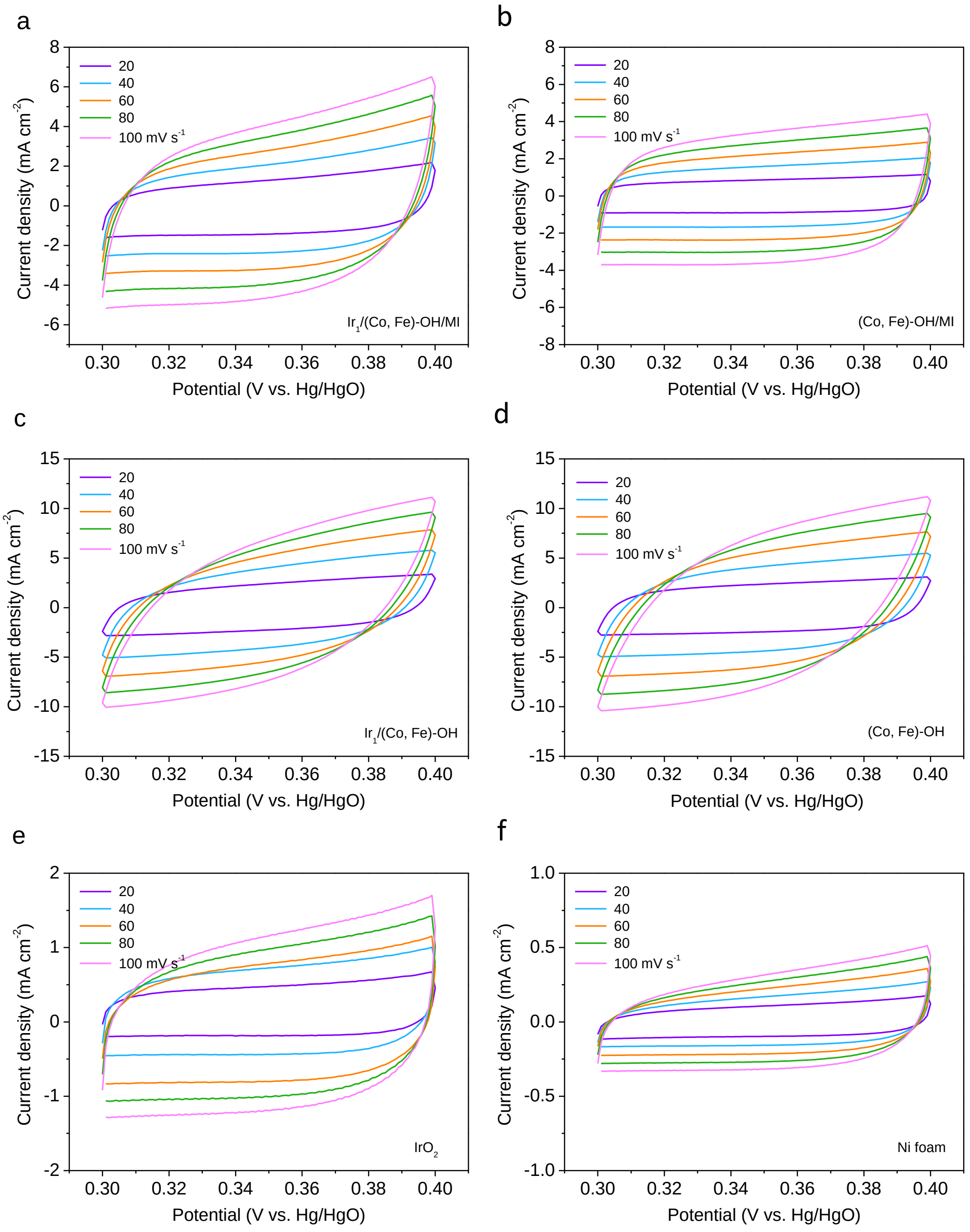

Supplementary Fig. 22 The CV curves of samples with different scan rates of 20, 40, 60, 80, and 100 mV s^{-1}. **a** Ir_1/(Co,Fe)-OH/MI. **b** (Co,Fe)-OH/MI. **c** Ir_1/(Co,Fe)-OH. **d** (Co,Fe)-OH. **e** IrO_2. **f** Ni foam.

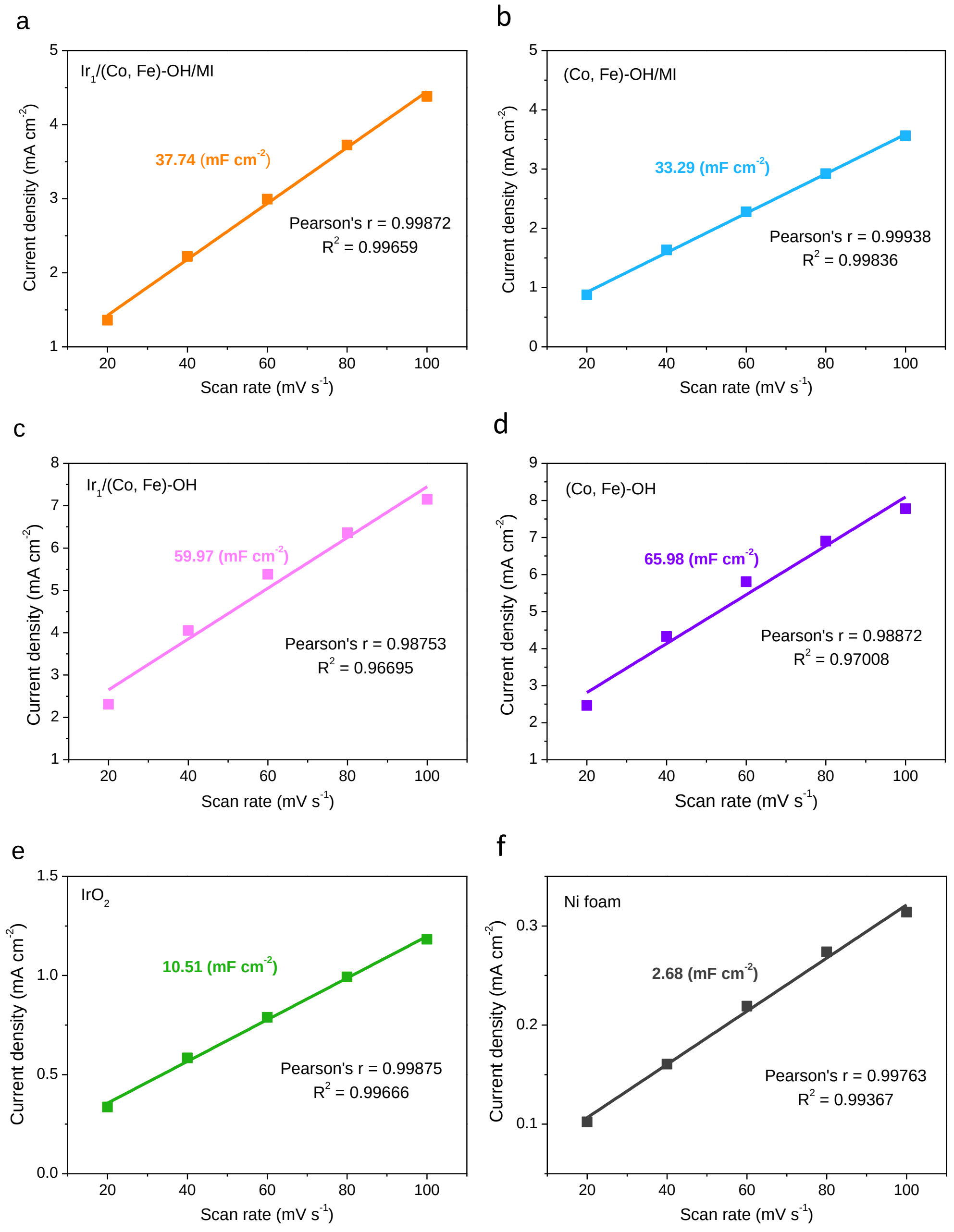

Supplementary Fig. 23 The C_{dl} of samples. a Ir_1/(Co,Fe)-OH/MI. **b** (Co,Fe)-OH/MI. **c** Ir_1/(Co,Fe)-OH. **d** (Co,Fe)-OH. **e** IrO_2. **f** Ni foam.

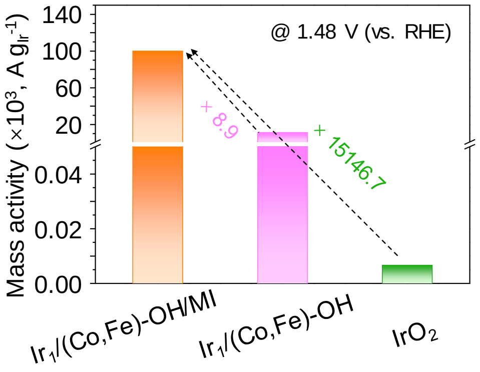

Supplementary Fig. 24 Mass activity based on Ir mass.

The mass activity of the Ir_1/(Co,Fe)-OH/MI catalyst, based on Ir mass, reaches 10^5 A g_{Ir}^{-1} (Supplementary Fig. 24). This value outperforms Ir_1/(Co,Fe)-OH by nearly 8.9 times and IrO_2 by over 15,000 times, demonstrating a significant enhancement in catalytic activity for oxygen evolution.

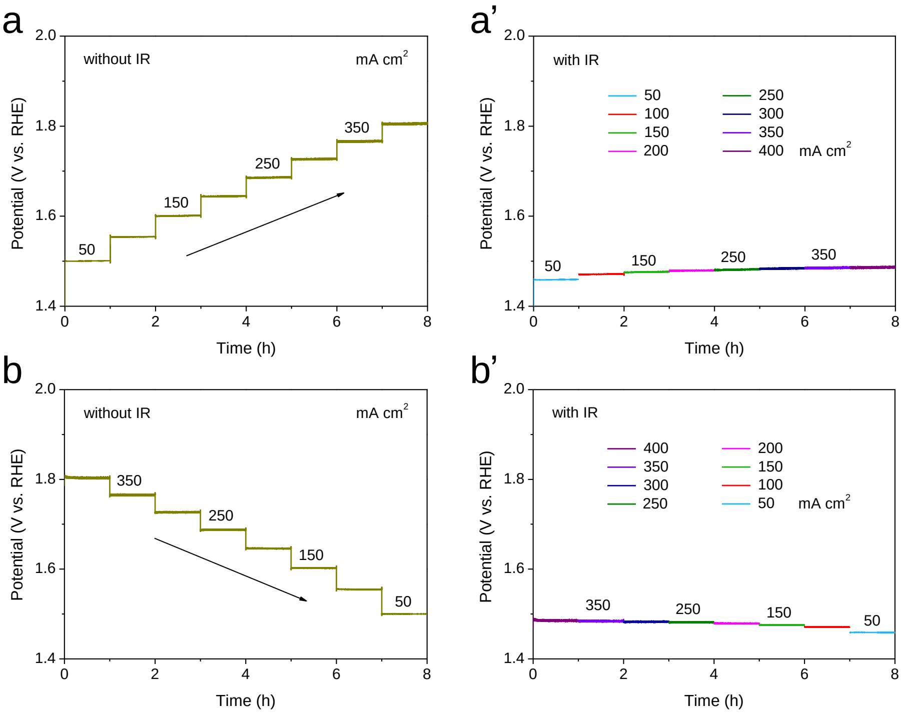

Supplementary Fig. 25 Multistep chronopotentiometry measurements were conducted to evaluate the OER stability of the Ir_1/(Co,Fe)-OH/MI sample. The current density varied within the 50−400 mA cm^{-2} range, with incremental or decremental steps of 50 mA cm^{-2} per hour. **a,a'** Current density increased from 50 to 400 mA cm^{-2}. **b,b'** Current density decreased from 400 to 50 mA cm^{-2}. Note: The potential values in **a** and **b** are not iR-compensated, while those in **a'** and **b'** are iR-compensated.

In the multistep chronopotentiometry measurements shown in Supplementary Fig. 25a, the current density was incrementally increased from 50 to 400 mA cm^{-2} with 50 mA cm^{-2} steps every hour, and the corresponding potentials were recorded. Upon reaching 50 mA cm^{-2}, the potential rapidly stabilized at approximately 1.501 V and remained constant for the remaining hour. Similar behavior was observed for all tested current densities up to 400 mA cm^{-2}.

In Supplementary Fig. 25b, the current density was decreased from 400 to 50 mA cm^{-2} with 50 mA cm^{-2} decrements every hour, and the corresponding potentials were recorded. At 400 mA cm^{-2}, the potential stabilized at approximately 1.804 V and remained constant for the remaining hour. Stable potential values were also observed across the current density range of 350−50 mA cm^{-2}.

Overall, the multistep chronopotentiometry measurements with incremental/decremental steps of 50 mA cm^{-2} per hour demonstrated consistent and stable potential values throughout each one-hour test (Supplementary Fig. 25a, b). These results indicate the good stability of the Ir_1/(Co,Fe)-OH/MI catalyst[30–32].

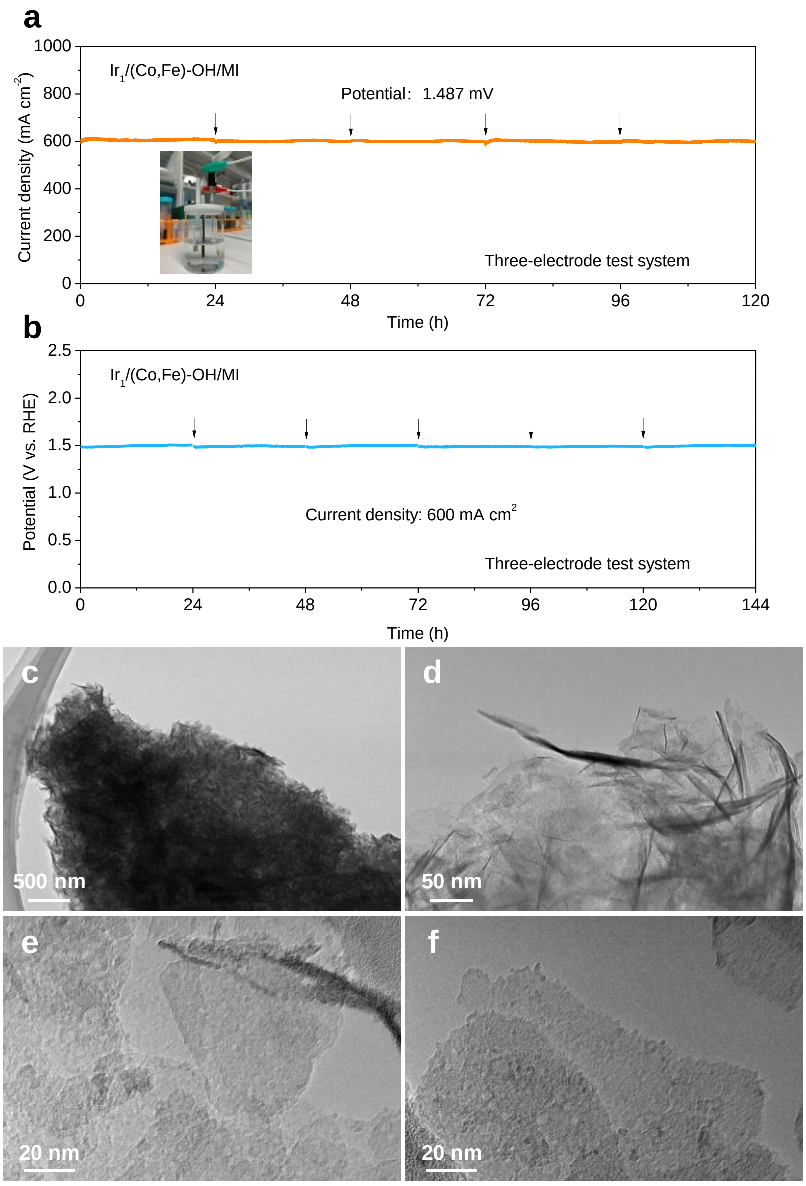

Supplementary Fig. 26 Stability characterizations of the Ir₁/(Co,Fe)-OH/MI sample in a three-electrode test system. a Chronoamperometric measurements. **b** Chronopotentiometric measurements. **c-f** TEM images of the catalyst after the OER chronoamperometric chracterization for 120 h at 600 mA cm^{-2}. Note: The potential data displayed in a and b are 100% iR-compensated. Arrows in **a** and **b** indicate fresh electrolyte replacements.

Chronoamperometry tests showed that Ir_1/(Co,Fe)-OH/MI can operate stably for 120 hours in a three-electrode system at a current density of 600 mA cm^{-2} (Supplementary Fig. 26a). Chronopotentiometry tests further confirmed this stability, showing consistent responses over 120 hours (Supplementary Fig. 26b). Post-OER analysis (Fig. 1 and Supplementary Fig. 26c-f) revealed no morphological changes in the catalyst.

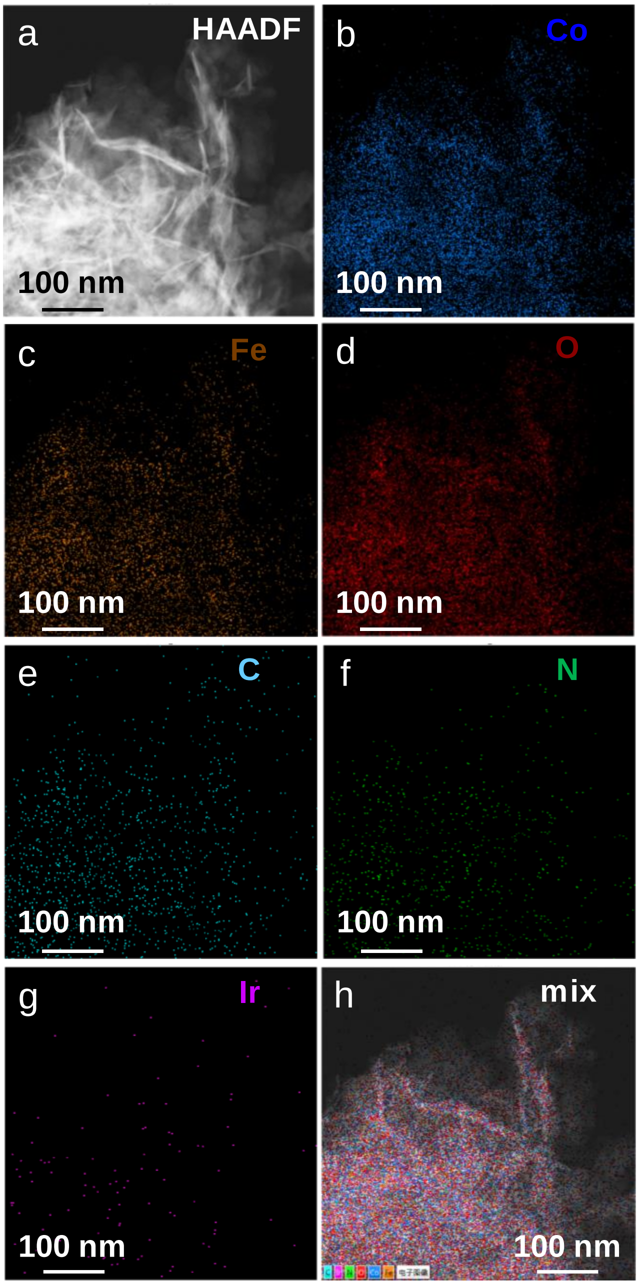

Supplementary Fig. 27 The HAADF-TEM image and corresponding element mappings of the Ir_1/(Co,Fe)-OH/MI sample after OER reaction for 120 h at 600 mA cm^{-2}. a HAADF image. **b-h** Element maps.

HAADF-TEM imaging and elemental mapping (Supplementary Fig. 27) were performed to investigate the stability of the Ir_1/(Co,Fe)-OH/MI sample after OER operation for 120 hours at a current density of 600 mA cm^{-2}. The element maps (Supplementary Fig. 27b-h) reveal that the distribution of elements within the Ir_1/(Co,Fe)-OH/MI sample remains largely unchanged compared to the pristine sample (Fig. 1), indicating good structural integrity. Notably, the presence of nitrogen (N) and iridium (Ir) signals (Supplementary Fig. 27f, g) confirms the persistence of both MI organic molecules and Ir atoms within the catalyst after extended OER operation.

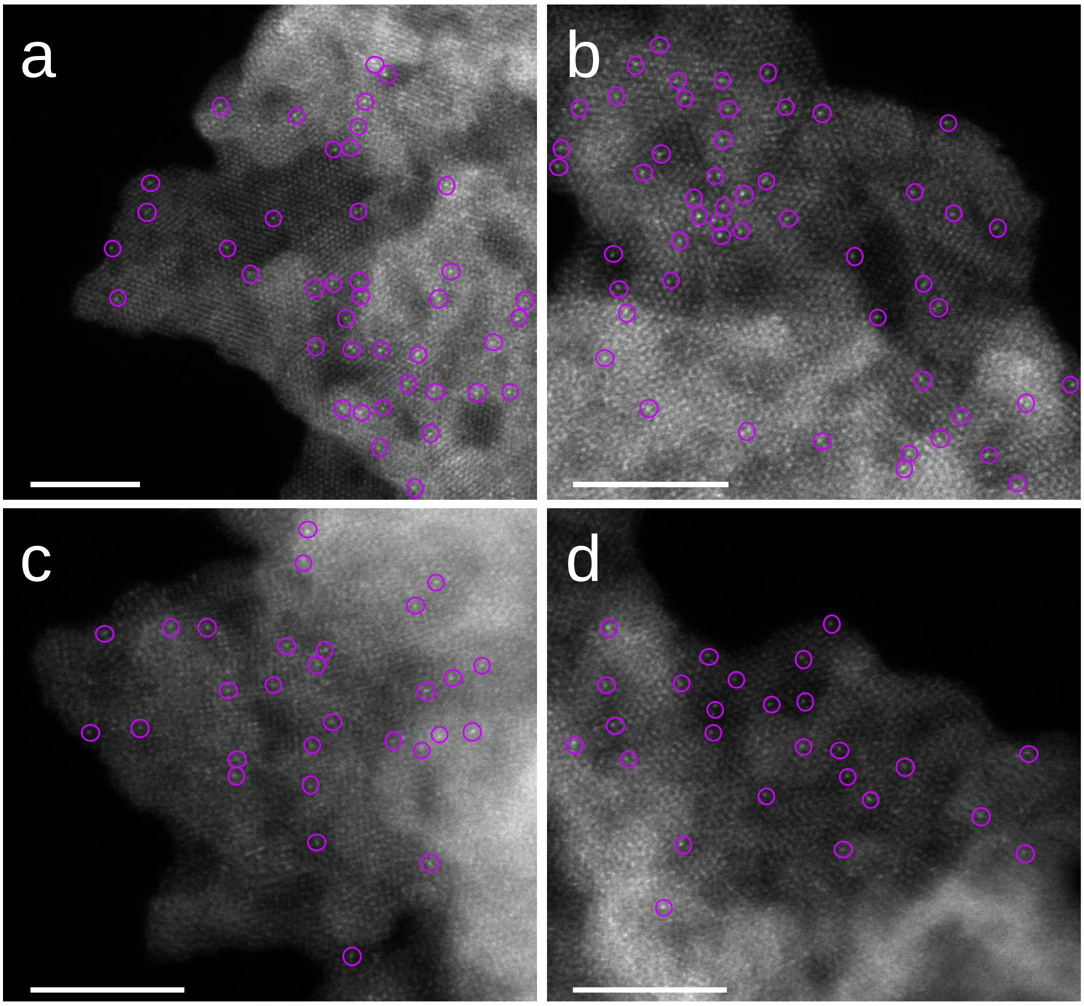

Supplementary Fig. 28 HAADF-STEM images of the Ir_1/(Co,Fe)-OH/MI sample after OER reaction for 120 h at 600 mA cm^{-2}. a-d The HAADF-STEM images at different positions of the Ir_1/(Co,Fe)-OH/MI sample. The scale bar is 5 nm.

HAADF-STEM was employed to analyze the morphology of Ir species in the Ir_1/(Co,Fe)-OH/MI sample after OER operation for 120 hours at 600 mA cm^{-2} (Supplementary Fig. 28). The HAADF-STEM images at various locations (Supplementary Fig. 28) show that Ir species remain as isolated individual atoms, consistent with observations of the pristine sample (Fig. 1).

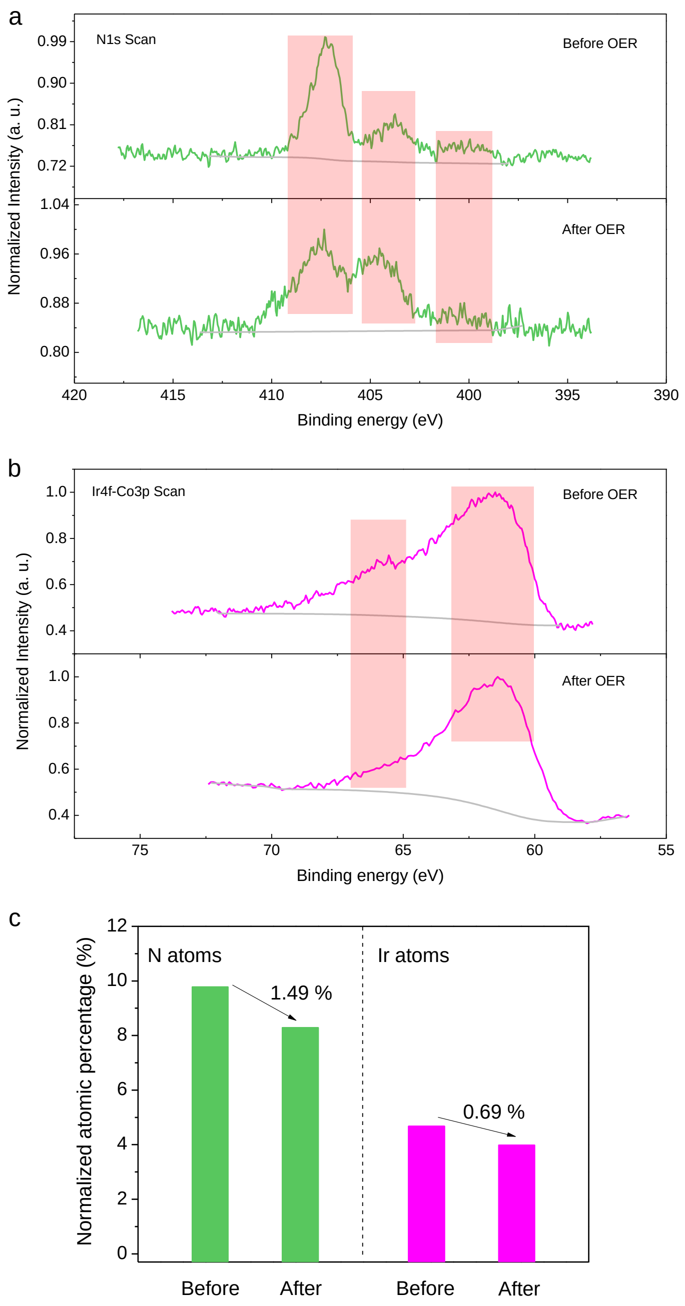

Supplementary Fig. 29 XPS results of the Ir$_1$/(Co,Fe)-OH/MI sample before and after the OER reaction for 120 hours at 600 mA cm^{-2}. a N 1s XPS. **b** Ir 4f-Co 3p XPS. **c** Normalized atomic percentages of N and Ir atoms before and after the OER reaction.

XPS analysis revealed small decreases in the normalized atomic percentages of N (1.49% reduction from 9.78% to 8.29%) and Ir (0.69% reduction from 4.68% to 3.99%) after the OER testing for 120 hours at 600 mA cm^{-2} (Supplementary Fig. 29). These minor decreases had a negligible effect on the catalyst's stability during extended OER operation. The decreased N/Ir atomic ratio was approximately 2.16 (1.49% / 0.69% ≈ 2.16). Since a single MI molecule contains two N atoms, this suggests a near 1:1 molar ratio loss of MI and Ir, implying the loss occurred as an "MI-Ir" unit rather than through the cleavage of the chemical bond between MI and Ir. This observation indicates strong coordination between MI and Ir, consistent with simulation results (Supplementary Fig. 39). The loss of the Ir-MI unit likely arises from the reconstruction of Ir$_1$/(Co,Fe)-OH/MI (from hydroxide to oxyhydroxide) during the early stages of the OER test. To further investigate this Ir-MI coordination loss due to reconstruction, we analyzed

Ir_1/(Co,Fe)-OH/MI using the in-situ Raman spectroscopy at various potentials (Supplementary Fig. 30).

Note:

The normalized atomic percentage was calculated base on the Co, Fe, N, and Ir atomic ratios. The corresponding calculation formulas are as follows.

$$\alpha_N = \frac{\chi_N}{\chi_N + \chi_{Co} + \chi_{Fe} + \chi_{Ir}} \times 100\% \tag{14a}$$

$$\alpha_{Ir} = \frac{\chi_{Ir}}{\chi_N + \chi_{Co} + \chi_{Fe} + \chi_{Ir}} \times 100\% \tag{14b}$$

$$\beta_N = \frac{\psi_N}{\psi_N + \psi_{Co} + \psi_{Fe} + \psi_{Ir}} \times 100\% \tag{14c}$$

$$\beta_{Ir} = \frac{\psi_{Ir}}{\psi_N + \psi_{Co} + \psi_{Fe} + \psi_{Ir}} \times 100\% \tag{14d}$$

where:

- χ_N, χ_{Co}, χ_{Fe}, and χ_{Ir} are the N, Co, Fe, and Ir atomic ratios before OER, respectively.
- ψ_N, ψ_{Co}, ψ_{Fe}, and ψ_{Ir} are the N, Co, Fe, and Ir atomic ratios after OER, respectively.
- α_N and α_{Ir} are the normalized atomic percentage of N and Ir before OER, respectively.
- β_N and β_{Ir} are the normalized atomic percentahe of N and Ir after OER, respectively.

The atomic ratios of XPS are different from those of ICP-MS, mainly because the XPS technique analyzes the components on the surface and near the surface of the materials. The ICP-MS technology analyzes the components of the whole material, including surface, near the surface, and bulk phase.

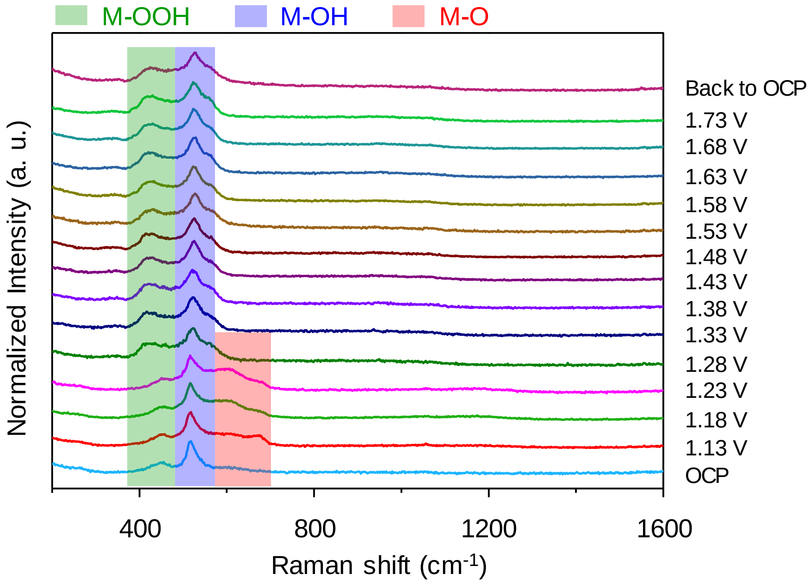

Supplementary Fig. 30 ***In situ*** **Raman spectra of Ir_1/(Co,Fe)-OH/MI sample at different potentials during OER reaction.** Note: M is Co or Fe.

In-situ Raman spectroscopy was conducted to probe the structural evolution of the Ir_1/(Co,Fe)-OH/MI catalyst during OER (Supplementary Fig. 30). The spectra were collected after chronoamperometry measurements at various potentials ranging from 1.13 to 1.73 V vs. RHE.

The results reveal a clear potential-dependent structural change within the catalyst. As the applied potential increased, the intensity of peaks corresponding to M-O bonds decreased, while those associated with M-OOH bonds became more prominent (Supplementary Fig. 30). These observations suggest that the Ir_1/(Co,Fe)-OH/MI undergoes a partial reconstruction from hydroxides to oxyhydroxides during OER.

The reconstruction process likely contributes to the loss of MI-Ir blocks. These findings suggest that future development of high-performance OER electrocatalysts should prioritize enhancing the stability of the hydroxide support while preserving the beneficial out-of-plane MI coordination.

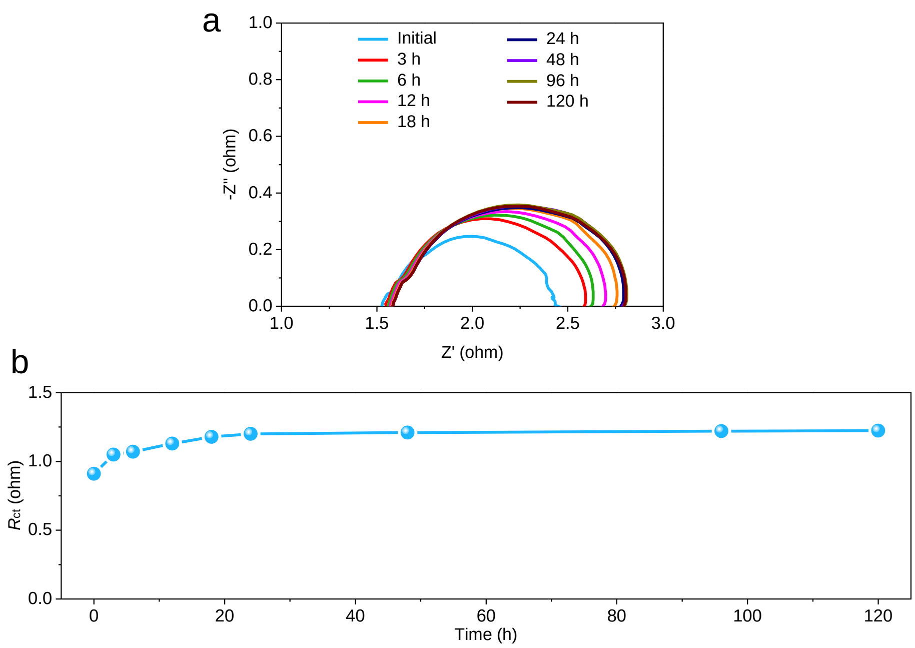

Supplementary Fig. 31 Electrochemical impedance spectroscopy of Ir_1/(Co,Fe)-OH/MI during a 120 h-long chronoamperometric test at 600 mA cm^{-2}. a Electrochemical impedance spectra as a function of time. **b** Charge transfer resistance (R_{ct}) evolution as a function of time.

Electrochemical impedance spectroscopy (EIS) results revealed that the charge transfer resistance (R_{ct}) of Ir_1/(Co,Fe)-OH/MI increased (from 0.91 to 1.20 Ω) during the initial 24 hours of OER testing, likely due to the (partial) reconstruction of hydroxides to oxyhydroxides in Ir_1/(Co,Fe)-OH/MI as indicated in the Raman characterization (Supplementary Fig. 30). Subsequently, the R_{ct} remained stable (1.21~1.22 Ω) likely due to the stable coordination between the Ir and MI in the Ir_1/(Co,Fe)-OH/MI sample (Supplementary Fig. 31).

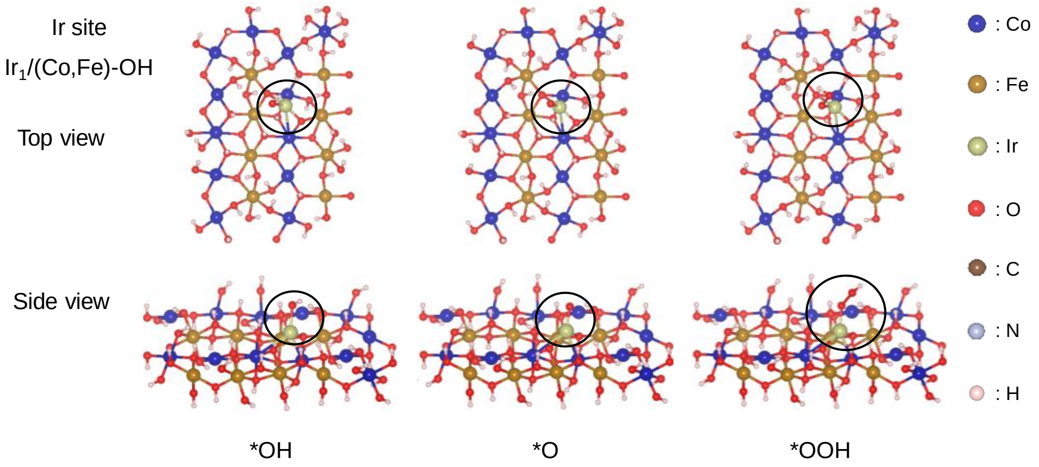

Supplementary Fig. 32 The structures adsorbed by OER intermediate species at the Ir site in Ir-CoFe model.
Note: The Ir-CoFe model corresponds to Ir_1/(Co,Fe)-OH sample.

The *OH, *O, and *OOH intermediates can be stably adsorbed at the Ir site of Ir-CoFe model.

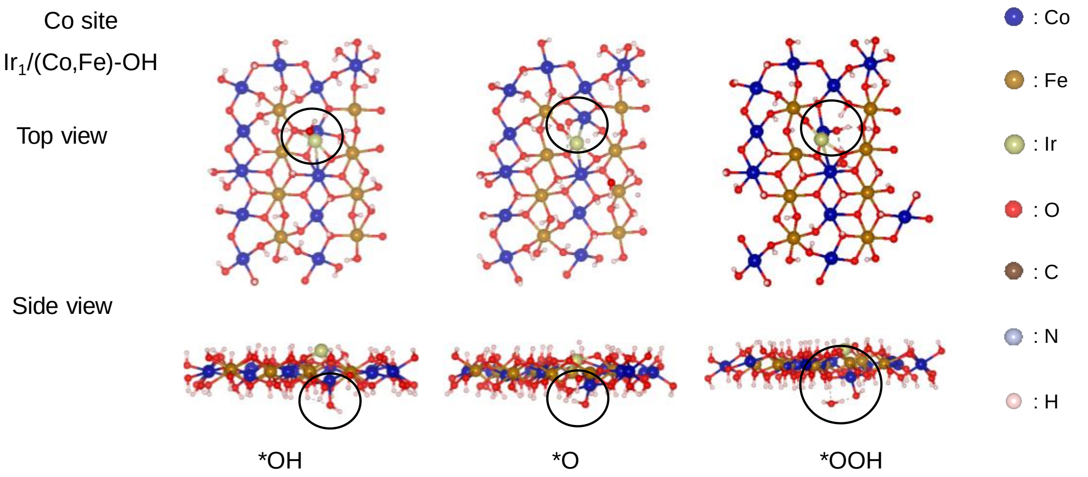

Supplementary Fig. 33 The structures adsorbed by OER intermediate species at the adjacent Co site near to Ir site in Ir-CoFe model. Note: The Ir-CoFe model corresponds to Ir_1/(Co,Fe)-OH sample.

In Ir-CoFe model, the *OH intermediate can be stably adsorbed at the adjacent Co site near to Ir site. But, the *O and *OOH intermediates cannot be stably adsorbed at this adjacent Co site.

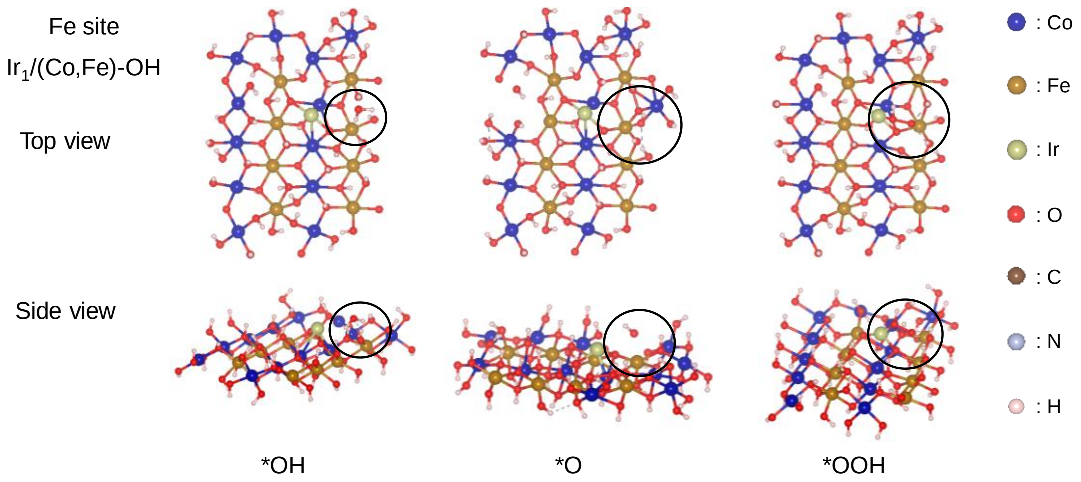

Supplementary Fig. 34 The structures adsorbed by OER intermediate species at the adjacent Fe site near to Ir site in Ir-CoFe model. Note: The Ir-CoFe model corresponds to Ir_1/(Co,Fe)-OH sample.

In Ir-CoFe model, the *OH, *O, and *OOH intermediates cannot be stably adsorbed at the adjacent Fe site near to Ir site.

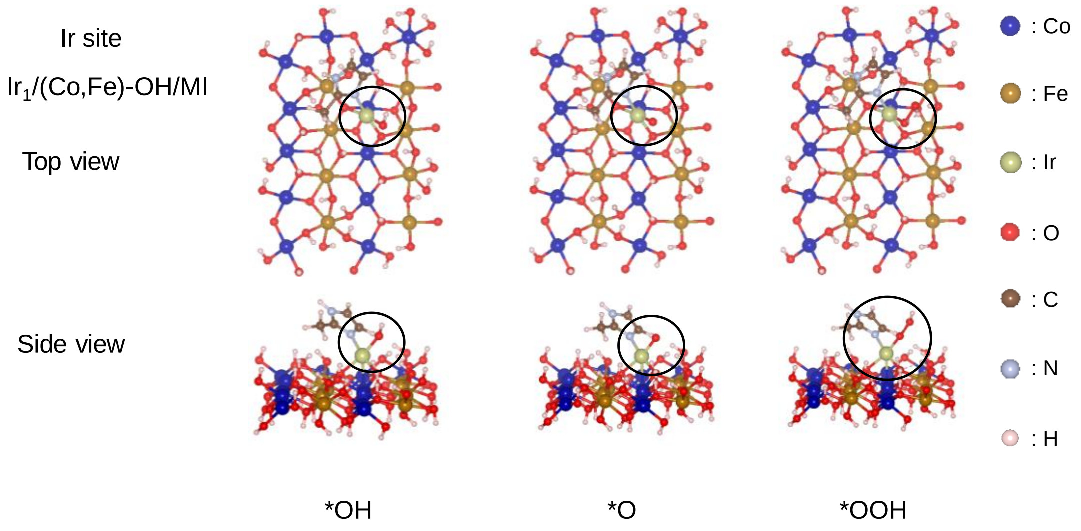

Supplementary Fig. 35 The structures adsorbed by OER intermediate species at Ir site in Ir(N)-CoFe model.
Note: The Ir(N)-CoFe model corresponds to Ir_1/(Co,Fe)-OH/MI sample.

In Ir(N)-CoFe model, the *OH, *O, and *OOH intermediates can be stably adsorbed at Ir site.

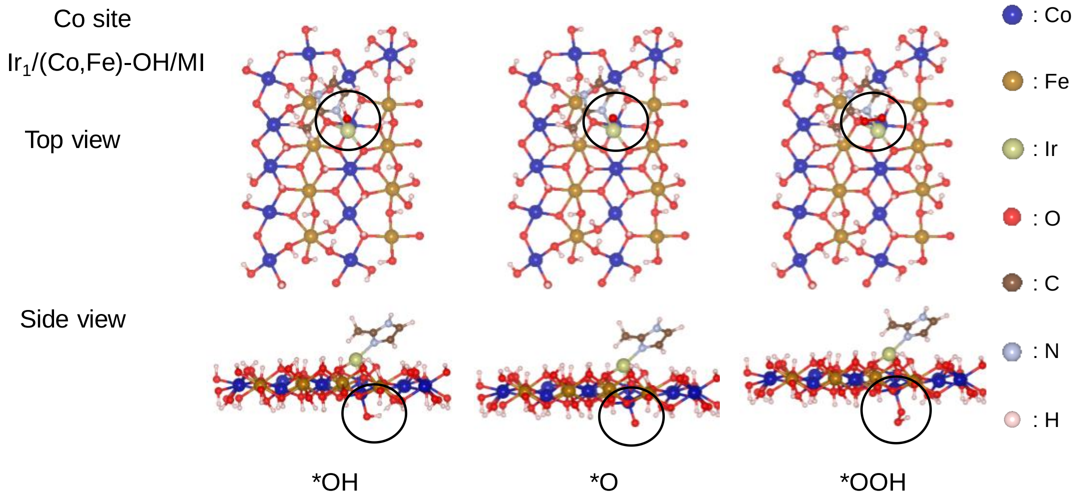

Supplementary Fig. 36 The structures adsorbed by OER intermediate species at the adjacent Co site near to Ir site in Ir(N)-CoFe model. Note: The Ir(N)-CoFe model corresponds to Ir_1/(Co,Fe)-OH/MI sample.

In Ir(N)-CoFe model, the *OH, *O, and *OOH intermediates can also be stably adsorbed at adjacent Co site near to Ir site.

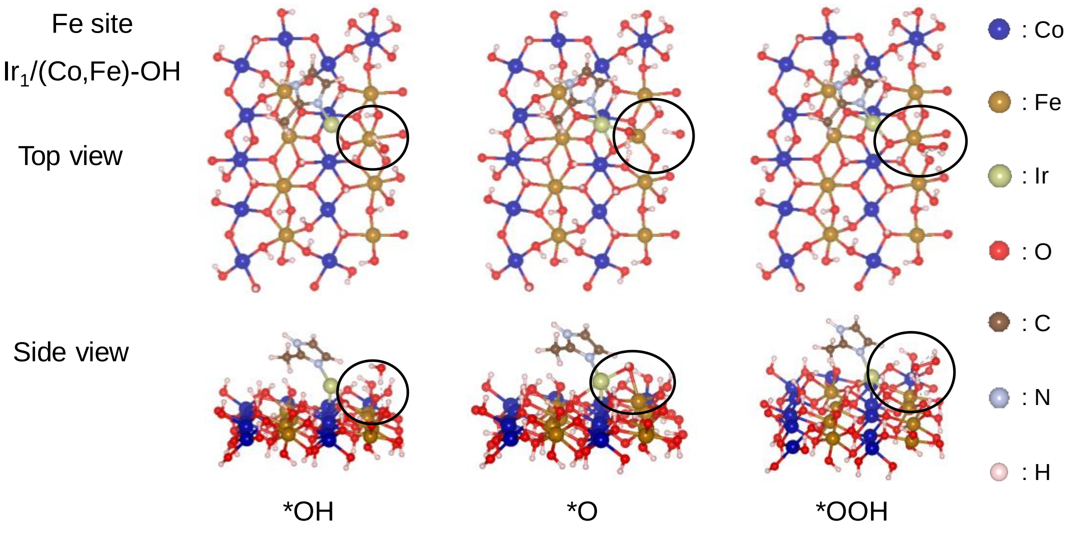

Supplementary Fig. 37 The structures adsorbed by OER intermediate species at the adjacent Fe site near to Ir site in Ir(N)-CoFe model. Note: The Ir(N)-CoFe model corresponds to Ir_1/(Co,Fe)-OH/MI sample.

In Ir(N)-CoFe model, the *OH, *O, and *OOH intermediates cannot be stably adsorbed at the adjacent Fe site near to Ir site.

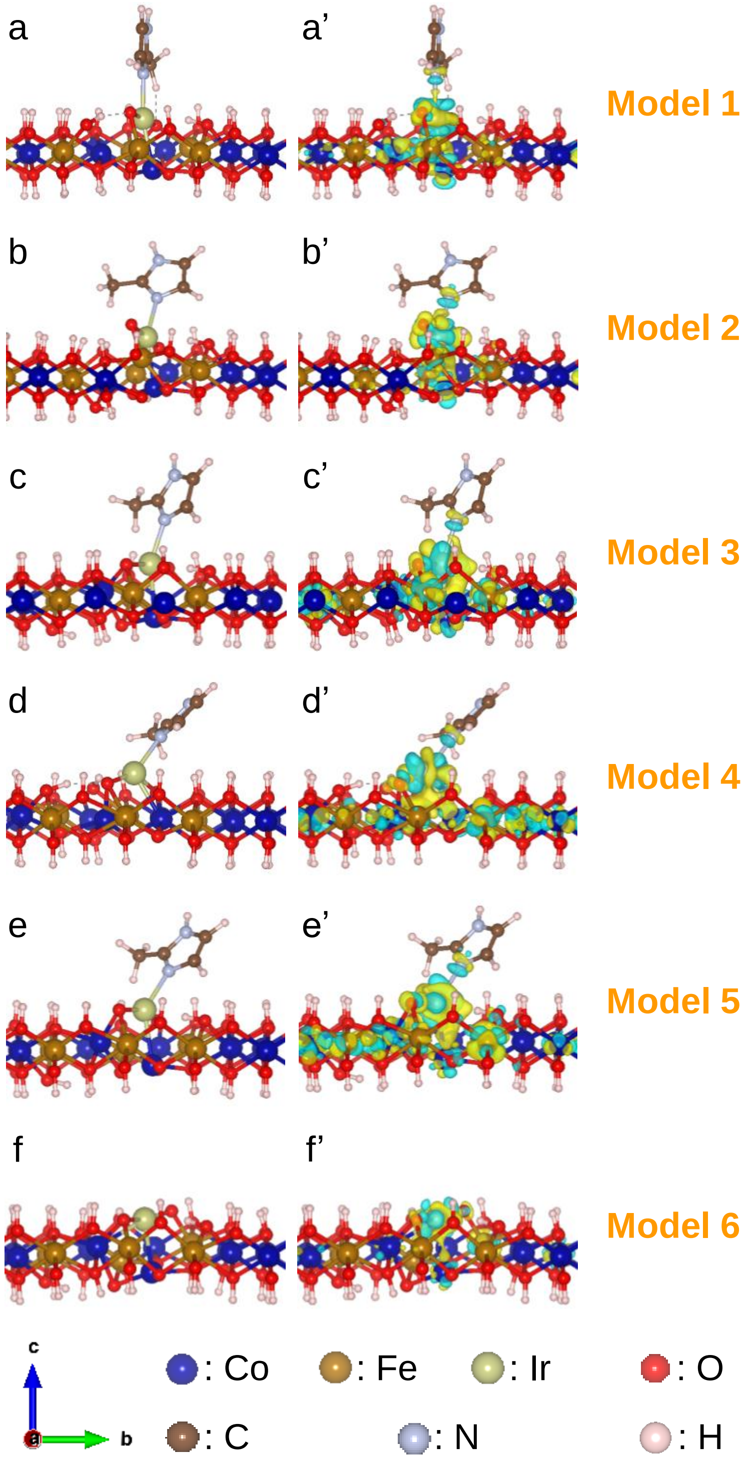

Supplementary Fig. 38 Differential charge density of Ir(N)-CoFe models with MI molecule coordinated at different orientations in Ir_1/(Co,Fe)-OH/MI, where the yellow and green regions represent electronic accumulation and depletion, respectively. The various Ir(N)-CoFe models with MI molecules coordinated at different orientations were denoted as Model 1, Model 2, Model 3, Model 4, and Model 5. The Ir-CoFe model in Ir_1/(Co,Fe)-OH was denoted as Model 6 for comparision. **a,a’** Model 1. **b,b’** Model 2. **c,c’** Model 3. **d,d’** Model 4. **e,e’** Model 5. and **f,f’** Model 6.

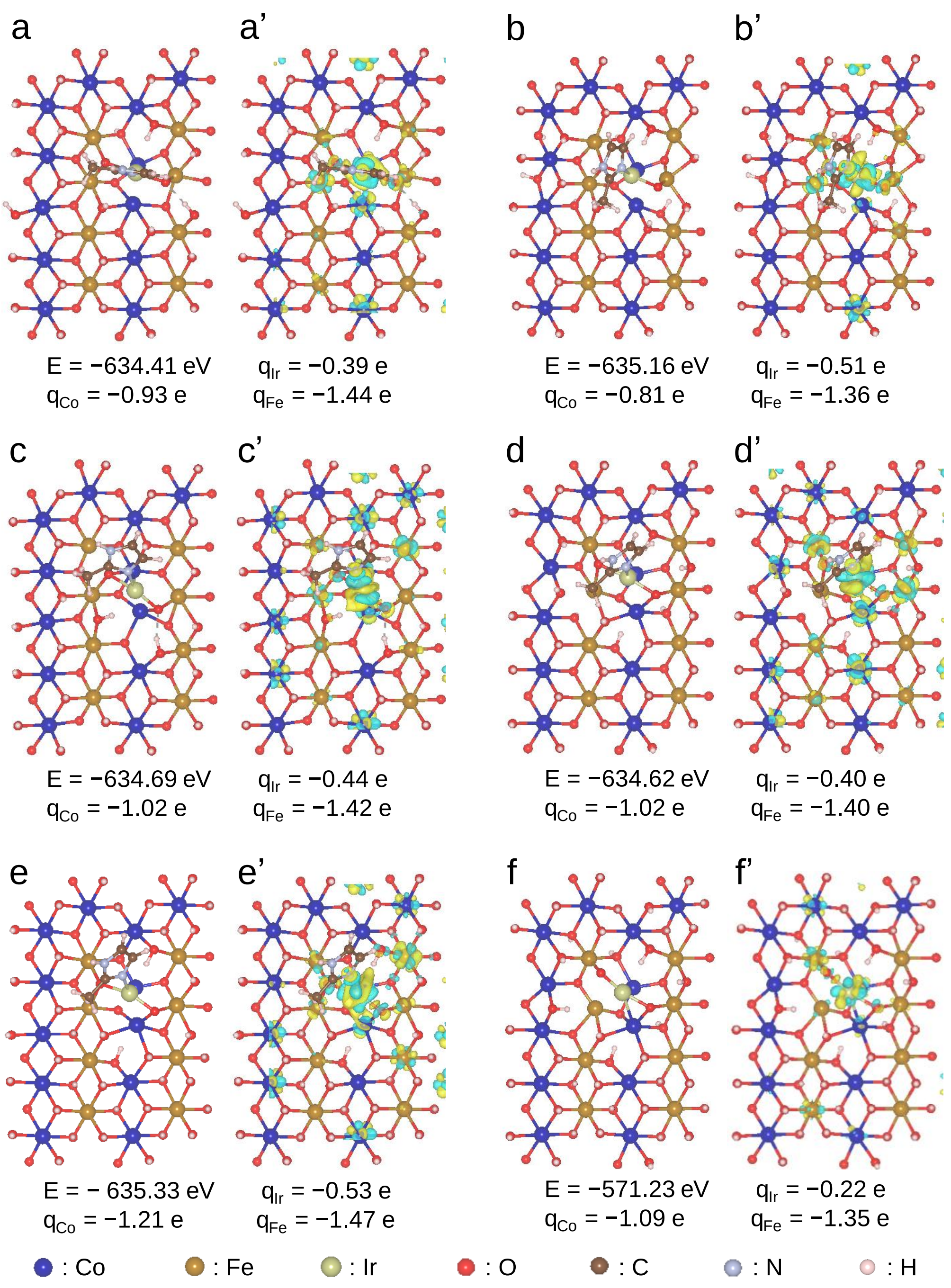

Supplementary Fig. 39 Differential charge density and Bader charge of Ir(N)-CoFe models with MI molecule coordination at different orientations in Ir_1/(Co,Fe)-OH/MI. Yellow and green regions represent electron accumulation and depletion, respectively. **a,a'** Model 1. **b,b'** Model 2. **c,c'** Model 3. **d,d'** Model 4. **e,e'** Model 5. **f,f'** Model 6. Note: These are top views (along the c-axis) of the models shown in Supplementary Fig. 38. E represents the total energy of the system. q_M (unit: e, M = Co, Fe, or Ir) is the average charge transfer number from metal atoms around the Ir atom. The Ir-CoFe for Ir_1/(Co,Fe)-OH is denoted as Model 6 for comparison.

Differential charge density results (Supplementary Fig. 38, 39) revealed that MI coordination (see Model 1~5 shown in Supplementary Fig. 38) induces charge redistribution around Ir atoms, creating pronounced electron donation regions. Additionally,

charge density around Fe and Co atoms was subtly altered, suggesting that MI orientation influences charge distributions between these metal atoms (Supplementary Fig. 39).

Bader charges were calculated to investigate the effect of MI molecule orientation on the charge distribution at the Ir, Co, and Fe sites in the Ir(N)-CoFe models (Supplementary Fig. 38, 39). In these figures, yellow regions indicate electron accumulation and green regions indicate depletion. The Ir(N)-CoFe models with varying MI orientations (Model 1~5) were analyzed and compared to a reference Ir-CoFe model (Model 6). Model 5, the Ir(N)-CoFe structure, exhibits the largest charge transfer values at the Ir, Co, and Fe sites, which are −0.53, −1.21, and −1.47e, respectively.

Generally, a lower system total energy corresponds to a more stable model structure. Compared to the reference Ir-CoFe model without MI coordination (Model 6), the Ir(N)-CoFe models with MI coordination (Model 1~5) exhibit a significant reduction in the total energy of the system, by about 63.18 to 64.10 eV (Supplementary Fig. 39). This finding demonstrates that MI coordination with Ir single atoms enhances the structural stability. Furthermore, the different MI orientations lead to variations in the energy values of the systems (Supplementary Fig. 39). Among these models with varying MI orientations, Model 5 exhibited the lowest system-energy value of −635.33 eV, indicating that it is the most stable structure. Therefore, the theoretical calculations and analysis of the Ir(N)-CoFe model in Fig. 4 were performed using Model 5. The reference Ir-CoFe model without MI coordination (denoted as Model 6) is also included in Fig. 4 for comparison.

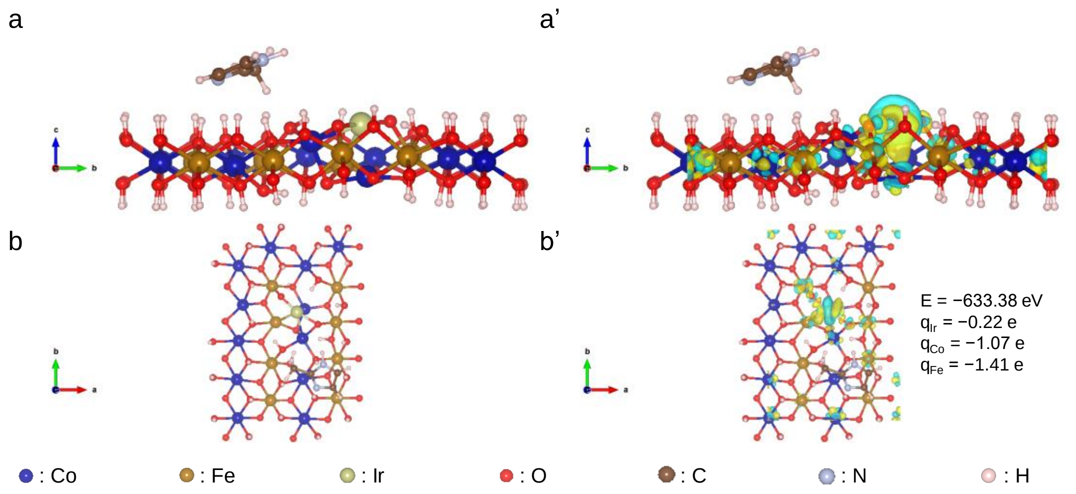

Supplementary Fig. 40 Differential charge density and Bader charge of Ir-CoFe model with adsorbed MI molecule. a,a' Side views. **b,b'** Top views.

To assess the impact of MI molecule coordination, we compared the MI adsorbed model (*i.e.*, the Ir-CoFe model with an adsorbed MI molecule, see Supplementary Fig. 40) with the MI coordinated model (*i.e.*, the Ir(N)-CoFe with a coordinated MI molecule at Ir atom site, see Supplementary Fig. 39e). The lower total energy of the MI coordinated model (−635.33 eV) compared to the MI adsorbed model (−633.38 eV) demonstrated that MI coordination with Ir is thermodynamically favored.

Next, to identify the optimal configuration among the coordinated MI models, we analyzed their total energies, differential charge density, Bader charge, partial density of states (PDOS), Gibbs free energy, overpotential, and reaction pathway. These analyses were performed based on the most stable Model 5 (Supplementary Fig. 38e).

A key difference emerged between the most stable Ir(N)-CoFe model (model 5, Supplementary Figs. 38e, 39e) and the Ir-CoFe model. In model 5, the coordinated MI molecule attracts electrons from Ir, Co, and Fe atoms, leading to increased electron donation from the metals and higher oxidation states compared to Ir-CoFe. This calculation result aligns well with the XAFS experiments (Fig. 2c and Supplementary Figs. 42d, 43d, 44).

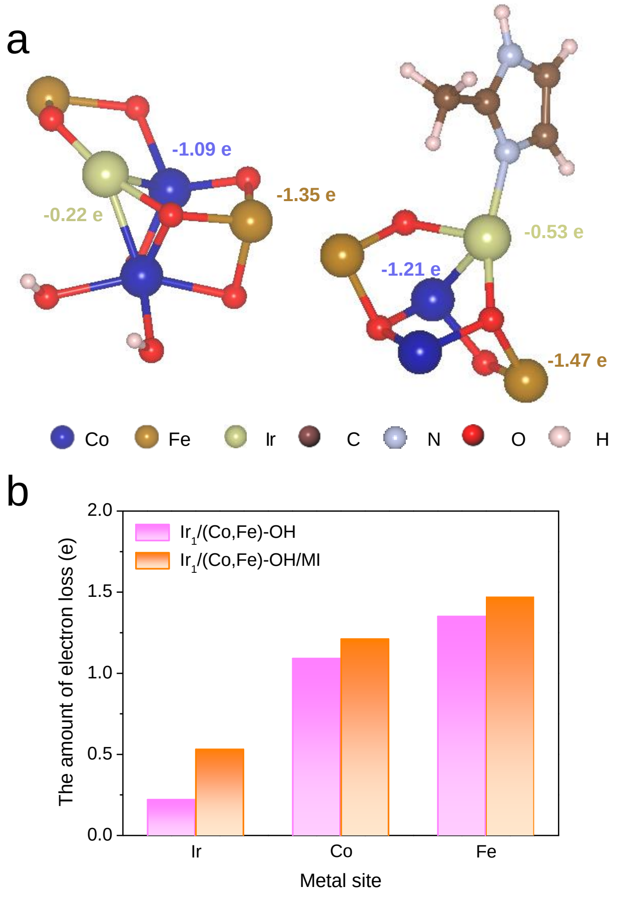

Supplementary Fig. 41 The Bader charge analysis of Ir(N)-CoFe and Ir-CoFe models in Ir_1/(Co,Fe)-OH/MI and Ir_1/(Co,Fe)-OH. a The structure models of Ir-CoFe and Ir(N)-CoFe. **b** The amount of charge missing at Ir, Co, and Fe sites.

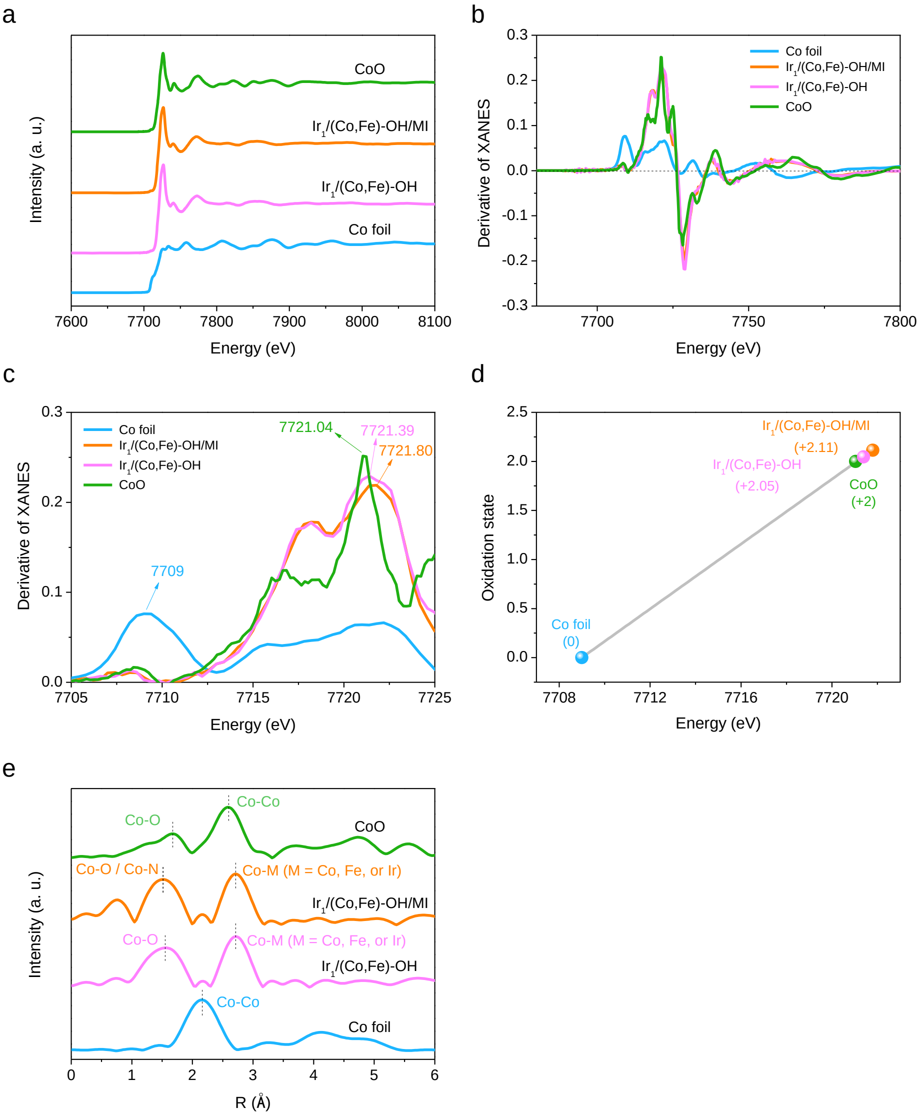

Supplementary Fig. 42 The results of Co *K*-edge XAFS. a XANES spectra. **b** The first derivative spectra. **c** The enlargement of first derivative spectra. **d** Valence of various Co species obtained from Co *K*-edge XANES. **e** The normalized Co *K*-edge FT-EXAFS spectra.

The synchrotron radiation instrument correction for Co *K*-edge XAFS spectra mirrored the procedure detailed for Ir L_3-edge XAFS spectra (see Supplementary Fig. 16). A Co foil was employed to eliminate energy shifts between experimental and theoretical spectra, arising from beamline variations. The theoretical X-ray absorption edge energy (E_0) for the Co *K*-edge XANES spectrum, which is determined as the energy of the first maximum in the XAFS first-order derivative, is 7709 eV.

For 3d transition metals like Co, the X-ray absorption edge energy (*i.e.*, E_0) in the normalized XANES spectra provides oxidation state information[22,23]. To determine the oxidation states of Co in Ir_1/(Co,Fe)-OH/MI, Ir_1/(Co,Fe)-OH, CoO, and the Co foil, we first obtained their respective E_0 values by identifying the first energy at which the first-order derivative of their XANES spectra reaches a maximum (Supplementary Fig. 42c). We then constructed a linear prediction curve using the E_0 of CoO and Co foil as the horizontal axis (independent variable/covariate) and their known oxidation states as the vertical axis (dependent variable/response) (Supplementary Fig. 42d). The oxidation states of Co in Ir_1/(Co,Fe)-OH/MI and Ir_1/(Co,Fe)-OH were then estimated based on the measured E_0 and the above-mentioned prediction curve[22,23].

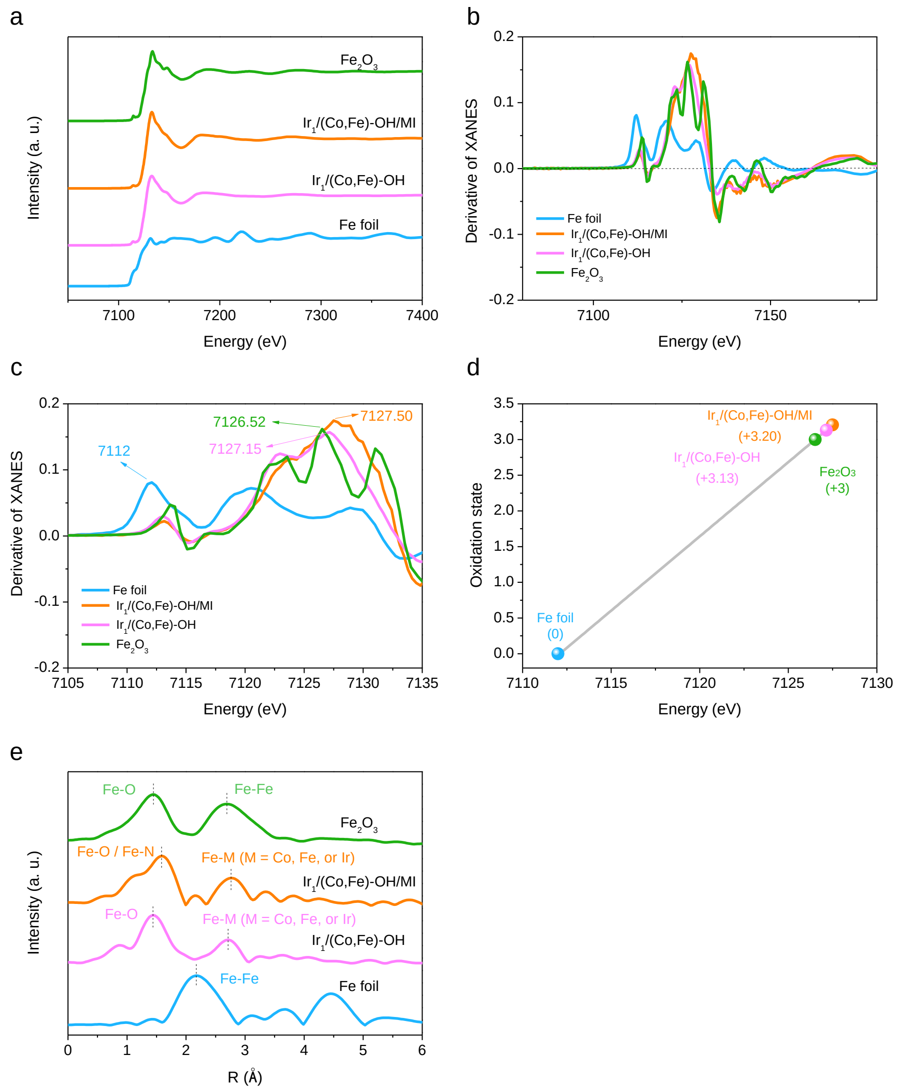

Supplementary Fig. 43 The results of Fe *K*-edge XAFS. a XANES spectra. **b** The first derivative spectra. **c** The enlargement of first derivative spectra. **d** Valence of various Fe species obtained from Fe *K*-edge XANES. **e** The normalized Fe *K*-edge FT-EXAFS spectra.

To measure the Fe *K*-edge XAFS spectra, we employed a calibration method similar to that illustrated in connection to Supplementary Fig. 16 and Supplementary Fig. 42. An Fe foil reference was used to correct for energy shifts between experimental and theoretical spectra caused by variations in beamlines across different synchrotron radiation facilities. The theoretical X-ray absorption edge energy (E_0, defined as the energy at the first maximum of the first-order derivative in the XANES spectrum) for the Fe *K*-edge was determined to be 7112 eV.

For 3d transition metals like Fe, the X-ray absorption edge energy (*i.e.*, E_0) in the normalized XANES spectra provides oxidation state information[22,23]. To determine the oxidation states of Fe in Ir_1/(Co,Fe)-OH/MI, Ir_1/(Co,Fe)-OH, Fe_2O_3, and the Fe foil, we first obtained their E_0 by finding the first maximum position energy in the first-order derivative of XANES spectra (Supplementary Fig. 43c). We then constructed a linear prediction curve using the E_0 of Fe_2O_3 and Fe foil as the horizontal axis (dependent variable/covariate) and their known oxidation states as the vertical axis (prediction variable) (Supplementary Fig. 43d). The oxidation states of Fe in Ir_1/(Co,Fe)-OH/MI and Ir_1/(Co,Fe)-OH were then estimated based on the measured E_0 and the above-mentioned prediction curve[22,23].

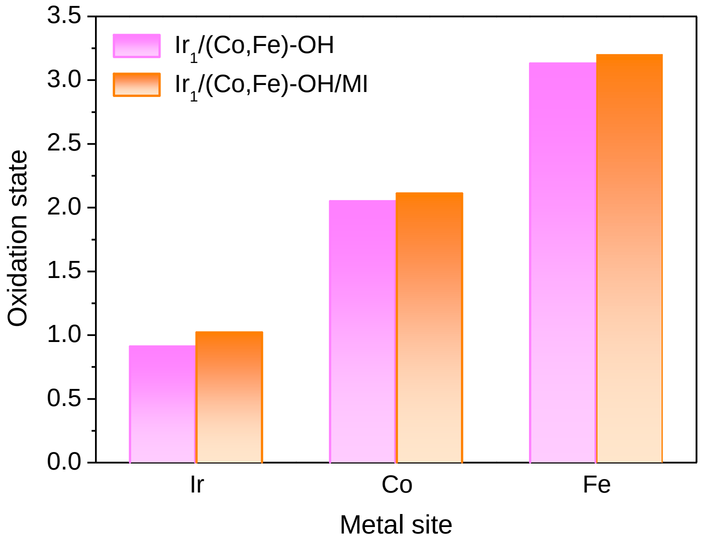

Supplementary Fig. 44 The oxidation state of Ir, Co, and Fe elements obtained from their XAFS spectra.

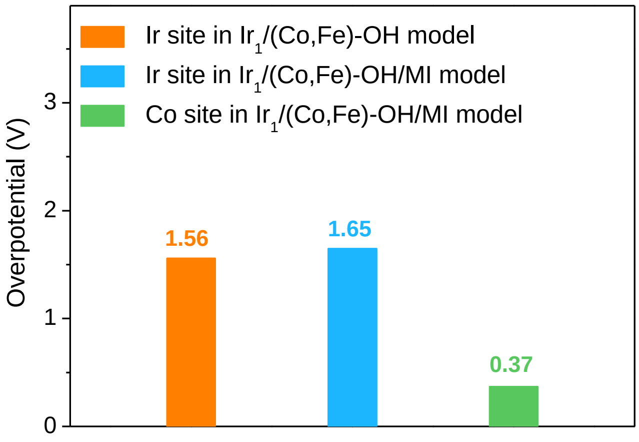

Supplementary Fig. 45 The calculated overpotentials of Ir and Co sites in Ir_1/(Co,Fe)-OH and Ir_1/(Co,Fe)-OH/MI models.

The calculated overpotentials of Ir and Co sites in the Ir_1/(Co,Fe)-OH and Ir_1/(Co,Fe)-OH/MI models are presented in Supplementary Fig. 45. Although the Ir site in the Ir_1/(Co,Fe)-OH/MI sample's model exhibits a slightly higher overpotential (1.65 V) compared to that of Ir_1/(Co,Fe)-OH (1.56 V), the Co site in the MI-containing model boasts a significantly lower overpotential (0.37 V). This substantial difference suggests that the Ir_1/(Co,Fe)-OH/MI sample's model has a lower overall overpotential, indicating higher overall OER activity than the Ir_1/(Co,Fe)-OH sample's model. These theoretical calculations are consistent with our experimental findings presented in Fig. 3b.

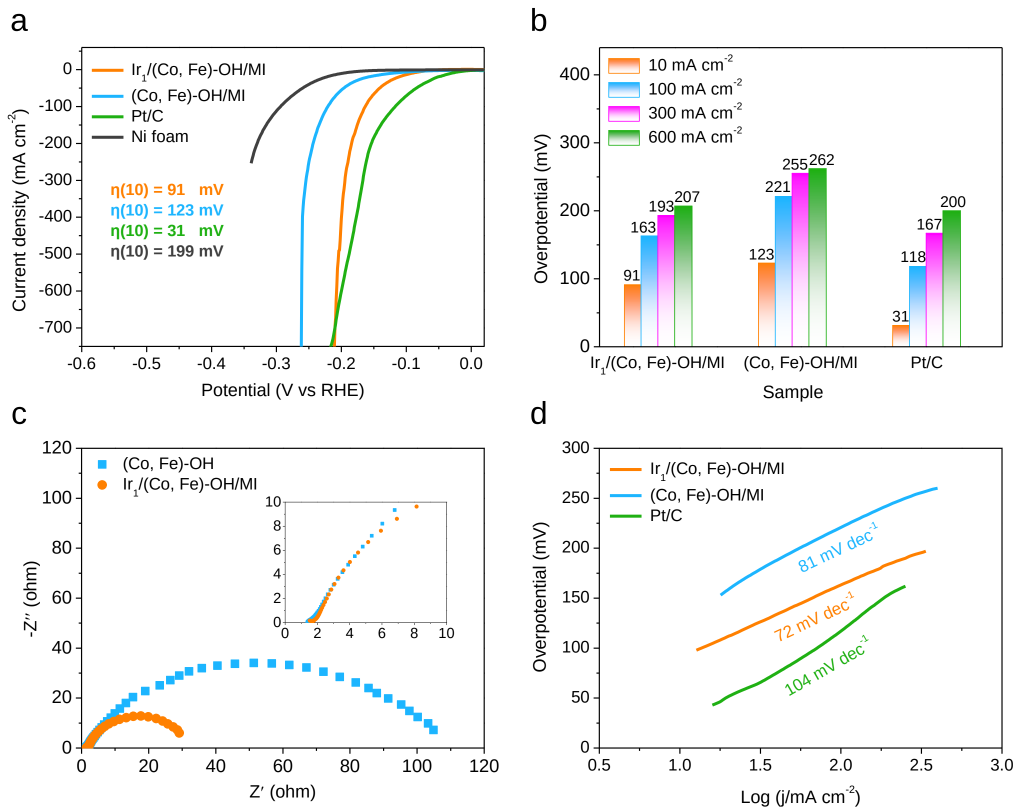

Supplementary Fig. 46 HER performances. **a** LSV curves. **b** Overpotentials at 10, 100, 300, and 600 mA cm^{-2}. **c** EIS spectra. **d** Tafel plots.

For HER tests, LSV characterization indicated that as-prepared catalysts exhibit high current densities (over 700 mA cm^{-2}) and good HER performance. Among them below the current density of 600 mA cm^{-2}, the electrochemical activities were in the order of 20% Pt/C > Ir_1/(Co,Fe)-OH/MI > (Co,Fe)-OH/MI > Ni foam (Supplementary Fig. 46a). At 10, 100, 300, and 600 mA cm^{-2}, the overpotentials of Ir_1/(Co,Fe)-OH/MI were 91, 163, 193, and 207 mV, respectively. These values were lower than those of (Co,Fe)-OH/MI (123, 221, 255, and 262 mV), but higher than those of 20% Pt/C (31, 118, 167, and 200 mV) at the same current densities (Supplementary Fig. 46b).

The Ir_1/(Co,Fe)-OH/MI had the lower transfer resistance (as evidenced by the smaller impedance arc) than (Co,Fe)-OH/MI, thus enabling faster HER reaction kinetics (Supplementary Fig. 46c). Thus, the Ir_1/(Co,Fe)-OH/MI obtained a Tafel slope of 72 mV dec^{-1}, a value below those measured for (Co,Fe)-OH/MI (81 mV dec^{-1}) and 20% Pt/C (104 mV dec^{-1}) (Supplementary Fig. 46d).

Based on the results of LSV and overpotentials (Supplementary Fig. 46a-b), it was known that Ir_1/(Co,Fe)-OH/MI exhibited lower HER performance than commercial 20% Pt/C catalyst. Therefore, except the the symmetrically electrolytic cell (*i.e.*, Ir_1/(Co,Fe)-OH/MI || Ir_1/(Co,Fe)-OH/MI), we also chose the commercial 20% Pt/C as HER electrode to prepare the asymmetrically electrolytic cell (*i.e.*, Ir_1/(Co,Fe)-OH/MI || 20% Pt/C) for overall water splitting, which aims to demonstrate the potential of Ir_1/(Co,Fe)-OH/MI catalyst as OER electrode (Fig. 5).

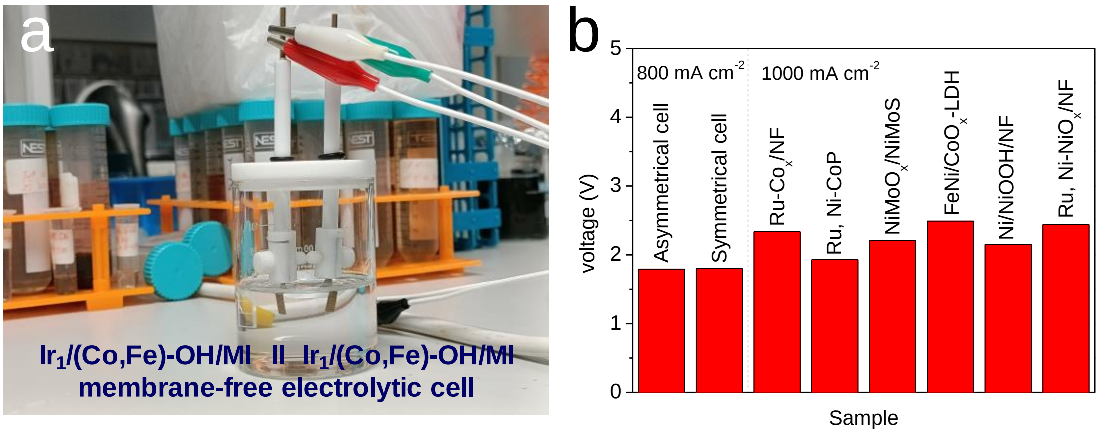

Supplementary Fig. 47 Performance of symmetric Ir_1/(Co,Fe)-OH/MI || Ir_1/(Co,Fe)-OH/MI membrane-free electrolytic cell. **a** Digital photograph of membrane-free electrolytic cell. **b** Comparison of overall water splitting voltage (1000 mA cm^{-2}) in reported OER electrocatalysts[33].

Supplementary Fig. 47a shows the two-electrode membrane-free electrolytic cell. This cell is used to measure the water splitting performance of Fig.5 in the main text.

The asymmetric Ir_1/(Co,Fe)-OH/MI || 20% Pt/C membrane-free electrolytic cell and the symmetric Ir_1/(Co,Fe)-OH/MI || Ir_1/(Co,Fe)-OH/MI membrane-free electrolytic cell demonstrate excellent overall water-splitting performance. They achieve low cell voltages of 1.80 V and 1.79 V, respectively, at a high current density of 800 mA cm^{-2}. This performance rivals that of many reported OER catalysts even at a higher current density of 1000 mA cm^{-2}.

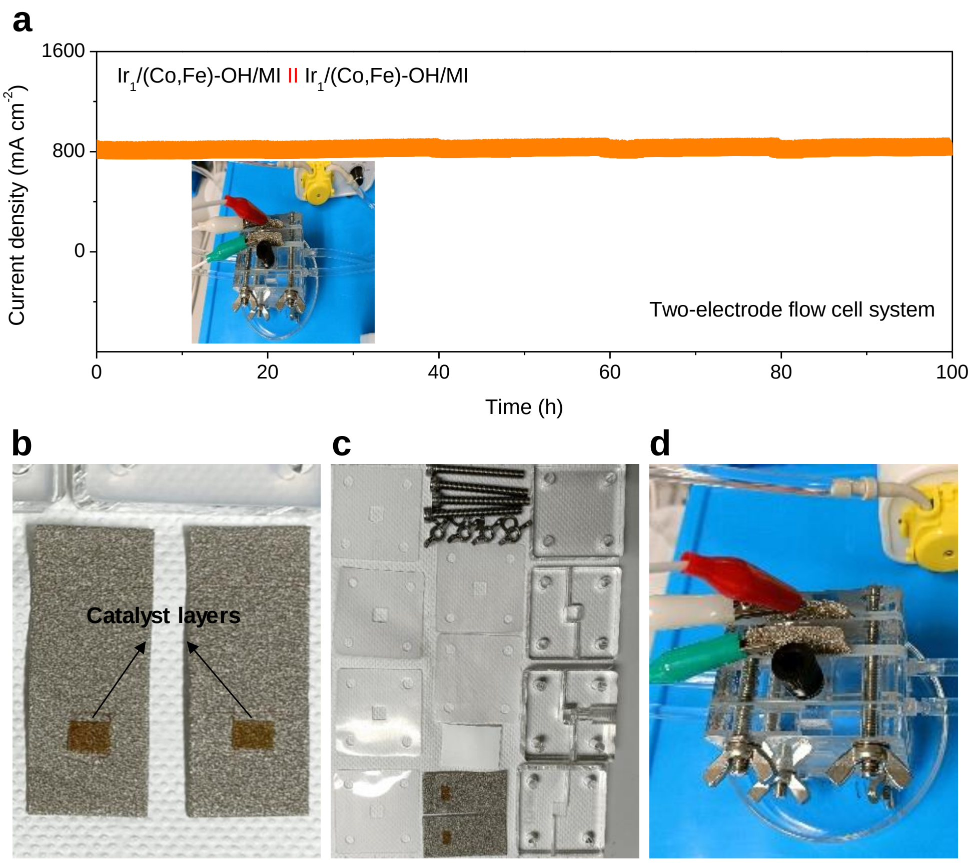

Supplementary Fig. 48 The stability test in the symmetric Ir_1/(Co,Fe)-OH/MI || Ir_1/(Co,Fe)-OH/MI flow electrolytic cell at 800 mA cm^{-2}. a Chronoamperometry measurements at 800 mA cm^{-2} for 100 h. **b** Ir_1/(Co,Fe)-OH/MI electrodes. **c** Components of a flow cell. **d** Photogarph of a flow cell.

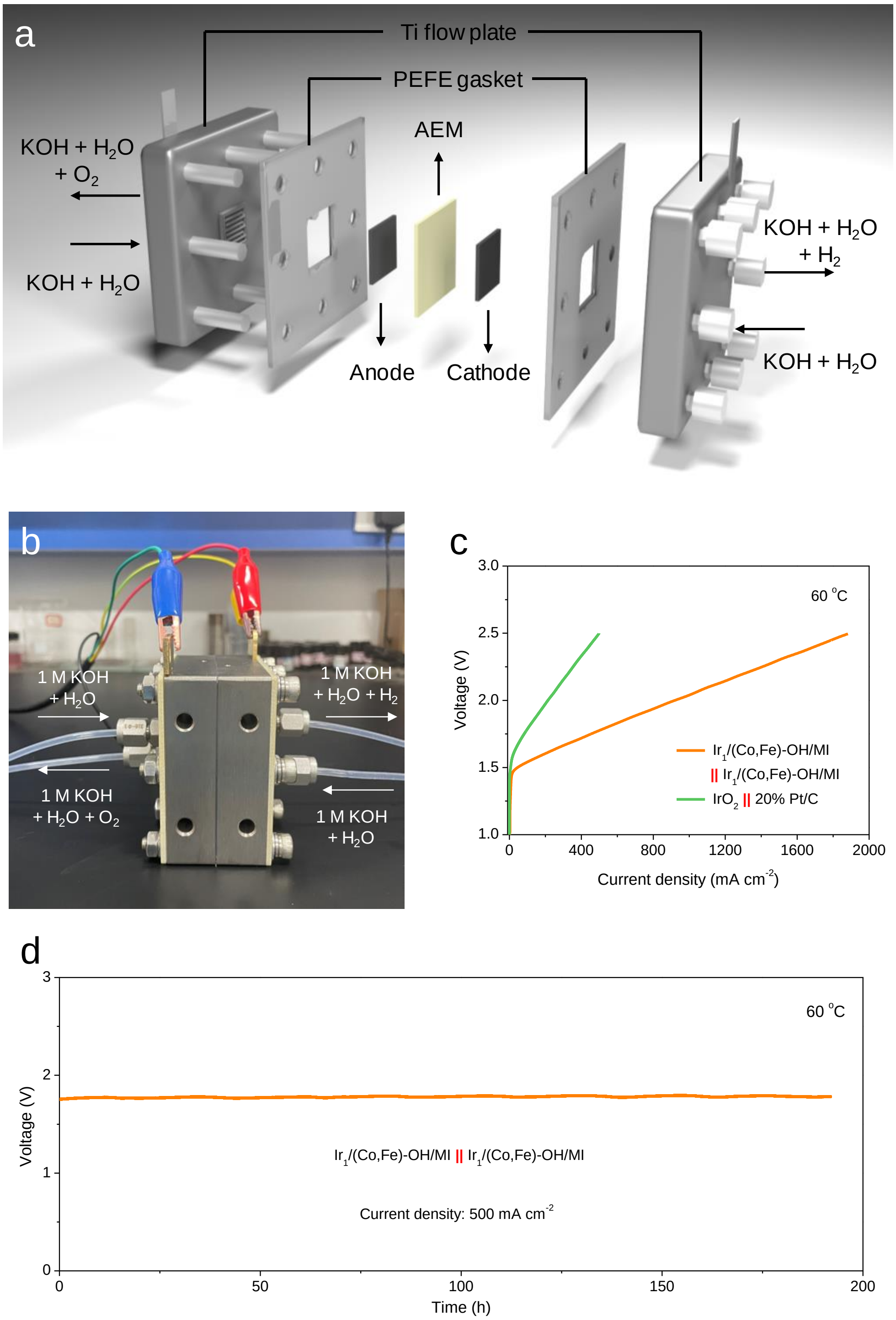

Supplementary Fig. 49 The performance of anion exchange membrane (AEM) water electrolyzer with Ir_1/(Co,Fe)-OH/MI electrodes. a Schematic diagram of the AEM water electrolyzer. **b** Digital photo of the AEM water electrolyzer. **c** Polarization curves of the AEM water electrolyzer in a 1.0 M KOH electrolyte at 60 °C. **d** Chronopotentiometry curve of the AEM water electrolyzer (Ir_1/(Co,Fe)-OH/MI || Ir_1/(Co,Fe)-OH/MI) at 500 mA cm^{-2}. Note: the area of work electrode was 2×2 cm^2. The voltage data displayed in c and d are 90% iR-compensated.

We also used the anion exchange membrane (AEM) water electrolyzer (denoted as Ir_1/(Co,Fe)-OH/MI || Ir_1/(Co,Fe)-OH/MI) to evaluate the Ir_1/(Co,Fe)-OH/MI sample's water splitting performance. In the Ir_1/(Co,Fe)-OH/MI || Ir_1/(Co,Fe)-OH/MI water

electrolyzer, the anode and cathode were Ir_1/(Co,Fe)-OH/MI electrodes, the separator was a commercial AEM (FAS-50), the electrolyte was 1.0 M KOH, and the test temperature was 60 ºC (Supplementary Fig. 49a,b). For comparison, we also assembled the IrO_2 || 20% Pt/C water electrolyzer.

The polarization curve showed that the Ir_1/(Co,Fe)-OH/MI || Ir_1/(Co,Fe)-OH/MI water electrolyzer possessed a lower voltage of 1.78 V at 500 mA cm^{-2}, than the commercial IrO_2 || 20% Pt/C water electrolyzer (2.50 V at 500 mA cm^{-2}) (Supplementary Fig. 49b). Compared with the performance of LDH-based AEM water electrolyzer reported in the literature, the performance of the Ir_1/(Co,Fe)-OH/MI || Ir_1/(Co,Fe)-OH/MI water electrolyzer is at the top level (Supplementary Table 13).

The chronopotentiometry measurement demonstrated that the Ir_1/(Co,Fe)-OH/MI || Ir_1/(Co,Fe)-OH/MI water electrolyzer ran stably over 150 h at a high current density of 500 mA cm^{-2}, with almost no performance degradation (Supplementary Fig. 49c). These results indicate that the Ir_1/(Co,Fe)-OH/MI as an advanced catalyst in electrocatalytic water splitting possesses a substantial potential for real-world applications.

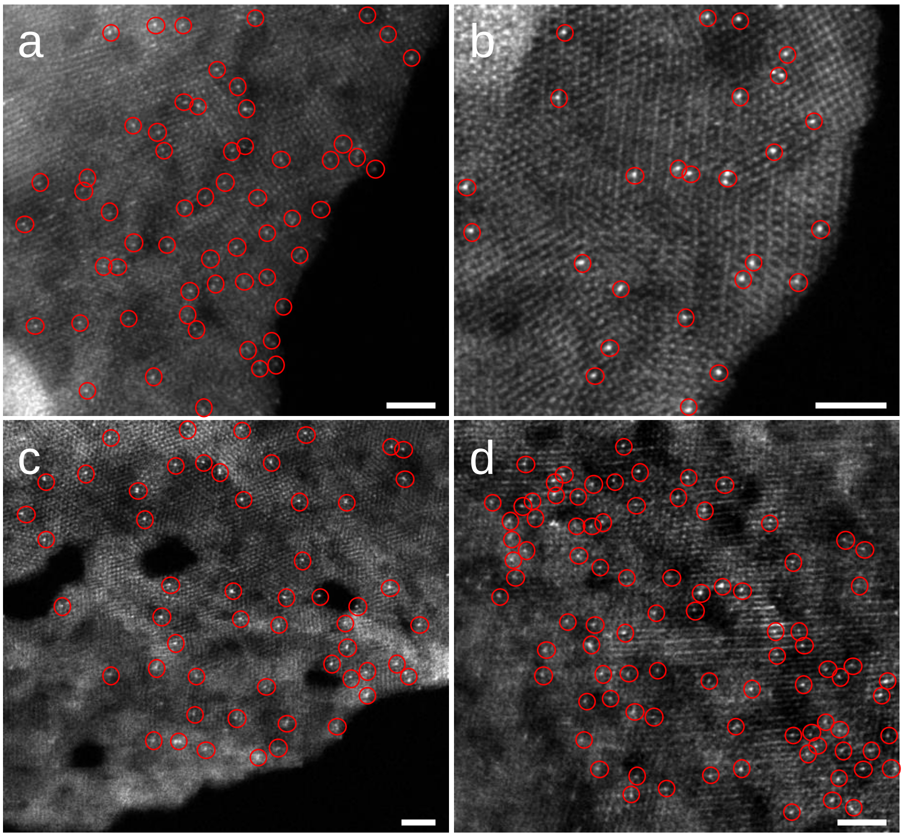

Supplementary Fig. 50 HAADF-STEM images of Pt_1/(Co,Fe)-OH/MI sample. a-d The HAADF-STEM images at different positions of Pt_1/(Co,Fe)-OH/MI sample. The scale bar is 2 nm.

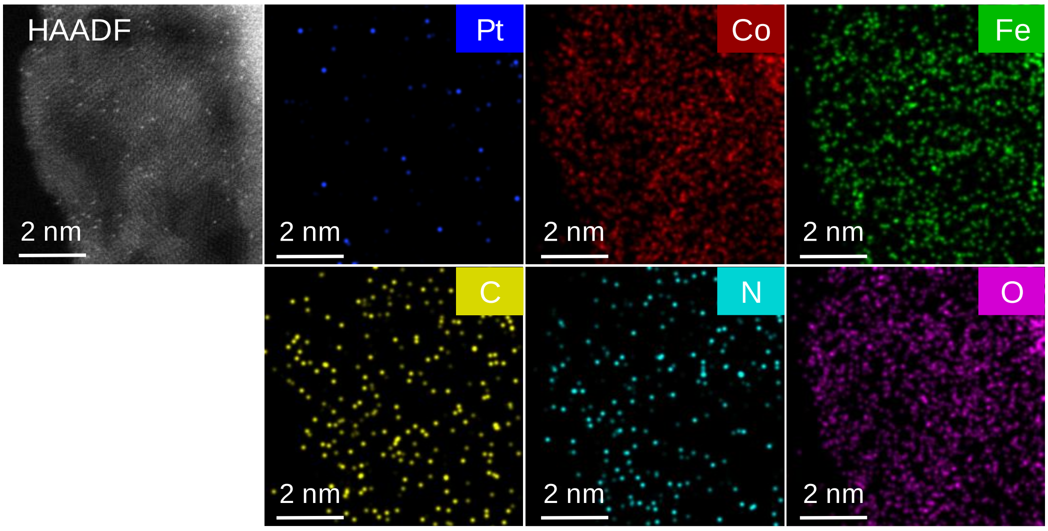

Supplementary Fig. 51 HAADF image and the corresponding element mappings of Pt_1/(Co,Fe)-OH/MI sample.

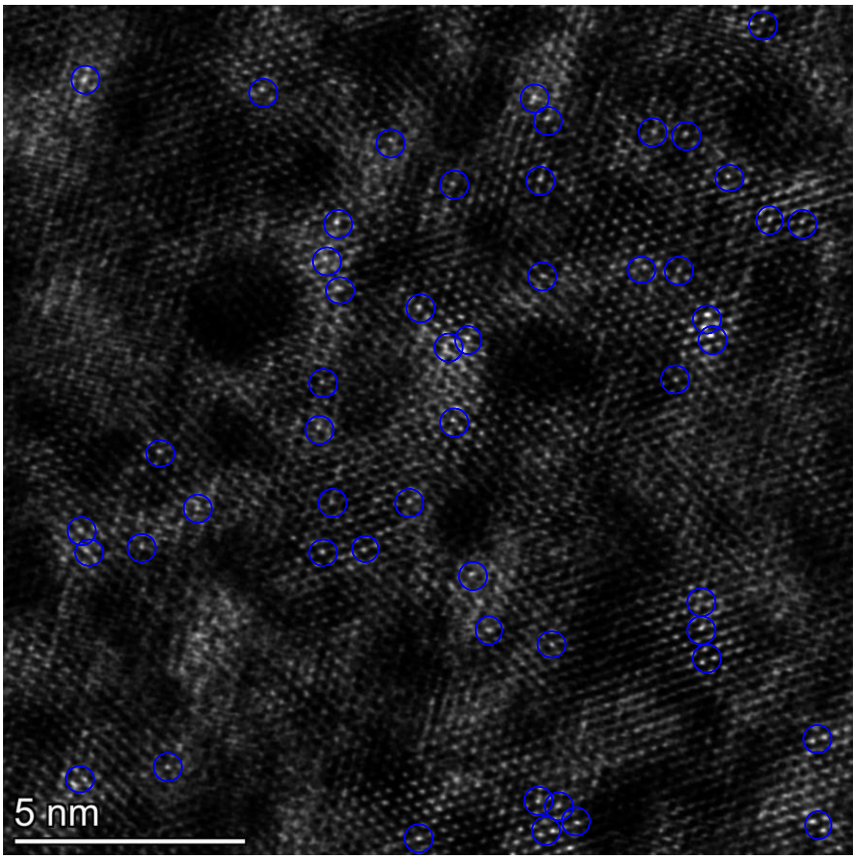

Supplementary Fig. 52 HAADF-STEM images of Pd_1/(Co,Fe)-OH/MI sample.

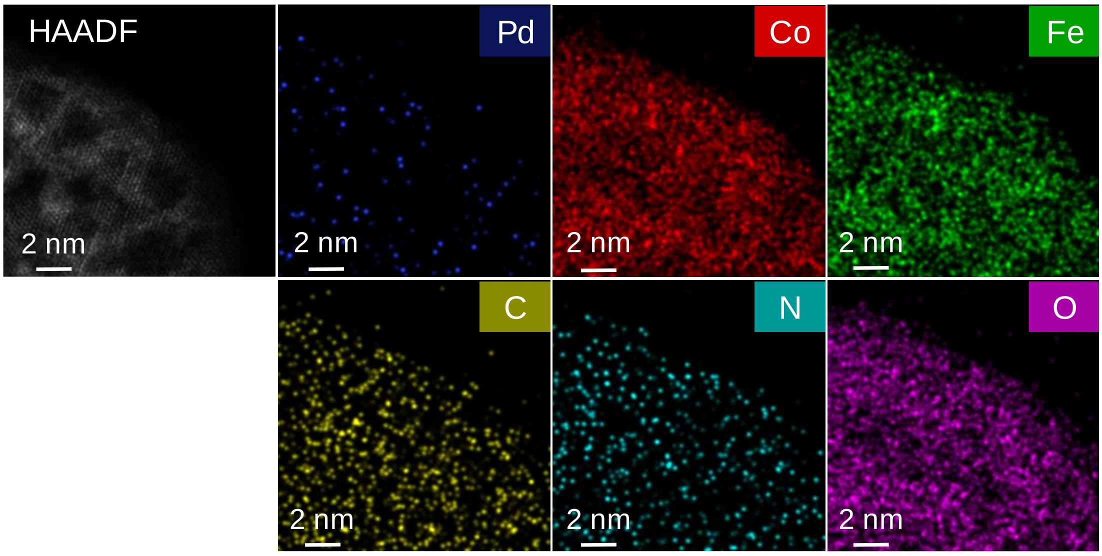

Supplementary Fig. 53 HAADF image and the corresponding element mappings of Pd_1/(Co,Fe)-OH/MI sample.

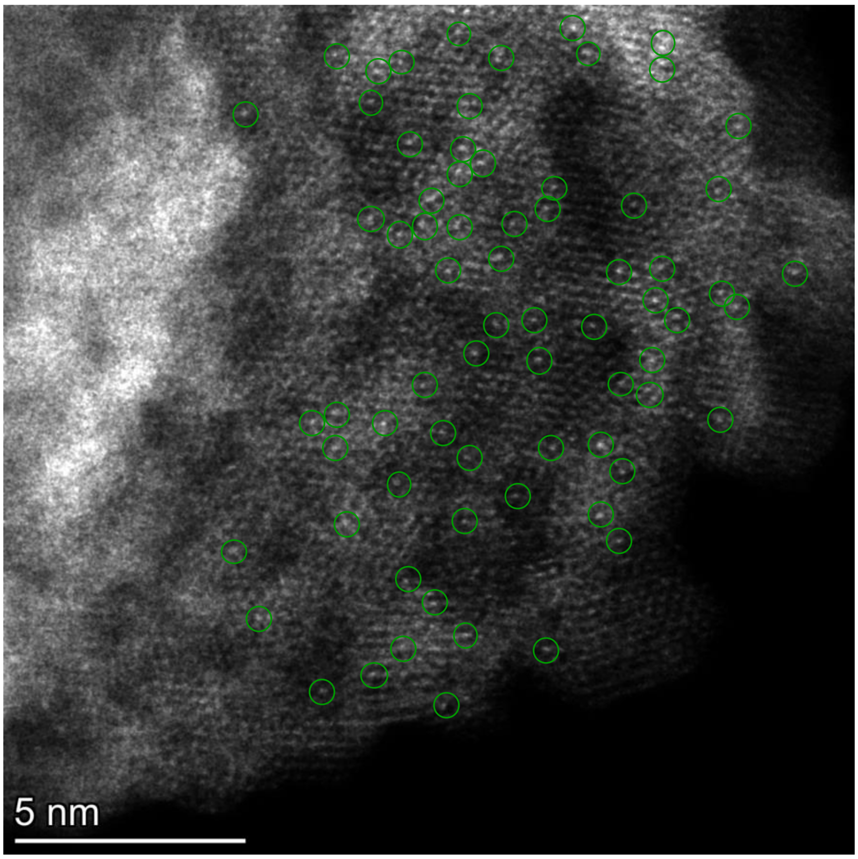

Supplementary Fig. 54 HAADF-STEM images of Ru_1/(Co,Fe)-OH/MI sample.

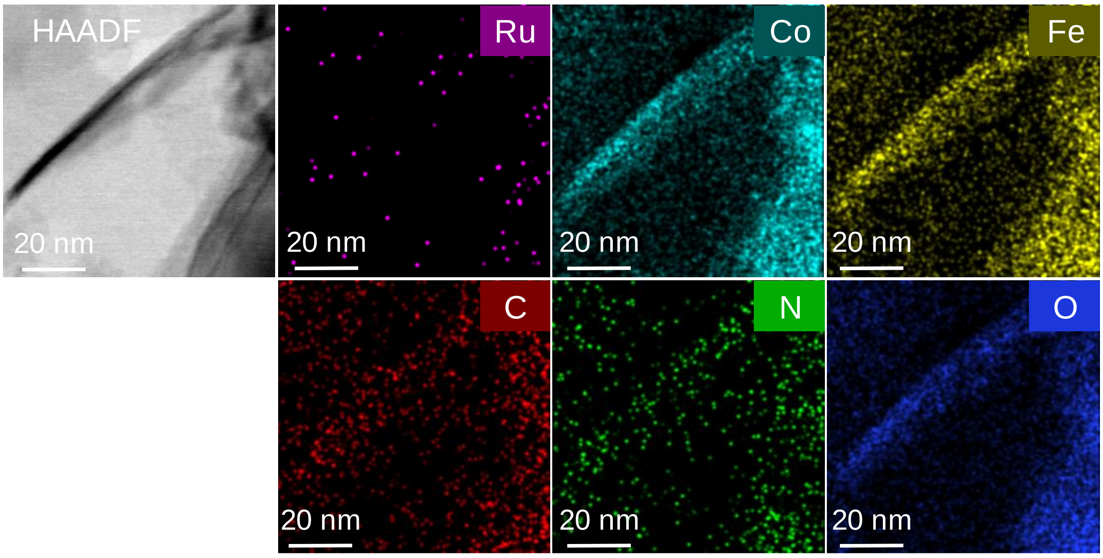

Supplementary Fig. 55 HAADF image and the corresponding element mappings of Ru_1/(Co,Fe)-OH/MI sample.

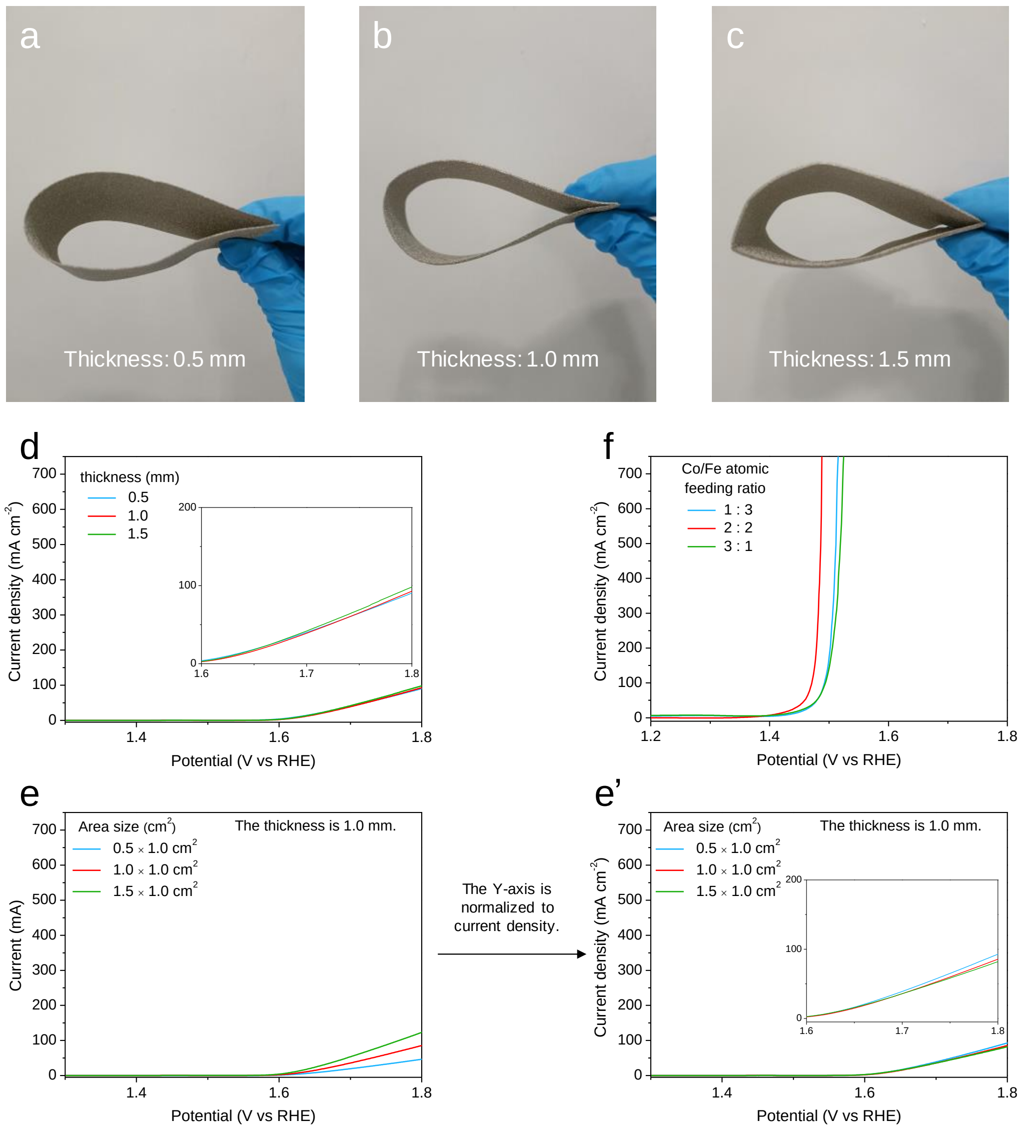

Supplementary Fig. 56 The influences of the thickness of Ni foam substrate and the Co/Fe atomic feeding ratio on OER performance. a-c Digital photos of Ni foam with different thicknesses. **d** LSV curves of Ni foam substrate with different thicknesses. The insert in d is a partial enlargement. **e,e'** LSV curves of Ni foam substrate with different area sizes. The insert in e' is a partial enlargement. **f** LSV curves of $Ir_1/(Co,Fe)$-OH/MI prepared using different Co/Fe atomic feeding ratios during the immersion process.

We investigated the impact of Ni foam substrate thickness on OER performance (Supplementary Fig. 56a−d). While Ni foam exhibits some OER activity above ~1.6 V (vs. RHE), its performance remains significantly lower than CoFe-based catalysts (Fig. 3a and Supplementary Fig. 56d). Slight increases in OER current density were observed in thicker substrates within the 1.7−1.8 V (vs. RHE) range.

We also investigated the impact of the cross-sectional area of the Ni foam substrate on OER performance (Supplementary Fig. 56e,e'). By increasing the cross-sectional area from 1×0.5 cm^2 to 1×1.5 cm^2, the Ni foam substrates (1.0 mm thickness) increased their OER current above ~1.6 V (vs. RHE) (Supplementary Fig. 56e). The currents (mA) in the Y-axis were normalized with respect to cross-sectional area to obtain the current densities (mA cm^{-2}). The current densities were similar for all cases (Supplementary Fig. 56e') and significantly lower than those of corresponding CoFe-based catalysts (Fig. 3a and Supplementary Fig. 56e'). This

article used a 1.0-mm-thick Ni foam with a cross-sectional area of 1×0.5 cm^2 to support the electrode used in the electrochemical tests, except the electrodes (thickness: 1.0 mm; area: 2×2 cm^2) used in the AEM water electrolyzer.

We also explored the influence of the Co/Fe atomic feeding ratio. The Ir_1/(Co,Fe)-OH/MI catalyst achieved optimal OER performance at a Co/Fe feeding ratio of 2:2. ICP-MS analysis confirmed a true atomic ratio of 1.75:1 Co/Fe in this sample (Supplementary Table 2).

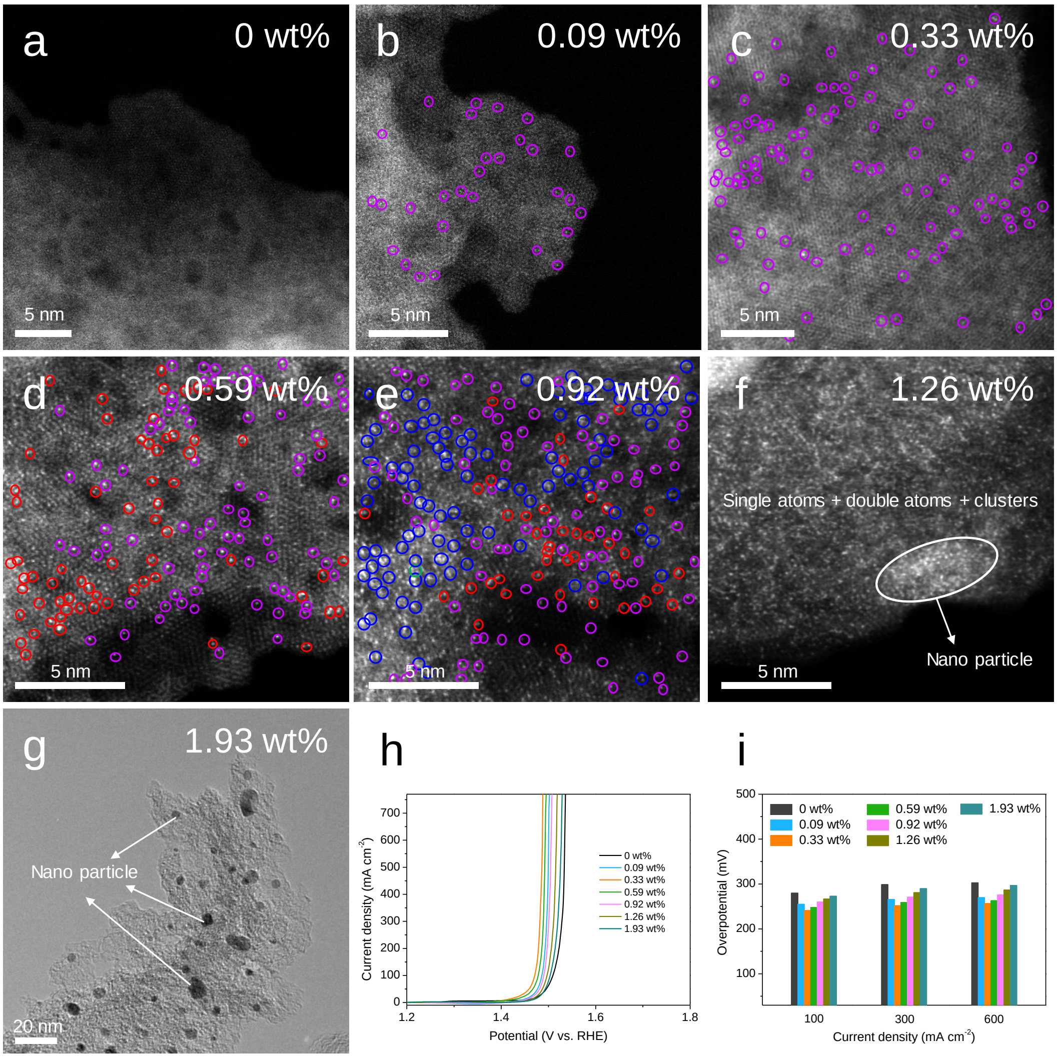

Supplementary Fig. 57 The morphology characterization and electrochemical tests of Ir$_1$/(Co,Fe)-OH/MI samples with different Ir mass fractions. a-f HAADF-STEM images. **g** TEM image. **h** LSV curves. **i** Overpotentials. Note: the mass fractions of Ir atoms were determined by ICP-MS tests.

We explored the effects of different Ir mass fractions on OER performance. When the mass fraction of Ir atoms in Ir$_1$/(Co,Fe)-OH/MI was less than 0.33 wt%, Ir atoms existed as isolated atoms (bright dots marked by pink circles) (Supplementary Fig. 57a-c). When the mass fraction of Ir atoms in Ir$_1$/(Co,Fe)-OH/MI was 0.59 wt%, Ir atoms existed as isolated atoms and double atoms (marked by red circles) (Supplementary Fig. 57d). When the mass fraction of Ir atoms in Ir$_1$/(Co,Fe)-OH/MI was 0.92 wt%, Ir atoms existed as isolated atoms, double atoms, and clusters (marked by blue circles) (Supplementary Fig. 57e). When the mass fraction of Ir atoms was 1.26 wt%, Ir atoms existed as isolated atoms, double atoms, clusters, and nanoparticles (marked by white circle) (Supplementary Fig. 57f). When the mass fraction of Ir atoms was 1.93 wt%, a lot of Ir nanoparticles were formed (Supplementary Fig. 57g).

With the increase of Ir mass fraction, the OER performance presented a trend of an increase, followed by a decrease. When the mass fraction of Ir atoms was 0.33 wt%, the OER performance was optimized (Supplementary Fig. 57h,i).

Supplementary Tables

Supplementary Table S1 Comparisons of digital photographs of samples prepared in different immersion solutions.

	The composition of immersion solution	Immersion Time	Digital photos of samples	
			Before immersion	After immersion
1 #	Co^{2+}, Ethylene glycol, H_2O	20 h	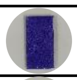	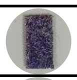
2 #	Fe^{3+}, H_2O	20 h	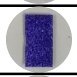	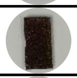
3 #	Fe^{3+}, Co^{2+}, H_2O	20 h	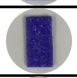	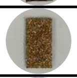
4 #	Fe^{3+}, Ethylene glycol, H_2O	20 h	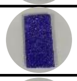	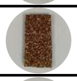
5 #	Ethylene glycol, H_2O	20 h	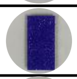	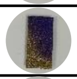
6 #	Co^{2+}, H_2O	20 h	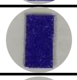	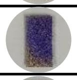
7 #	Co^{2+}, Fe^{3+}, Ethylene glycol, H_2O	20 h	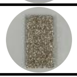	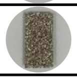
8 #	Co^{2+}, Fe^{3+}, Ethylene glycol, H_2O	20 h	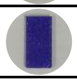	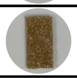

Note: The initial pH values of all immersion solutions were adjusted to ~4.5. All immersion solutions contained 350 uL of Ir^{3+} solution.

Based on these controlled experiments, we can draw the following key conclusions:

1) The flocculation of Fe^{3+} played a crucial role in converting Co-MI to CoFe-based hydroxide (Group 1#, Group 4#, and Group 5# experiments).

2) CoFe-based hydroxides formed when Co^{2+} and Fe^{3+} co-existed in immersion solutions without ethylene glycol. However, the distribution of the CoFe-based hydroxide on Ni foam was uneven (Group 2#, Group 3#, and Group 6# experiments). Experiments in Group 3# and Group 8# further suggest that ethylene glycol promotes uniform distribution of CoFe-based hydroxide on Ni foam.

3) The presence of Co-MI was essential for strong adhesion of CoFe-based hydroxides to the Ni foam substrate (Group 7# and Group 8# experiments).

Supplementary Table S2 The elemental analysis of Ir_1/(Co,Fe)-OH/MI sample through ICP-MS measurements. ICP-MS test value (ppb) Co: 29.45; Fe: 16.00; Ir: 0.36.

**Quantity**	**Value**
Mass percentage of Co	27.18 wt%
Mass percentage of Fe	14.77 wt%
Mass loading of Ir	0.33 wt%
Atomic ratio of Co/Fe	1.75 : 1
Atomic ratio of Co/Fe/Ir	266.84 : 152.98 : 1

Note: The mass of Ir_1/(Co,Fe)-OH/MI dissolved into 4 mL of 3wt% HNO_3 was 0.65 mg. Then, 20 μL of the solution was diluted to 30 mL for ICP-MS test.

Supplementary Table S3 Elemental analysis of Ir_1/(Co,Fe)-OH sample following ICP-MS characterization. ICP-MS test value (ppb) Co: 259.55; Fe: 132.96; Ir: 2.91.

**Quantity**	**Value**
Mass percentage of Co	26.65 wt%
Mass percentage of Fe	13.65 wt%
Mass loading of Ir	0.30 wt%
Atomic ratio of Co/Fe	1.85 : 1
Atomic ratio of Co/Fe/Ir	291.05 : 157.38 : 1

Note: The mass of Ir_1/(Co,Fe)-OH dissolved into 4 mL of 3 wt% HNO_3 was 3.9 mg. Then, 30 μL of the solution was added into 30 mL for ICP-MS test.

Supplementary Table S4 Comparisons of OER performance for metal hydroxides-based single-atom catalysts.

**Catalyst**	**Tafel slope (mV dec^{-1})**	**Overpotential (mV) @ 10 mA cm^{-2}**	**Reference**
Ir_1/(Co,Fe)-OH/MI	24	179	This study
Ru_1/NiFe-LDH	31	189	*Nat. Commun.* 12, 4587 (2021)
Ru_1/CoFe-LDH	25	194	*Energy Environ. Sci.* 15, 4048 (2022)
Ru_1/CoFe-LDH	39	198	*Nat. Commun.* 10, 1711 (2019)
Ru_1/NiFe-LDH	98.1	196	*ACS Catal.* 13, 2771 (2023)
Ru_1/CoFeNi-LDH	40	205	*Adv. Energy Mater.* 11, 2002816 (2021)
Ru_1/NiFe-LDH	36	194	*Chem. Eng. J.* 446, 136962 (2022)
Ru_1/NiFe-LDH	50.2	230	*Nanoscale* 12, 9669 (2020)
$Ir_1/Ni(OH)_2$	58	223	*Nano Res.* 15, 10014–10020 (2022).
$Ir_1/Ni(OH)_2$	58.4	235	*Chem. Eng. J.* 395, 125149 (2020)
$Ir_1/Ni(OH)_2$	78	260	*Nano Lett.* 22, 3832 (2022)
Ir_1/CoOOH	32	200	*Nat. Commun.* 13, 2473 (2022)
Au_1/NiFeOOH	36	237	*J. Am. Chem. Soc.* 140, 3876 (2018)

Supplementary Table S5 Comparisons of OER performance for various other electrocatalysts.

Catalyst	Tafel slope (mV dec^{-1})	Overpotential (mV) @ 10 mA cm^{-2}	Reference
Ir_1/(Co,Fe)-OH/MI	24	179	This study
δ-FeOOH	68	390	*Adv. Mater.* 30, 1 (2018)
Fe_1Ni_2-BDC	42	260	*ACS Energy Lett.* 4, 285 (2019)
NiFe-LDH	46	251	*ACS Catal.* 9, 6027 (2019)
Rh/NiFeRh-LDH	40	230	*Nano Lett.* 20, 136 (2020)
Ni_2Fe_1-O	39	244	*Adv. Energy Mater.* 8, 1 (2018)
FeP/Ni_2P	22.7	190	*Nat. Commun.* 9, 1 (2018)
r-NiFeOOH	40	270	*ACS Energy Lett.* 3, 1515 (2018)
Cu_3N	118	286	*ACS Energy Lett.* 4, 747 (2019)
Ni_2P-VP_2	49	280	*Adv. Mater.* 31, 1901174 (2019)
CoV-LDH	44	250	*Energy Environ. Sci.* 11, 1736 (2018)
CoFeZr oxides	54.2	248	*Adv. Mater.* 31, 1901439 (2019)
Ni-P-B	70.6	263	*Energy Environ. Sci.* 13, 102 (2020)
$Ni_2P_4O_{12}$	156	280	*Adv. Mater.* 30, 1705045 (2018)
Co_3O_4/CoFe oxides	61	297	*Adv. Mater.* 30, 1801211 (2018)
F-Co_2B	32	320	*Energy Environ. Sci.* 12, 2443 (2019)
Ni-ZIF/Ni-B	101	226	*Adv. Energy Mater.* 9, 1902714 (2019)
NiFeMo-LDH	35	238	*ACS Energy Lett.* 3, 546 (2018)
np-Ir/NiFeO	29.6	197	*Nat. Commun.* 11, 2701 (2020)
w-$Ni(OH)_2$	33	237	*Nat. Commun.* 10, 2149 (2019)
Ir/NiO	38	215	*J. Am. Chem. Soc.* 142, 7425 (2020)
FeOOH(Se)/IF	54	287	*J. Am. Chem. Soc.* 141, 7005 (2019)
CoO/Co_3O_4	54	260	*Angew. Chem. Int. Ed.* 59, 6929 (2020)
CoFe LDH-Ar	37.8	266	*Angew. Chem. Int. Ed.* 56, 5867 (2017)
Ni/$Ni(OH)_2$	53	270	*Adv. Mater.* 32, 1906915 (2020)
W_2N/WC	122.8	320	*Adv. Mater.* 32, 1905679 (2020)
HCM@Ni-N	76	304	*Adv. Mater.* 31, 1904548 (2019)
δ-FeOOH NSs/N	69	265	*Adv. Mater.* 30, 1803144 (2018)
P-Co_3O_4	51.6	280	*Adv. Energy Mater.* 10, 1902521 (2020)
Fe-CoOOH/G	37	330	*Adv. Energy Mater.* 7, 1602148 (2017)
N-Fe_2PO_{5-x}-OT	27.2	235	*Adv. Funct. Mater.* 28, 1801397 (2018)
NiFe-OH-F	42.9	243	*Nano Lett.* 19, 530 (2019)

Supplementary Table S6 The A_{ECSA} of Ir_1/(Co,Fe)-OH/MI, (Co,Fe)-OH/MI, Ir_1/(Co,Fe)-OH, (Co,Fe)-OH, and IrO_2 samples.

**Sample**	**A_{ECSA} (per ECSA cm^2)**
Ir_1/(Co,Fe)-OH/MI	14.08
(Co,Fe)-OH/MI	12.42
Ir_1/(Co,Fe)-OH	22.38
(Co,Fe)-OH	24.62
IrO_2	3.92
Ni foam	1.00

Supplementary Table S7 Comparisons of mass activity based on noble metal for metal hydroxides-based single-atom catalysts tested in OER.

**Catalyst**	**Mass activity based on noble metal (A $g_{nobel\ metal}^{-1}$) @ 250 mV overpotential**	**Reference**
Ir_1/(Co,Fe)-OH/MI	99967.97	This study
Ru_1/NiFe-LDH	14547.41 (@ 240 mV)	*Nat. Commun.* 12, 4587 (2021)
Ru_1/CoFe-LDH	19243.98	*Nat. Commun.* 10, 1711 (2019)
Ru_1/NiFe-LDH	257.28	*ACS Catal.* 13, 2771−2779 (2023)
Ru_1/CoFeNi-LDH	43962.03	*Adv. Energy Mater.* 11, 2002816 (2021)
Ru_1/NiFe-LDH	< 25.86	*Nanoscale* 12, 9669-9679 (2020)
Ir_1/Ni(OH)$_2$	1562.13	*Chem. Eng. J.* 395, 125149 (2020)
Ir_1/Ni(OH)$_2$	10930.56	*Nano Lett.* 22, 3832–3839 (2022)
Ir_1/CoOOH	14530.93	*Nat. Commun.* 13, 2473 (2022)
Au_1/NiFeOOH	3571.43	*J. Am. Chem. Soc.* 140, 3876–3879 (2018)

Supplementary Table S8 The elemental analysis of (Co,Fe)-OH/MI sample through ICP-MS measurements. ICP-MS test value (ppb) Co: 134.96; Fe: 74.93.

**Quantity**	**Value**
Mass percentage of Co	23.21 wt%
Mass percentage of Fe	12.89 wt%
Co/Fe Atomic ratio	1.80 : 1

Note: The mass of (Co,Fe)-OH/MI dissolved into 4 mL of 3 wt% HNO_3 was 0.7 mg. Then, 100 μL of the solution was added into 30 mL of 3 wt% HNO_3 for ICP-MS test.

Supplementary Table S9 The elemental analysis of (Co,Fe)-OH sample through ICP-MS measurements. ICP-MS test value (ppb) Co: 305.24; Fe: 157.91.

**Quantity**	**Value**
Mass percentage of Co	26.57 wt%
Mass percentage of Fe	13.75 wt%
Co/Fe Atomic ratio	1.84 : 1

Note: The mass of (Co,Fe)-OH dissolved into 4 mL of 3 wt% HNO_3 was 4.6 mg. Then, 30 μL of the solution was added into 30 mL for ICP-MS test.

Supplementary Table S10 The equivalent circuit diagram and the detailed values of R_s and R_{ct} for Ir_1/(Co,Fe)-OH/MI, (Co,Fe)-OH/MI, Ir_1/(Co,Fe)-OH, (Co,Fe)-OH, and IrO_2 at an OER overpotential of 250 mV.

**Equivalent circuit**	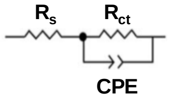	
**Catalyst**	**R_s (Ohmic resistance, Ω)**	**R_{ct} (charge transfer resistance, Ω)**
Ir_1/(Co,Fe)-OH/MI	1.53	0.91
(Co,Fe)-OH/MI	1.55	4.14
Ir_1/(Co,Fe)-OH	1.64	4.21
(Co,Fe)-OH	1.81	6.10
IrO_2	1.57	104.44

R_s represents the intrinsic resistance of the electrode material, electrolyte, current collector, leads, and their contact resistances.

R_{ct} arises from electronic and ionic resistances at the electrode-electrolyte interface. This resistance is influenced by the catalyst's activity, charge transport kinetics during redox reactions, electrolyte-electrode wettability, electrode morphology, and conductivity. Additionally, ion transfer distance and temperature can also affect R_{ct}.

Supplementary Table S11 Summary of overall water splitting data for various catalysts (1 M KOH electrolyte and 10 mA cm^{-2}).

**Cell**	**Cell voltage (V) @ 10 mA cm^{-2}**	**Reference**
Ir_1/(Co,Fe)-OH/MI ‖ 20% Pt/C	1.44	This study
Ir_1/(Co,Fe)-OH/MI ‖ Ir_1/(Co,Fe)-OH/MI	1.51	
$RuIrO_x$ ‖ $RuIrO_x$	1.47	*Nat. Commun.* 10, 4875 (2019)
Ru_1/D-NiFe LDH ‖ Ru_1/D-NiFe LDH	1.44	*Nat. Commun.* 12, 4587 (2021)
Ni-Fe NPs ‖ Ni-Fe NPs	1.47	*Nat. Commun.* 10, 5599 (2019)
CoMoNiS-NF ‖ CoMoNiS-NF	1.54	*J. Am. Chem. Soc.* 141, 10417 (2019)
R-$NiCo_2O_4$ ‖ R-$NiCo_2O_4$	1.61	*J. Am. Chem. Soc.* 140, 13644 (2018)
CoP/NCNHP ‖ CoP/NCNHP	1.64	*J. Am. Chem. Soc.* 140, 2610 (2018)
CoMnO@CN ‖ CoMnO@CN	1.50	*J. Am. Chem. Soc.* 137, 14305 (2015)
RuCu NSs ‖ RuCu NSs	1.49	*Angew. Chem. Int. Ed.* 58, 13983 (2019)
Co/β-Mo_2C@N-CNTs ‖ Co/β-Mo_2C@N-CNTs	1.64	*Angew. Chem. Int. Ed.* 58, 4923 (2019)
VOOH/NF ‖ VOOH/NF	1.62	*Angew. Chem. Int. Ed.* 56, 573 (2017)
Co_3O_4-MTA ‖ Co_3O_4-MTA	1.63	*Angew. Chem. Int. Ed.* 56, 1324 (2017)
Cu@NiFe LDH ‖ Cu@NiFe LDH	1.54	*Energy Environ. Sci.* 10, 1820 (2017)
EG/$Co_{0.85}$Se/NiFe LDH ‖ EG/$Co_{0.85}$Se/NiFe LDH	1.67	*Energy Environ. Sci.* 9, 478 (2016)
MoO_3/Ni-NiO ‖ MoO_3/Ni-NiO	1.55	*Adv. Mater.* 32, 2003414 (2020)
Ni/$Ni(OH)_2$ ‖ Ni/$Ni(OH)_2$	1.59	*Adv. Mater.* 32, 1906915 (2020)
W_2N/WC ‖ W_2N/WC	1.58	*Adv. Mater.* 32, 1905679 (2020)
Cr-doped FeNi-P/NCN ‖ Cr-doped FeNi-P/NCN	1.50	*Adv. Mater.* 31, 1900178 (2019)
δ-FeOOH NSs/NF ‖ δ-FeOOH NSs/NF	1.62	*Adv. Mater.* 30, 1803144 (2018)
CoS_x@Cu_2MoS_4-MoS_2/NSG ‖ CoS_x@Cu_2MoS_4-MoS_2/NSG	1.60	*Adv. Energy Mater.* 10, 1903289 (2020)
Ni-ZIF/Ni-B ‖ Ni-ZIF/Ni-B	1.54	*Adv. Energy Mater.* 10, 1902714 (2019)
Mo-Co_9S_8@C ‖ Mo-Co_9S_8@C	1.56	*Adv. Energy Mater.* 10, 1903137 (2019)
Co@N-CS/N-HCP ‖ Co@N-CS/N-HCP	1.545	*Adv. Energy Mater.* 9, 1803918 (2019)
NiFe LDH@NiCoP ‖ NiFe LDH@NiCoP	1.57	*Adv. Funct. Mater.* 28, 1706847 (2018)
sNiCoP/NF ‖ sNiCoP/NF	1.58	*Nano Lett.* 16, 7718 (2016)
CoFe@NiFe LDH ‖ CoFe@NiFe LDH	1.59	*Appl. Catal. B* 253, 131 (2019)
S-CoO_x ‖ S-CoO_x	1.63	*Nano Energy* 71, 104652 (2020)
$Zn_{1-x}Fe_x$-LDH ‖ $Zn_{1-x}Fe_x$-LDH	1.62	*Small* 14, 1803638 (2018)
NiCoFeB ‖ NiCoFeB	1.81	*Small* 15, 1804212 (2019)

Supplementary Table S12 Summary of overall water splitting literature data for various catalysts (1 M KOH electrolyte at various current densities).

**Cell**	**Cell voltage (V,)**	**Reference**
Ir_1/(Co,Fe)-OH/MI || 20% Pt/C	1.80 @ 800 mA cm^{-2}	This study
Ir_1/(Co,Fe)-OH/MI || Ir_1/(Co,Fe)-OH/MI	1.79 @ 800 mA cm^{-2}	
NFN-MOF/NF || NFN-MOF/NF	1.96 @ 500 mA cm^{-2}	*Adv. Energy Mater.* 8, 1801065 (2018)
B-NFN-MOF/NF || B-NFN-MOF/NF	2.02 @ 500 mA cm^{-2}	*Adv. Energy Mater.* 8, 1801065 (2018)
Pt-C/NF || IrO_2/NF	2.02 @ 500 mA cm^{-2}	*Adv. Energy Mater.* 8, 1801065 (2018)
CoFe-LDH || CoFe-LDH	1.83 @ 500 mA cm^{-2}	*Energy Environ. Sci.* 15, 4048 (2022)
Ni-Mo-B HF || Ni-Mo-B HF	1.88 @ 500 mA cm^{-2}	*Adv. Funct. Mater.* 32, 2107308 (2021)
Stainless steel || Ni foam	2.08 @ 500 mA cm^{-2}	*Energy Environ. Sci.* 11, 2858 (2018)
Pt/C || IrO_2	2.01 @ 500 mA cm^{-2}	*Energy Environ. Sci.* 13, 86 (2020)
Stainless steel || Ni foam	2.12 @ 500 mA cm^{-2}	*Energy Environ. Sci.* 13, 86 (2020)

Supplementary Table S13 Comparisons of AEM water electrolyzer performance for various metal hydroxide-based catalysts (1 M KOH electrolyte).

AEM water electrolyzer	Operating temperature (°C)	Mass loading of catalysts (mg cm^{-2})	Electrolyzer voltage (V)	Reference
Ir_1/(Co,Fe)-OH/MI || Ir_1/(Co,Fe)-OH/MI	60	0.7	1.54 @ 100 mA cm^{-2}<br>1.78 @ 500 mA cm^{-2}<br>2.04 @ 1000 mA cm^{-2}	This study
NiFe-LDH || NiFe-LDH	—	0.5	2.13 @ 250 mA cm^{-2}	*Nano Res.* 16, 2286–2293 (2023).
Pt_1-Mn,Fe-Ni-LDH || Pt_1-Mn,Fe-Ni-LDH	60	~8.0	1.79 @ 500 mA cm^{-2}	*ACS Nano* 18, 16222−16235 (2024).
Pt@S–NiFe LDH || S–NiFe LDH	65	>1.0	1.62 @ 100 mA cm^{-2}	*Adv. Mater.* 35, 2208209 (2023).
CuNiFe-LDH/NFP || $NiFe_2O_4$/NFP	50	~2.5	1.85 @ 947 mA cm^{-2}	*Appl. Catal. B: Environ.* 340, 123187 (2024).
NiFeCoP || NiFeCo-LDH	53~55	8.3 (NiFeCo-LDH)<br>7.2 (NiFeCoP)	1.75 @ 500 mA cm^{-2}	*Appl. Catal. B: Environ.* 294, 120246 (2021).
Monolayer-NiFe-LDH/NF || Monolayer-NiFe-LDH/NF	50	3.0	1.69 @ 1000 mA cm^{-2}	*ACS Appl. Mater. Interfaces* 13, 37179–37186 (2021).
Ni_3S_4@$Ni(OH)_2$ || Ni_3S_4@$Ni(OH)_2$	Room temperature	>1.0	1.84 @ 500 mA cm^{-2}	*J. Colloid Interface Sci.* 654, 66–75 (2024).
Cr-NiFe-LDH || NiMoCo	40	0.9 (Cr-NiFe-LDH)	2.11 @ 1000 mA cm^{-2}	*Small* 18, 2200303 (2022).
NiFe LDH-NiS || Ni	80~85 (30wt% KOH)	—	2.01 @ 400 mA cm^{-2}	*Adv. Energy Mater.* 11, 2102353 (2021).
$Co(OH)_x$/Ag/$Co(OH)_2$ || Pt/C	50	—	1.80 @ 600 mA cm^{-2}	*J. Alloy. Compd.* 889, 161674 (2021).
Cu/γ-NiOOH || Cu/γ-NiOOH	50	~4	1.81 @ 400 mA cm^{-2}	*Chem Catalysis* 3, 100552, (2023).
$Ni_xCo_{1-x}Se_yOOH$ || Pt/C	60	2 (NixCo1-xSeyOOH)<br>0.2 (Pt)	1.75 @ 1000 mA cm^{-2}	*Adv. Mater.* 33, 2103812 (2021).

References

1. Kresse, G. & Furthmüller, J. Efficiency of Ab-initio total energy calculations for metals and semiconductors using a plane-wave basis set. Comp. Mater. Sci. 6, 15–50 (1996).
2. Kresse, G. & Hafner, J. Ab initio molecular dynamics for liquid metals. Phys. Rev. B 47, 558–561 (1993).
3. Kresse, G. & Hafner, J. Ab initio molecular-dynamics simulation of the liquid-metal–amorphous-semiconductor transition in germanium. Phys. Rev. B 49, 14251–14269 (1994).
4. Kresse, G. & Furthmüller, J. Efficient iterative schemes for Ab initio total-energy calculations using a plane-wave basis set. Phys. Rev. B 54, 11169–11186 (1996).
5. Kresse, G. & Joubert, D. From ultrasoft pseudopotentials to the projector augmented-wave method. Phys. Rev. B. 59, 1758–1775 (1999).
6. Blöchl, P. E. Projector augmented-wave method. Phys. Rev. B. 50, 17953–17979 (1994).
7. Perdew, J. P., Burke, K. & Ernzerhof, M. Generalized gradient approximation made simple. Phys. Rev. Lett. 77, 3865–3868 (1996).
8. Monkhorst, H. J. & Pack, J. D. Special points for brillouin-zone integrations. Phys. Rev. B 13, 5188–5192 (1976).
9. Grimme, S., Antony, J., Ehrlich, S. & Krieg, H. A consistent and accurate ab initio parametrization of density functional dispersion correction (DFT-D) for the 94 elements H-Pu. J. Chem. Phys. 132, 154104 (2010).
10. Nørskov, J. K., et al. Origin of the overpotential for oxygen reduction at a fuel-cell cathode. J. Phys. Chem. B 108, 17886–17892 (2004).
11. Man, I. C., et al. Universality in oxygen evolution electrocatalysis on oxide surfaces. ChemCatChem 3, 1159–1165 (2011).
12. Shi, H. et al. A sodium-ion-conducted asymmetric electrolyzer to lower the operation voltage for direct seawater electrolysis. Nat. Commun. 14, 3934 (2023).
13. Peng, S., et al. Necklace-like multishelled hollow spinel oxides with oxygen vacancies for efficient water electrolysis. J. Am. Chem. Soc. 140(42), 13644–13653 (2018).
14. Kang, J., et al. Valence oscillation and dynamic active sites in monolayer NiCo hydroxides for water oxidation. Nat. Catal. 4, 1050–1058 (2021).
15. Wang, X., et al. Pivotal role of reversible NiO_6 geometric conversion in oxygen evolution. Nature 611, 702–708 (2022).
16. Fan, W., et al. Rational design of heterogenized molecular phthalocyanine hybrid single-atom electrocatalyst towards two-electron oxygen reduction. Nat. Commun. 14, 1426 (2023).
17. Liu, F., et al. Avoiding sabatier's limitation on spatially correlated Pt–Mn atomic pair sites for oxygen electroreduction. J. Am. Chem. Soc. 145(46), 25252–25263 (2023).

18. Meng, H., et al. Optimizing electronic synergy of atomically dispersed dual-metal Ni–N_4 and Fe–N_4 sites with adjacent Fe nanoclusters for high-efficiency oxygen electrocatalysis. Energy Environ. Sci. 17, 704–716 (2024).

19. Huang, W., et al. Ligand modulation of active sites to promote electrocatalytic oxygen evolution. Adv. Mater. 34, 2200270 (2022).

20. Zhao, J., et al. Balancing loading mass and gravimetric capacitance of NiCo–layered double hydroxides to achieve ultrahigh areal performance for flexible supercapacitors. Adv. Powder Mater. 3, 100151 (2024).

21. Zhao, J., et al. Sub-nanometer-scale fne regulation of interlayer distance in Ni–Co layered double hydroxides leading to high-rate supercapacitors. Nano Energy 76, 105026 (2020).

22. Zhao, H. et al. The role of Cu_1-O_3 species in single-atom Cu/ZrO_2 catalyst for CO_2 hydrogenation. Nat. Catal. 5, 818–831 (2022).

23. Gu, J., Hsu, C.-S., Bai, L., Chen, H.M. & Hu, X. Atomically dispersed Fe^{3+} sites catalyze efficient CO_2 electroreduction to CO. Science 364, 1091–1094 (2019).

24. Zhu, Y. et al. Iridium single atoms incorporated in Co_3O_4 efficiently catalyze the oxygen evolution in acidic conditions. Nat. Commun. 13, 7754 (2022).

25. Baroudi, K. et al. Structure and properties of α-$NaFeO_2$-type ternary sodium iridates. J. Solid. State Chem. 210, 195 (2014).

26. Agrestini, S. et al. Nature of the magnetism of iridium in the double perovskite Sr_2CoIrO_6. Phys. Rev. B 100, 014443 (2019).

27. Feng, H. et al. Room-temperature ferrimagnetism of anti-sitedisordered Ca_2MnOsO_6. Phys. Rev. Mater. 3, 124404 (2019).

28. Li, N. et al. Identification of the active-layer structures for acidic oxygen evolution from 9R-$BaIrO_3$ electrocatalyst with enhanced iridium mass activity. J. Am. Chem. Soc. 143, 18001–18009 (2021).

29. Liu, Y. et al. Operando identification of dual active sites in Ca_2IrO_4 nanocrystals with yttrium substitutions boosting acidic oxygen evolution reaction. ACS Energy Lett. 7, 3798–3806 (2022).

30. Lu, X. & Zhao, C. Electrodeposition of hierarchically structured three-dimensional nickel–iron electrodes for efficient oxygen evolution at high current densities. Nat. Commun. 6, 6616 (2015).

31. Tang, C., Cheng, N.Y., Pu, Z.H., Xing, W. & Sun, X.P. NiSe nanowire film supported on nickel foam: An efficient and stable 3D bifunctional electrode for full water splitting. Angew. Chem. Int. Ed. 54, 9351–9355 (2015).

32. Hou, Y., Lohe, M.R., Zhang, J., Liu, S.H., Zhuang, X.D. & Feng, X.L. Vertically oriented cobalt selenide/NiFe layered-double-hydroxide nanosheets supported on exfoliated graphene foil: An efficient 3D electrode for overall water splitting. Energy Environ. Sci. 9, 478–483 (2016).

33. Mu, X., et al. Breaking the symmetry of single-atom catalysts enables an extremely low energy barrier and high stability for large-current-density water splitting. Energy Environ. Sci. 15, 4048–4057 (2022).
